# Highly disordered nanoporous carbons for enhanced energy storage in supercapacitors

**DOI:** 10.1038/s41467-026-71520-x

**Published:** 2026-04-20

**Authors:** Xinyu Liu, Robert D. Hunter, Zhen Xu, El Hassane Lahrar, Céline Merlet, Clare P. Grey, Maria-Magdalena Titirici, Alexander C. Forse

**Affiliations:** 1 Yusuf Hamied Department of Chemistry, University of Cambridge, Cambridge, UK; 2 Department of Chemical Engineering, Imperial College London, London, UK; 3 Sorbonne Université, CNRS, Physicochimie des Électrolytes et Nanosystèmes Interfaciaux, Paris, France; 4 Réseau sur le Stockage Électrochimique de l’Énergie (RS2E), Fédération de Recherche CNRS 3459, Amiens, France; 5 CIRIMAT, Université de Toulouse, Toulouse INP, CNRS, Toulouse, France; 6 Advanced Institute for Materials Research (WPI-AIMR), Tohoku University, Sendai, Japan

**Keywords:** Supercapacitors, Electrochemistry, Electrochemistry

## Abstract

There has been a lack of clear principles for designing nanoporous carbons with enhanced performance in supercapacitors due to their structural complexity. Our recent NMR and Raman spectroscopy studies of a series of commercial nanoporous carbons show that carbons with smaller graphene-like domains have higher capacitance. In this study, we demonstrate that low-temperature synthesis provides a promising route for producing highly disordered nanoporous carbons with enhanced gravimetric and volumetric capacitance. NMR spectroscopy measurements provide unique insights by simultaneously probing local structural order and ion adsorption capacities, revealing that carbons with smaller graphene-like domain sizes and higher ion adsorption capacities generally have better capacitive performance. We finally show that the capacitance of a nanoporous carbon can be predicted directly from the NMR spectra of electrolyte-soaked electrodes. Our findings provide a strategy that can be extended to various carbon precursors and synthesis routes for developing energy storage materials with enhanced capacitance.

## Abstract (web summary)

Recent work has shown that structural disorder is a key descriptor of capacitance in nanoporous carbons. Here, the authors demonstrate that low-temperature synthesis produces carbons with smaller graphene-like domains and higher ion adsorption capacity, leading to enhanced capacitance.

## Introduction

Electrochemical double layer capacitors (EDLCs, a type of supercapacitor) are high-performance energy storage devices due to their fast charging rates and cycle stability^1,2^. The electrode materials in commercial EDLCs are typically nanoporous carbons, which consist of disordered graphene-like domains that form the pore walls of a porous three-dimensional structure^3–7^. While numerous efforts have been devoted to optimise the pore size^3,8,9^, functional groups^10,11^, surface area^12,13^ and local structural graphitisation degree^14^ to improve the performance, clear design principles for optimising nanoporous carbons to enhance capacitance have yet to be established due to their complex structures.

Nanoporous carbons in supercapacitors possess high Brunner-Emmett-Teller (BET) surface area and pore volume, normally achieved by activation^15^. Among various activation methods, chemical activation using potassium hydroxide (KOH) is widely used in academic studies^16,17^, as it allows the controlled and efficient development of microporosity at lower temperatures with higher yields compared to physical activation^17^. The combination of hydrothermal pretreatment of biomass and chemical activation has also proven to be a promising route to synthesising high-performance nanoporous carbons for EDLCs^18,19^. During the KOH activation process, KOH decomposes into K_2_CO_3_ around 400 °C, which further breaks down into K_2_O and CO_2_ at temperatures above 700 °C. Pore formation occurs through: (i) redox reactions between carbon and KOH/K_2_CO_3_ that etch the carbon framework, consuming carbon atoms and creating void spaces; (ii) gaseous CO_2_ and CO evolution that opens pores. This combined chemical etching and physical activation leads to high BET surface areas and development of pores around 2 nm diameter^17,18,20^. In our recent studies of 20 commercial microporous activated carbons, we showed that the degree of disorder, and specifically the sizes of the graphene-like domains, correlates with their capacitance in organic electrolyte^21^. Carbons with smaller graphene-like domains were found to have larger capacitances. This was revealed through solid-state nuclear magnetic resonance (NMR) spectroscopy experiments combined with mesoscopic simulations^21^. In the NMR measurements, the sizes of the graphene-like domains were measured via the Δ*δ* values of adsorbed electrolyte ions in electrolyte-soaked carbon samples^21–27^, where;

1 $$\Delta \delta \,\left({ppm}\right)={{\delta }_{{in}-{pore}}-\delta }_{{neat}\,{electrolyte}}$$

where ${\delta }_{{neat\; electrolyte}}$ is the chemical shift of the neat electrolyte and ${\delta }_{{in}-{pore}}$ is the chemical shift of the “in-pore” resonance, which arises from ions located within the nanopores of the carbon material^10,21,23–25,27^. This Δ*δ* value observed in these samples originates predominantly from “ring current effects”, whereby the delocalised electrons in the aromatic carbon rings give rise to a local magnetic field which is experienced by the in-pore ions as a nucleus independent chemical shift (NICS)^22,28,29^. The magnitude of the NICS (and therefore Δ*δ*) depends on the sizes of the graphene-like domains in the porous carbon, the pore size distribution of the carbon, and adsorption effects which influence the average carbon-ion separation^22,30^. Our previously reported NMR simulation approach^21,22,30^ provides a simple method to model these effects, accounting for both the pore sizes and ion-carbon interactions, therefore allowing a representative graphene-like domain size to be extracted from the NMR spectrum. The approach assumes a carbon slit-pore model comprised of polyaromatic hydrocarbon pore walls, and uses the experimentally acquired pore size distributions of the carbons from gas sorption, as well as carbon-ion density profiles obtained from molecular dynamics simulations^30^. Additionally, the integral of the in-pore resonance quantifies the ion adsorption capacity within nanopores in the absence of applied potential. This approach provides a more direct measure of ion accessible porosity than gas physisorption analysis, as the latter relies on the assumption that pores accessible to small gas molecules at measurement temperatures (*e.g*., 77 K for N_2_) are equally accessible to the larger electrolyte ions at room temperature.

Beyond NMR spectroscopy, other spectroscopic probes of carbon disorder have been developed, the most widely and routinely used being Raman spectroscopy. Raman spectra of disordered nanoporous carbons exhibit two major features. First, the D band (between 1330 and 1350 cm^−1^) is attributed to the A_1g_ breathing mode of the six membered carbon rings in a graphene sheet, and only appears when there is structural disorder. Second, the G band (between 1580 and 1590 cm^−1^) is assigned to the E_2g_ stretching mode of the sp^2^ bonds^31^. In Raman measurements, the graphene-like domain size in nanoporous carbons is characterised by the intensity ratio of the D band to the G band (*I*_D_/*I*_G_, peak height)^31–34^, with more disordered carbons having smaller *I*_D_/*I*_G_ (peak height) values for stage 2 carbons within the 3-stage model proposed by Robertson and Ferrari^31^. Our recent Raman spectroscopy study of a large family of carbons supported the findings from NMR that carbons with smaller graphene-like domains have higher capacitances in symmetric EDLCs in a conventional organic electrolyte 1 M tetraethylammonium tetrafluoroborate in acetonitrile (1 M NEt_4_BF_4_/ACN)^21,32^. Upon thermal annealing of the commercial activated carbons, we observed that the sizes of the graphene-like domains increased with increasing annealing temperature, with the capacitance decreasing. While TEM has been used to visualise topological defects in disordered carbons^35^, such imaging provides only localised views without the quantitative disorder metrics essential for establishing structure-performance correlations.

In pursuit of high capacitance activated carbons, we targeted the synthesis of highly disordered carbons with small graphene-like domains. Indeed, previous modelling studies^4,7^, as well as experimental work on carbide-derived carbons^3,14^, showed that lower temperature syntheses give rise to carbons with more defects and smaller graphene-like domains. While low-temperature-treated carbons exhibit smaller graphene-like domains^36^, previous studies of supercapacitor electrodes have focused on optimising the pore structure and have largely overlooked domain size^18^ -a factor shown to be a primary determinant of capacitance performance. We here synthesise a range of highly disordered activated carbons and find that their high capacitances correlate with their small graphene-like domain sizes, while the electrolyte accessibility to the carbon pores also plays an important role, presenting a fundamental shift from porosity-centred to disorder-centred design^37,38^. Our work shows that low-temperature synthesis is a promising method for producing nanoporous activated carbons with a more disordered local structure and enhanced energy storage in EDLCs and demonstrates how fundamental mechanistic insights can transform materials synthesis strategy, even using well-established chemical processes, offering a clear principle for designing nanoporous carbons with better performance.

## Results and discussions

### Low-temperature synthesised nanoporous carbons with enhanced capacitance

Low temperature syntheses of glucose-derived activated carbons were carried out in order to synthesise highly disordered nanoporous carbons (See Methods). We emphasise that “low-temperature synthesis” refers specifically to the effective KOH activation temperature range of 600–750 °C, below which insufficient carbonisation may lead to decreased material performance. Importantly, both the hydrothermal carbonisation and the KOH chemical activation steps were carried out at the same temperature for each carbon, with the nomenclature HTC-X representing a hydrothermal carbon prepared at X °C. A relatively long carbonisation and activation time (6 h) was applied for sufficient carbonisation and activation in the low-temperature synthesis.

**Fig. 1:**
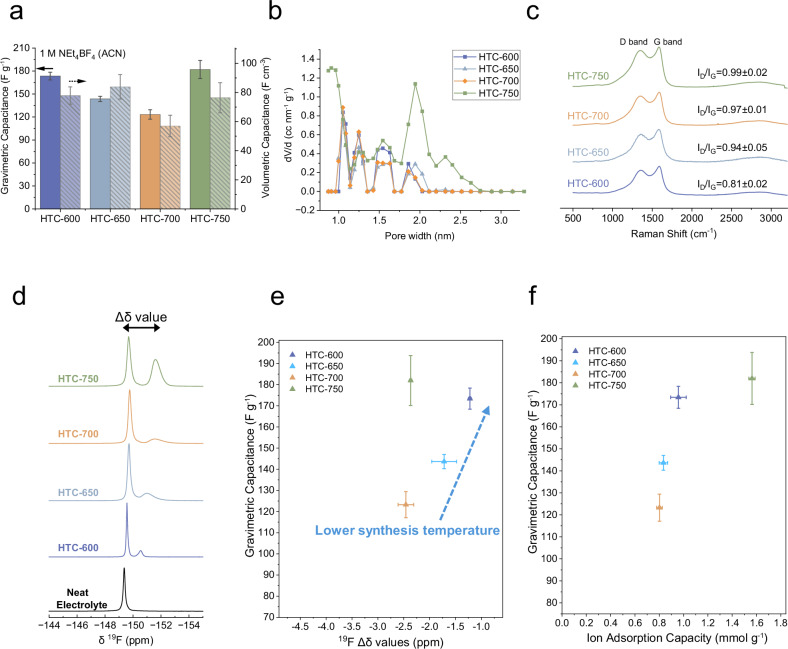
Characterisation of the synthesised nanoporous carbons. **a** Gravimetric and volumetric capacitances of low-temperature synthesised carbons measured at 0.05 A g^−1^ in 1 M NEt_4_BF_4_ /ACN, with the masses of the electrodes shown in Table S4. **b** Pore size distributions of low-temperature synthesised carbons calculated based on 2D non-local density functional theory (2D-NLDFT)^40^ analysis (slit pore model) of N_2_ isotherms at 77 K, with BET surface area, pore volume and average pore size presented in Table S1. **c** Raman spectra of low-temperature synthesised carbons (532 nm). See Supporting Information and our previous work^32^ for details on deconvolution of Raman spectra. **d** ^19^F MAS NMR spectra (14.1 T, 5 kHz MAS) of the synthesised carbons soaked with 1 M NEt_4_BF_4_ /ACN electrolyte. **e** Plot of gravimetric capacitance and ^19^F Δ*δ* values derived from **d**), with the in-pore chemical shifts taken as the weighted average for carbons showing multiple in-pore environments. **f** Relationship between gravimetric capacitance at 0.05 A g^−1^ in 1 M NEt_4_BF_4_/ACN and ion adsorption capacity.

An initial series of activated nanoporous carbons showed increasing gravimetric capacitances as the synthesis temperature was decreased from 700 to 600 °C, both at 0.05 A g^−1^ as well as at higher current densities up to 2 A g^−1^ (Fig. 1a, Figures S1 and S2), with the rectangular cyclic voltammograms and predominantly linear constant charge-discharge curves indicating double-layer capacitance behaviour. Interestingly, HTC-750 deviated from this trend and showed similar capacitances to HTC-600 (Fig. 1a). Gas physisorption results showed that HTC-600, HTC-650 and HTC-700 have similar BET surface areas of 1411, 1245, and 1581 m^2^ g^−1^, respectively, and similar pore size distributions (Fig. 1b, Figure S3 and Table S1), with all carbons being predominantly microporous (with pores below 2 nm diameter). In contrast, the surface area of HTC-750 is noticeably larger (2689 m^2^ g^−1^), with clear differences in the pore size distribution compared to the other carbons (Fig. 1b). Additional characterisation using X-ray photoelectron spectroscopy (XPS) measurements revealed high surface oxygen content ( ~ 15–20 at. %) for all synthesised carbons (Figure S4 and Table S2), though the oxygen content did not show any apparent correlation with gravimetric capacitance (Figure S4), similar to our previous findings^21^. We also note that the oxygen content in the bulk is much lower (less than 10 wt%) (Table S3). While oxygen-containing functional groups can affect the point of zero charge (PZC) of the nanoporous carbons and thus influence their performance in aqueous electrolytes^10^, these effects may be less significant in the organic electrolyte (1 M NEt_4_BF_4_ /ACN) used in the current study^39^. Additionally, the ^13^C MAS (magic angle spinning) NMR spectrum of HTC-650, the sample with the highest surface %O content, showed that the carbon atoms are predominantly sp^2^ hybridised, with no clear signature of sp^3^ content observed (Figure S5). Overall, these results suggest that while chemical functionalities and porosity are often considered key parameters for capacitance, the enhanced capacitance observed for the low temperature carbons reported here likely arises from other factors.

To study the synthesised carbons’ local structural order degree, Raman spectroscopy measurements of carbon powders were carried out (Fig. 1c). Deconvolution of the Raman spectra suggested a trend of decreasing sizes of graphene-like fragments (*I*_D_/*I*_G_ intensity ratio values decreasing, Stage 2 of Ferrari and Robertson model)^31,34^ as the synthesis temperature was decreased from 750 to 600 °C (Fig. 1c). A broad feature at 2300 to 3200 cm^−1^ is also observed for all the four synthesised carbons, which is attributed to the modulated 2D, D + D’ and 2D’ bands^41^. The observed temperature-dependent evolution of domain sizes aligns with previous findings^4,32,36^, where carbons synthesised at lower temperatures have smaller graphene-like domains due to kinetic limitations that hinder extensive graphitisation. We therefore propose that the capacitance increases observed as the synthesis temperature is decreased from 700 to 600 °C arise from the presence of smaller graphene-like domains^21,32^, although this cannot explain the large capacitance observed for the sample synthesised at 750 °C.

NMR measurements of electrolyte-soaked samples supported the findings from Raman Spectroscopy. The ^19^F MAS NMR spectra of the electrolyte-soaked carbons exhibit at least two resonances for the BF_4_^–^ anions (Fig. 1d), as in previous studies^21,24^, with the left-hand resonances assigned as “ex-pore” anions and the right-hand resonances assigned as “in-pore” anions. The Δ*δ* value is defined as the chemical shift difference between the in-pore and neat electrolyte resonances as in Eq. (1), and is dominated by the graphene-like domain sizes, with the carbon pore size having a minor impact on Δ*δ* for predominantly microporous carbons^22,24^. Notably, the Δ*δ* values observed here are much lower in magnitude than for conventional commercial activated carbons^21^, and Δ*δ* decreased in magnitude as the synthesis temperature was decreased from 700 to 600 °C (Fig. 1e). The carbons with higher gravimetric capacitances generally have Δ*δ* values of smaller magnitude (Fig. 1e and Table S5), consistent with our earlier findings^21^, with the exception of HTC-750 °C (see below). This trend still holds at a high current density of 1 A g^−1^ (Figure S6). This again suggests that the increasing capacitance at lower synthesis temperatures from 700 to 650 to 600 °C results from the decrease in the graphene-like domain sizes in nanoporous carbons.

As mentioned above, an abrupt increase of the gravimetric capacitance is noted for HTC-750 compared to the other carbons prepared at lower temperatures. We attribute this to the significant pore opening at 750 °C which gives rise to the sample with the highest BET surface area (2689 m^2^ g^−1^) in the series and a significant number of pores below 1 nm in diameter and around 2 nm in diameter (Fig. 1b and Table S1). This is likely due to the more dramatic etching of the carbon framework at 750 °C by KOH and the formation of potassium-containing compounds at this temperature (*i.e*. K_2_CO_3_ and K_2_O)^17,18^. Importantly, in addition to providing a measurement of the sizes of the graphene-like domains, our NMR experiments measure another key property of the nanoporous carbons; the electrolyte ion adsorption capacity. Indeed, the integrals of the in-pore resonances relate to the number of ions that can access the carbon nanopores in the absence of applied potential. Deconvolution of the NMR spectra of the electrolyte-saturated carbon samples shows that the gravimetric capacitance showed some correlation with the ion adsorption capacity (Fig. 1f), with HTC-750 having the largest ion adsorption capacity in the series (1.6 mmol g^−1^). The volumetric capacitance is also correlated with the ion adsorption capacity of the carbons in the absence of an applied potential (Figure S7), though for volumetric capacitance the correlation is less obvious (Figure S7). We hypothesise that the opening of the pores facilitates more pathways for ions to access the defective sites, where the charges are stored. However, an excessively high BET surface area is not always desirable, as it lowers the volumetric capacitance, though HTC-750 exhibits a similar volumetric capacitance to HTC-600 and HTC-650 (Fig. 1a and Figure S6). Moreover, comparing HTC-600 and HTC-750, the BET surface area normalised capacitance is around two times larger for HTC-600, than that of HTC-750 (Figure S6), highlighting the more efficient charge storage in HTC-600.

**Fig. 2:**
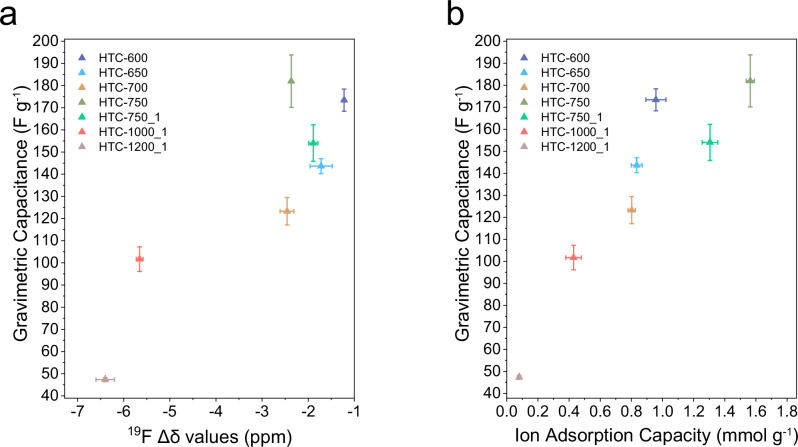
Structure-property correlation of the synthesised nanoporous carbons. **a** Correlation between gravimetric capacitance and ^19^F Δδ values for all the synthesised carbons, with the results of the additional series added. **b** Correlation between gravimetric capacitance and ion adsorption capacity for all the synthesised carbons.

An additional series of carbons was synthesised at 750 °C and at higher temperatures to explore the above effects further. As the carbons were synthesised under much higher temperatures, shorter carbonisation and activation times were used (1 h) to increase the yield. The carbons were labelled as “HTC_X_1” with “X” being the carbonisation and activation temperature and “1” representing the time (1 h). In general, improved capacitances were observed for carbons synthesised at lower temperatures (Fig. 2a, b). Among these, again HTC-750_1 showed the highest gravimetric capacitance, with a high BET surface area (2062 m^2^ g^−1^) and a large ion adsorption capacity (1.3 mmol g^−1^) (Fig. 2b), consistent with the more dramatic activation observed for HTC-750 above (Figure S3 and Table S1). However, similar to HTC-750, its volumetric and surface area normalised capacitances were lower than those of HTC-600 and HTC-650 (Figure S6), reaffirming that higher surface area does not always translate to better performance for commercial EDLCs. While there is some correlation between BET surface area and NMR-measured ion adsorption capacity for the HTC samples (Figure S7C), these quantities do not correlate well for commercial activated carbons. This suggests that BET surface area alone is insufficient to predict electrochemically accessible porosity, underscoring the value of the NMR approach over gas sorption-based methods. HTC-1000_1, with a Δ*δ* value of larger magnitude, exhibits a more ordered structure and lower capacitance (Fig. 2a), mimicking the behaviour of commercial activated carbons^10,21^. Finally, HTC-1200_1 shows a much lower capacitance, likely due to pore collapse at such high synthesis temperatures (Figure S3, Table S1), as indicated by the very small ion adsorption capacity of 0.08 mmol g^-1^ (Fig. 2b). Overall these results suggest that both the sizes of the graphene-like domains and ion adsorption capacities impact the capacitance, which is further explored below.

While our carbons with smaller graphene-like domains show enhanced capacitance per unit surface area, they do have some limitations that require further exploration and optimisation. Firstly, carbons synthesised at lower temperatures exhibit slightly poorer conductivity (Tables S6 and S7), as indicated by the more resistive voltammogram for HTC-600, which deviated more evidently from the ideal rectangular shape (Figure S1), and by the large initial semicircle in its EIS Nyquist plot (Figure S1), both reflecting its low electrical conductivity. Secondly, the low-temperature synthesised carbons were found to have reduced cycling stability compared to commercial activated carbons (Figure S2). Floating tests were conducted to study the cycle lives (See Methods) of the low-temperature synthesised carbons and showed more rapid degradation compared to commercial activated carbons (Figure S2). We hypothesise that the observed stability trade-offs may arise from two potential mechanisms^42^: (i) carbon edge sites acting as active catalytic sites that accelerate electrolyte decomposition reactions, or (ii) the disordered carbon structures themselves being inherently more reactive compared to more ordered carbons. While our low-temperature synthesised carbons show reduced cycling stability compared to commercial materials (*e.g*., HTC-750 degrades about 1.8 times faster than ACS-PC despite achieving 36% higher capacitance), this work serves as a fundamental proof-of-concept demonstrating that disorder engineering can achieve substantial performance gains. This work establishes disorder engineering as a viable design strategy, with the identified stability challenges providing clear targets for future electrode optimisation and electrolyte engineering efforts. The significance of cycling stability is application-dependent, with supercapacitor applications such as regenerative braking in electric vehicles, backup power systems, and pulse power delivery experiencing relatively infrequent cycling^43^ where enhanced energy density may outweigh stability concerns. Applications that require continuous cycling would benefit from the stability enhancement strategies identified for future development. The conductivity and degradation issues can potentially be addressed in the future by incorporating conductive additives, *e.g*. carbon black^44^, and by electrode mass balancing^45^, respectively, to improve the overall performance of the devices. We propose that the concept of low-temperature activated carbon synthesis can be extended beyond the specific methods used in this study *e.g*. by using a range of biomass precursors and activation methods.

### Impact of local structural order degree and ion adsorption capacity on capacitance

**Fig. 3:**
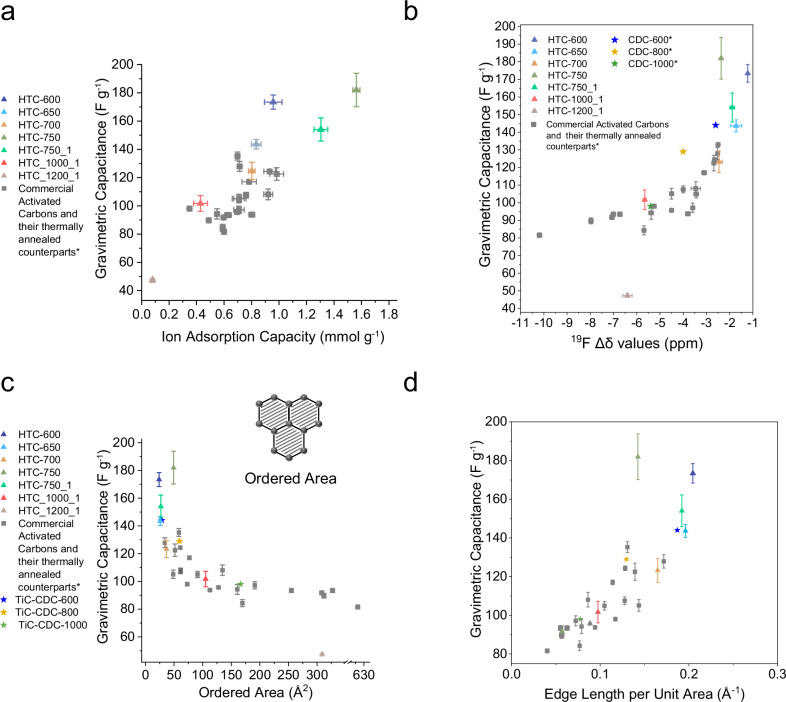
Impact of local structural order degree and ion adsorption capacity on capacitance. **a** Correlation between gravimetric capacitance and ion adsorption capacities, with commercial activated carbons, their thermally annealed counterparts data added from previous studies^3,21^. See Figure S7 for the relationship between ion adsorption capacities and BET surface areas. **b** Correlation between gravimetric capacitance and ^19^F Δ*δ* values, with commercial activated carbons, their thermally annealed counterparts and CDC data added from previous studies^3,21^. **c** Relationship between gravimetric capacitance and calculated ordered domain size of the synthesised nanoporous carbons, with commercial activated carbons, their thermally annealed counterparts and CDC data added from previous studies^21^. **d** Correlation between gravimetric capacitance and edge length per unit area for synthesised carbons, with commercial activated carbons and their thermally annealed counterparts data added from previous studies^21^. See Methods for detailed definitions of the edge length per unit area.

With an expanded series of nanoporous carbons including those from our previous studies, it is observed that carbons with higher gravimetric capacitances generally have (i) larger ion adsorption capacities (Fig. 3a) and (ii) Δ*δ* values of smaller magnitude (Fig. 3b), which extends our previous findings^21^. The correlation between the capacitance and the ion adsorption capacity was much less obvious in our previously studied commercial activated carbon series (Fig. 3a, grey points)^21^, likely because they have similar pore volumes (mostly 0.8–0.9 cc g^−1^) and ion adsorption capacities (0.6–0.8 mmol g^−1^), in which case the capacitive performance was more strongly dominated by the sizes of the graphene-like domains. However, with a wider range of nanoporous carbons including carbons with evidently large (HTC-750; 1.24 cc g^−1^ and HTC-750_1; 0.93 cc g^−1^) and small (HTC-1200_1; 0.32 cc g^−1^) pore volumes, a correlation is observed (Fig. 3a).

To extract the sizes of the graphene-like domains in the synthesised nanoporous carbons, we conducted NMR simulations based on our previously established method^24,30^, and as applied in previous studies^21^ (Fig. 3c). In general, the synthesised carbons follow the trend observed in our previous study that carbons with smaller calculated ordered domain sizes generally have higher capacitance^21^. Here our low-temperature synthesised carbons have both smaller graphene-like domains and larger capacitances compared to our previous commercial activated carbon series, extending the limits of our master plots (Fig. 3c). Among the carbons carbonised and activated for 6 h, the ordered domain sizes increased as the synthesis temperature increased, e.g. from 23.9 Å^2^ to 49.2 Å^2^ for HTC-600 and HTC-750, respectively. HTC-1000_1 exhibits similar behaviour to most commercial carbons, while HTC-1200_1 with an intermediate simulated ordered area of around 308 Å^2^ shows the lowest capacitance among all studied carbons, due to limited porosity and ion adsorption capacity (Figure S3 and Table S1).

Summarising, our analysis suggests that both the sizes of the graphene-like domains and the ion adsorption capacity are key factors that determine the capacitance of nanoporous carbons. Raman spectroscopy measurements show similar results with respect to disorder, with carbons having smaller *I*_D_/*I*_G_ values and larger D band full width half maxima (FWHM) generally exhibiting higher gravimetric capacitance (Figure S8), aligning with other previous observations^32^. While both techniques suggest that low temperature-synthesised carbons have smaller graphene-like domains, it is worth noting that Raman spectroscopy does not provide information on the ion adsorption capacity of the carbons, and therefore NMR provides a more complete picture.

Having established that both ion adsorption capacities and graphene-like domain sizes are important parameters in determining capacitance, we briefly speculate why this is the case here. First, the ion adsorption capacities indicate the accessibility of the ions to the sites where the charge is stored^37,38^, so it is logical that this correlates with capacitance. Second, to explore the role of the graphene-like domain size further, we plotted the capacitances of the nanoporous carbons against the “edge length per unit area” (Fig. 3d), which is a parameter defined to describe the approximate quantity of domain edges per unit area of graphene-like domains (see Methods). The value of edge length per unit area is larger for nanoporous carbons with smaller graphene-like domains (0.2 for HTC-600) and vice versa (0.097 for HTC-1000_1). Generally, a linear trend is observed, *i.e*. nanoporous carbons with larger edge length per unit area show larger capacitances (Fig. 3d). We hypothesise that edge sites accessible to electrolyte ions are where the charges are stored most efficiently^46^, leading to increased capacitance for nanoporous carbons with smaller graphene-like domains and larger edge length per unit area (Fig. 3d). Summarising, the ion adsorption capacity plays an important role together with the sizes of the graphene-like domains in determining the capacitance. We further hypothesise that while ion adsorption capacity and graphene-like domain size can be correlated (smaller domains providing more accessible edge sites), these parameters can also vary independently depending on synthesis conditions. For example, HTC-600 and HTC-650 exhibit similar ion adsorption capacities (0.95 and 0.8 mmol g^−1^) with comparable graphene-like domain sizes of 23.9 and 26 Å^2^, respectively. In contrast, both HTC_750 and HTC-750_1 show similar high ion adsorption (1.6 and 1.3 mmol g^−1^, respectively) through enhanced porosity but demonstrate different graphene-like domain sizes (49.2 and 27.1 Å^2^, respectively). These findings show that pore activation and domain size evolution can be independently controlled through synthesis temperature and conditions.

### Prediction of the capacitance from NMR spectra

**Fig. 4:**
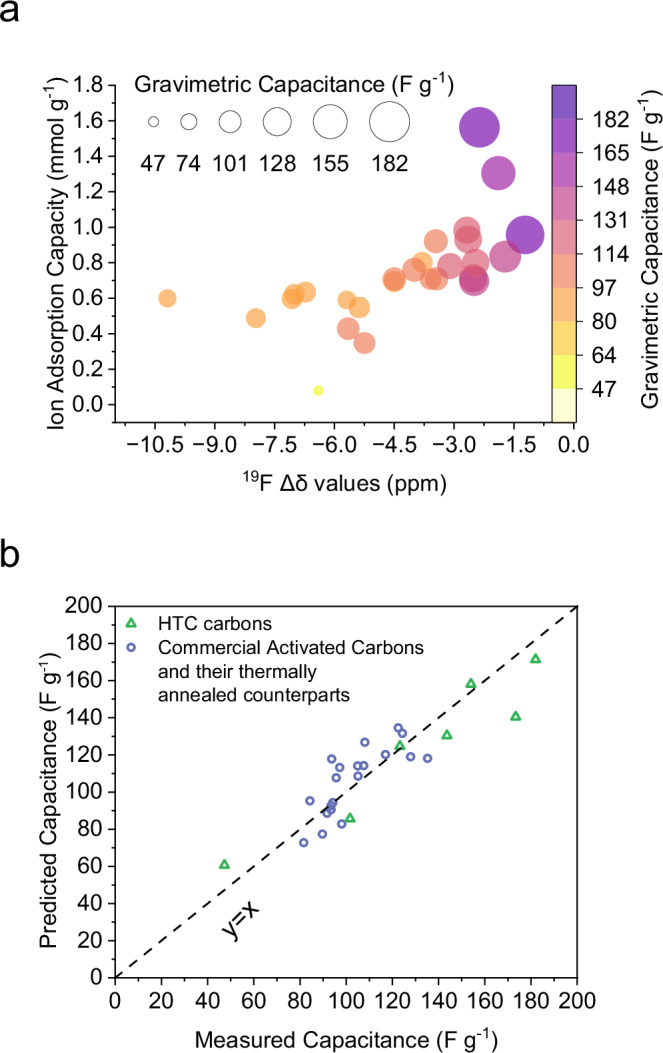
Prediction of the capacitance based on NMR measurements. **a** Relationship between gravimetric capacitances at 0.05 A g^−1^ and ion adsorption capacity and ^19^F Δ*δ* values for the synthesised carbons series, with commercial activated carbons and their thermally annealed counterparts data added from previous studies^21^. **b** Relationship between predicted capacitance and measured capacitance using Δ*δ* values and ion adsorption capacity measured from NMR experiments.

By combining the relationship between the Δ*δ* value, ion adsorption capacity, and gravimetric capacitance in a single graph (Fig. 4a), we again find that carbons with small Δ*δ* value and large ion adsorption capacities give rise to the largest capacitances (top right corner of Fig. 4a). A similar plot with ion adsorption capacities, graphene-like domain sizes and capacitances was also obtained (Figure S9). For carbons with similar graphene-like domain sizes (similar Δ*δ* value), the ion adsorption capacity becomes the dominant factor in determining capacitance. Conversely for carbons with similar ion adsorption capacities the sizes of the graphene-like domains (as measured by the Δ*δ* value) dominate (Fig. 4a). Figure 4a underscores the advantage of NMR spectroscopy, which simultaneously assesses both structural order and ion adsorption capacities with the electrolyte ions as the probe. Interestingly, carbons with smaller graphene-like domains (smaller Δ*δ* value) tend to exhibit larger ion adsorption capacities. We tentatively hypothesise that the graphene-like domain size might be inversely correlated with ion adsorption capacity, though more work is required to test this idea.

Mapping ion adsorption capacity, Δ*δ* values and gravimetric capacitance for a wide range of nanoporous carbons, we propose that the capacitance can be directly predicted from the ion adsorption capacity and the Δ*δ* value, both measurable in a single NMR experiment. (Fig. 4b). We used a linear regression approach combining Δ*δ* values and ion adsorption capacities measured from NMR spectroscopy to predict capacitance (see Methods). The best fit for the expression for the capacitance is:

2 $${Predicted}\,{capacitance}\,{of}\,{carbon}\,i={A}_{0}+{A}_{1}\times \varDelta \delta \left(i\right)+{A}_{2}\times {Q}_{{ion}}\left(i\right)$$

where *i* designates a specific carbon (*e.g*. HTC-600 or ACS-PC), Δ*δ*(i) and *Q*_ion_(i) are the Δ*δ* and ion adsorption capacity measured for this specific carbon, and *A*_*0*_, *A*_*1*_ and *A*_*2*_ are fitting parameters. A strong agreement was observed between the predicted and measured capacitances (Fig. 4b). Similar results were observed using the NMR-derived ordered area and ion adsorption capacity (Figure S9). It is worth noting that the Δ*δ* value can be directly extracted from NMR spectra whereas the NMR-derived ordered area requires further simulations, making it less accessible to a wider range of researchers. We emphasise that the carbons in this study are predominantly microporous conductive carbons with most pores below 2 nm (Fig. 1b and Figure S3). For non-porous carbons and/or meso/microporous carbons, the Δ*δ* values would be very small or negligible, and the Δ*δ* values would no longer correspond as straightforwardly to graphene-like domain sizes. Overall we find that NMR is a powerful tool to screen and predict the capacitances of predominantly microporous carbons.

## Discussion

In conclusion, our work demonstrates that low-temperature synthesis is a promising pathway to make nanoporous carbons with enhanced capacitance in EDLCs. Nanoporous carbons synthesised at low temperatures were found to have smaller graphene-like domains and improved gravimetric and volumetric capacitance compared with commercial activated carbons. Through a combination of NMR and Raman spectroscopy, we were able to provide a comprehensive understanding of the structural factors that influence capacitance. NMR spectroscopy, in particular, allowed us to simultaneously evaluate both the sizes of the graphene-like domains and the electrolyte ion adsorption capacities, with electrolyte ions as the probe. Our findings indicate that carbons with smaller graphene-like domains and higher ion adsorption capacity generally show enhanced capacitive performance. The highest gravimetric capacitances were achieved for either highly disordered carbons or carbons with more order but more accessible pores (higher electrolyte ion adsorption capacities), the latter carbons, however, generally being associated with lower volumetric capacitances. We show that the combination of Δ*δ* and the ion adsorption capacity both measured by NMR spectroscopy in a quick and simple NMR experiment ( ~ 5 mins) are excellent descriptors for capacitance, enabling rapid identification of high-performance materials without electrochemical testing. Importantly, this study provides clear design principles for creating high-performance nanoporous carbons for supercapacitors, which can be applied extensively across different synthesis routes and activation methods. NMR spectroscopy, as a dual probe for carbon disorder and electrolyte accessibility, proves to be a powerful tool to guide the design and synthesis of improved electrode materials for EDLCs.

## Methods

### Hydrothermal carbonisation

A 10 wt% D-glucose in water solution (20 mL) was prepared and stirred overnight. The prepared solution was then poured into quartz vials, and subsequently placed into 50 mL Teflon lined stainless steel autoclaves. The autoclaves were heated in a programmable oven to 230 °C at a heating rate of 5 °C min^−1^ and held for 12 h before cooling naturally cooled to room temperature. The HTC carbon was recovered by filtration, and washed with deionised water until a colourless aqueous phase was obtained. The HTC carbon was then dried at 80 °C under vacuum overnight.

### Chemical activation

The HTC carbon was chemically activated by physically mixing KOH pellets with the HTC carbon in a weight ratio of 3:1 using a mortar and pestle. The mixture was heated in a Carbolite Horizontal Tube Furnace GHA 12/300 at a rate of 5 °C min^−1^ under a 300 mL min^−1^ flow of N_2_ to the desired temperature for 1 or 6 h. The resulting activated carbon was washed sequentially with approximately 50 mL of 5 M HCl and 50 mL deionised water until the pH reached around 7, before drying at 110 °C in air overnight.

Free-standing carbon films were made by mixing carbon powder (90 wt%) and polytetrafluoroethylene binder (10 wt%) in ethanol. A measured amount of carbon powder (approximately 190 mg) was slowly poured into a watch glass with weighed PTFE and the mixture was stirred manually. After the ethanol had evaporated, the mixture was manually rolled into a film using a rolling pin with a thickness of 0.25 mm. The self-standing film was then placed into a folded aluminium foil and put into a vacuum oven at 100 °C for at least 24 h so that the residual ethanol and water could be fully removed.

### Gas physisorption analysis

Nitrogen sorption measurements were performed using a Micromeritics TriStar instrument at 77 K over a range of approximately 10^−5^ to 0.99 p/p_0_. 50–200 mg of samples were ground to a fine powder and degassed at 200 °C for 16 h under vacuum. The specific surface area was calculated by applying the Rouquerol correction to select an appropriate pressure range^47^.

The average pore sizes were calculated by taking the pore size value at half of the cumulative pore volume in the pore size distribution. Pore size distributions were calculated by 2D-non local density functional theory (NLDFT) based on a slit-pore model^40^.

### Coin cells and electrochemical measurement

All the coin cells were two-electrode cells (CR2032) and were assembled in a N_2_-filled glovebox (H_2_O < 0.1 ppm, O_2_ < 0.1 ppm). The coin cells were symmetric (i.e., both electrodes were the same carbon material). The electrodes were cut using a stainless-steel manual punching cutter (diameter 0.48 cm, Hilka Tools), and the two electrodes in each cell had identical masses within 0.2 mg. The masses of the electrodes ranged from 1.7 mg to 2.8 mg (Table S4).

Two electrodes were placed onto a coin cell bottom casing with a larger diameter (1.43 cm) glass fibre separator placed between the electrodes. Around 150 µL of 1 M tetraethylammonium tetrafluoroborate in acetonitrile (Sigma Aldrich) (1 M NEt_4_BF_4_/ACN) electrolyte was added around the separator with a Pasteur pipette. After the electrolyte fully wetted the electrodes, two SS316 spacer disks (0.5 mm thickness) were placed directly on top of the electrode, serving as current collectors. Additionally, a single SS316 spring was placed above the spacing disks. The top casing was then used to close the coin cell, with all coin cell parts made of stainless steel. The coin cells were sealed with a Compact Hydraulic Coin Cell Crimper (Cambridge Energy Solutions) under a pressure of 80 kg cm^−3^ for approximately 40 s. All coin cell parts were supplied by Cambridge Energy Solutions.

All electrochemical measurements were conducted in a two-electrode configuration with a Biologic BCS-805 potentiostat at room temperature. Cyclic voltammograms of the cells were first obtained at a scan rate of 10 mV s^-1^ with a fixed potential window of 0–2.5 V for at least 5 cycles. For constant charge-discharge measurements, each cell was measured sequentially under different current densities at 0.05, 0.1, 0.2, 0.5, 0.75, 1, 1.5 and 2 A g^−1^ with a voltage window of 0–2.5 V for at least 3 cycles under each current density. For each type of carbon, at least two cells were made. The capacitance of each cell was calculated through galvanostatic charge discharge measurements from the slope of the second half of the discharge curve in the last cycle. This method to calculate the capacitance of the single electrode assumes that the capacitance of two electrodes is equal. The capacitance was normalised by the active mass of carbon materials in electrodes. The error bars in the corresponding figures represent the standard deviations between the repetitive cells. Electrochemical impedance spectroscopy was conducted under a frequency range from 0.01 to 200k Hz. See our previous work for more details on calculations of the gravimetric and volumetric capacitance^21^.

For floating tests, after every 6 h of aging with a constant voltage hold at 2.7 V, a constant current charge-discharge measurement at 0.5 A g^−1^ with a voltage window of 0–2.7 V was conducted to derive the capacitance. These sequences were reiterated 5 times giving a total floating time of 30 h.

### Solid-state NMR spectroscopy experiments

Each carbon sample was made into a carbon film as described above and dried for at least 24 h at 100 °C in vacuo before being transferred to a N_2_-filled glovebox. The film was then cut and weighed in the glovebox. The weighed film piece (around 5 mg) was put into a sealed vial overnight with 1 M NEt_4_BF_4_ (ACN) to fully saturate the sample (around 150 µL) before being packed into 2.5 mm rotors. The vial was sealed with PTFE tape on the vial thread and a layer of electrical tape on the outside. The rotors were quickly packed (typically within 3 min) to avoid evaporation of the acetonitrile solvent. For each sample, the rotor was weighed before and after being packed to calculate the mass of the electrolyte added to the system. The excess electrolyte was carefully removed with tissue paper after packing.

NMR spectroscopy experiments were carried out with a Bruker Avance Neo spectrometer in a Bruker 2.5 mm HX double resonance probe. Measurements were carried out at a magnetic field strength of 9.4 T, corresponding to a ^1^H Larmor frequency of 400.1 MHz. All spectra were acquired with a 90° pulse-acquire sequence at a sample spinning speed of 5 kHz. Recycle delays were set to be more than five times T_1_ for each sample to ensure that the experiments were quantitative. The recycle delays are typically 8-12 s. ^19^F NMR spectra were referenced relative to neat hexafluorobenzene (C_6_F_6_) at −164.9 ppm as a secondary reference. See our previous paper for details on deconvolution of the NMR spectra^21^.

^13^C NMR spectroscopy experiments were carried out with a Bruker Avance Neo spectrometer in a Bruker 3.2 mm HXY triple resonance probe. Measurements were carried out at a magnetic field strength of 14 T, corresponding to a ^1^H Larmor frequency of 600.1 MHz. All spectra were acquired with a spin-echo pulse sequence (90° − τ − 180° − τ−acquire) at a sample spinning speed of 20 kHz. A spin-echo delay *τ* of 50 µs (a single rotor period) was used. Recycle delays were set to be 10 s. ^13^C NMR spectra were referenced relative to adamantane at 29.5 ppm as a secondary reference

### X-ray photoelectron spectroscopy

X-ray photoelectron spectroscopy (XPS) analysis was performed using a Thermo Fisher K-Alpha XPS system, and the acquired spectra were analysed using the Avantage software.

### Raman spectroscopy

Raman spectra were collected on a Renishaw inVia micro-Raman (1000–3000 cm^−1^), using a 50 mW 532 nm laser at 10% laser power.

All Raman spectra were normalized by maximum intensity, baseline-subtracted and deconvoluted using a four-peak model with Lorentzian functions. Fit parameters included peak positions, full widths at half maximum (FWHM), and peak areas. Each spectrum was deconvoluted by two different researchers from Imperial College London and Cambridge. For each fitting, different initial peak positions were applied, and then varied freely to achieve the optimal fitting. The results in Fig. 1 and Figure S8 represent the average of the four fits (two fits from each researcher), with the error bars showing the standard deviation among the different fits. For four-peak analysis with constraints, the position of the left hand “shoulder” peak was kept consistent between 1180 and 1200 cm^−1^, and the position of the right hand “shoulder” was maintained at 1500 to 1530 cm^−1^ for all spectra. The full widths at half maximum (FWHM) and peak areas were varied freely to achieve the optimal fitting.

### Calculation of the ordered areas

The ordered areas of the carbons, used as a characterization of carbon domain sizes, were calculated through a comparison between measured and simulated Δ*δ* values, as described in our previous work^21^. In particular, the Δ*δ* values were simulated using a mesoscopic model which describes the diffusion of fluid molecules in porous carbons and predicts the NMR spectra of those species^30^. The parameters of the model (electrolyte considered, lattice size, aromatic molecules used to estimate the nucleus independent chemical shifts, etc.…) are all the same as in our previous work except for the pore size distributions which are the ones corresponding to the carbons synthesized in this work (see Figs. 1b and S3b, S3d)^21^.

### Prediction of the capacitances

In this work, we try to predict the capacitance using a linear regression with multiple variables. The best fit for the expression for the capacitance is:

3 $${Predicted}\,{capacitance}\,{of}\,{carbon}\,i={A}_{0}+{A}_{1}\times \varDelta \delta \left(i\right)+{A}_{2}\times {Q}_{{ion}}\left(i\right)$$

where *i* designates a specific carbon (*e.g*. HTC-600 or ACS-PC), Δ*δ*(i) and *Q*_ion_(i) are the Δ*δ* and ion adsorption capacity measured for this specific carbon, and *A*_*0*_, *A*_*1*_ and *A*_*2*_ are fitting parameters.

The determination of the fitting parameters is done by calculating the closed-form solution to the linear regression:

4 $$A={\left({X}^{T}X\right)}^{-1}{X}^{T}\vec{y}$$

Where *A* is the vector containing the fitting parameters,

5 $$A=\left(\begin{array}{c}{A}_{0}\\ {A}_{1}\\ {A}_{2}\end{array}\right)$$

*X* is the matrix containing the measured values of Δ*δ* and *Q*_ion_ for all 27 carbons considered,

6 $$X=\,\left(\begin{array}{ccc}1 & \varDelta \delta \left(1\right) & {Q}_{{ion}}\left(1\right)\\ \vdots & \vdots & \vdots \\ 1 & \varDelta \left(27\right) & {Q}_{{ion}}\left(27\right)\end{array}\right)$$

and $\vec{y}$ is the vector containing the measured capacitances.

The parameters obtained when training on Δ*δ* values in ppm, *Q*_ion_ in mmol g^−1^ and capacitances in F g^−1^ are *A*_0_ = 88.62, *A*_1_ = 5.13 and *A*_2_ = 60.70.

The linear regression can also be done on ordered areas (in *Å*^2^) instead of Δ*δ* values. The parameters are then *A*_0_ = 68.77, *A*_1_ = −0.068 and *A*_2_ = 70.44.

The agreement between predicted and measured capacitances seems to be similar in both cases (Fig. 4b and Figure S9b).

It is worth noting that the actual values of the coefficients are not especially relevant since the values are in different units and span different ranges.

Additional tests were done by training the parameters on a random selection of half of the carbons and testing them on the remaining half. This gives relatively good results suggesting that actual prediction for new carbons not included in the current study could work well.

### Edge length per unit area

The sizes of the graphene-like domains were estimated as squares. Therefore, the number of domains per unit area is defined as,

7 $${Number}\,{of}\,{domains}\,{per}\,{unit}\,{area}=\frac{1}{A}$$

where 1 (*Å*^2^) is the unit area, and A is the NMR-derived ordered area (*Å*^2^).

The edge length per unit area is calculated as,

8 $${Edge}\,{length}\,{per}\,{unit}\,{area}={Number}\,{of}\,{domains}\,{per}\,{unit}\,{area}*{domain}\,{perimeter}$$

where the domain perimeter is $4\sqrt{A}$ as the sizes of the domains were estimated to be squares.

Therefore, the edge length per unit area is proportional to

9 $${Edge}\,{length}\,{per}\,{unit}\,{area}\propto \frac{1}{\sqrt{A}}$$

If the sizes of the graphene-like domains were treated as regular hexagons. The number of domains remained unchanged according to Eq. (6). The domain perimeter is $6\sqrt{\frac{2A}{3\sqrt{3}}}$ for regular hexagons.

As a result, the relationship in (8) holds.

### CHN combustion measurement

C, H and N concentrations (wt%) were determined through CHN combustion analysis using an Exeter Analytical CE-440, with combustion at 975 °C. All samples were put into a vacuum oven at 100 °C for at least 12 h before the tests. Each sample was measured twice.

### 4-point probe conductivity measurement

The electrical conductivity was measured using a Jandel cylindrical four-point probe head with 1 mm needle spacing and 300 µm needle diameter. The apparatus was connected with a Keysight 34410 A digital multimeter to interpret the measured resistance.

## Supplementary information

Supplementary Information

Transparent Peer Review file

## Data Availability

All data are available in the main text or the supplementary materials. All raw experimental data files are available in the Cambridge Research Repository, Apollo. 10.17863/CAM.116043

## References

[1] Simon, P. & Gogotsi, Y. Perspectives for electrochemical capacitors and related devices. *Nat. Mater.* **19**, 1151–1163 (2020). 32747700 10.1038/s41563-020-0747-z

[2] Zhang, L. L. & Zhao, X. S. Carbon-based materials as supercapacitor electrodes. *Chem. Soc. Rev.* **38**, 2520–2531 (2009). 19690733 10.1039/b813846j

[3] Chmiola, J. et al. Anomalous increase in carbon capacitance at pore sizes less than 1 nanometer. *Science* **313**, 1760–1763 (2006). 16917025 10.1126/science.1132195

[4] de Tomas, C. et al. Structural prediction of graphitization and porosity in carbide-derived carbons. *Carbon* **119**, 1–9 (2017).

[5] Jain, S. K., Pellenq, R. J. M., Pikunic, J. P. & Gubbins, K. E. Molecular modeling of porous carbons using the hybrid reverse monte carlo method. *Langmuir* **22**, 9942–9948 (2006). 17106983 10.1021/la053402z

[6] de Tomas, C., Suarez-Martinez, I. & Marks, N. A. Carbide-derived carbons for dense and tunable 3D graphene networks. *Appl. Phys. Lett.* **112**, 10.1063/1.5030136 (2018).

[7] Palmer, J. C., Brennan, J. K., Hurley, M. M., Balboa, A. & Gubbins, K. E. Detailed structural models for activated carbons from molecular simulation. *Carbon* **47**, 2904–2913 (2009).

[8] Yushin, G., Dash, R., Jagiello, J., Fischer, J. E. & Gogotsi, Y. Carbide-derived carbons: effect of pore size on hydrogen uptake and heat of adsorption. *Adv. Funct. Mater.* **16**, 2288–2293 (2006).

[9] Borchardt, L., Oschatz, M. & Kaskel, S. Tailoring porosity in carbon materials for supercapacitor applications. *Mater. Horiz.* **1**, 157–168 (2014).

[10] Lyu, D. X. et al. Understanding sorption of aqueous electrolytes in porous carbon by NMR spectroscopy. *J. Am. Chem. Soc.* **146**, 9897–9910 (2024). 38560816 10.1021/jacs.3c14807 PMC11009947

[11] Oda, H., Yamashita, A., Minoura, S., Okamoto, M. & Morimoto, T. Modification of the oxygen-containing functional group on activated carbon fiber in electrodes of an electric double-layer capacitor. *J. Power Sources* **158**, 1510–1516 (2006).

[12] Rose, M. et al. Hierarchical micro- and mesoporous carbide-derived carbon as a high-performance electrode material in supercapacitors. *Small* **7**, 1108–1117 (2011). 21449047 10.1002/smll.201001898

[13] Chmiola, J., Yushin, G., Dash, R. & Gogotsi, Y. Effect of pore size and surface area of carbide derived carbons on specific capacitance. *J. Power Sources* **158**, 765–772 (2006).

[14] Yin, H., Shao, H., Daffos, B., Taberna, P.-L. & Simon, P. The effects of local graphitization on the charging mechanisms of microporous carbon supercapacitor electrodes. *Electrochem. Commun.* **137**, 107258 (2022).

[15] Rodríguez-Reinoso, F. & Molina-Sabio, M. Activated carbons from lignocellulosic materials by chemical and/or physical activation: an overview. *Carbon* **30**, 1111–1118 (1992).

[16] Gao, Y., Yue, Q., Gao, B. & Li, A. Insight into activated carbon from different kinds of chemical activating agents: a review. *Sci. Total Environ.* **746**, 141094 (2020). 32745853 10.1016/j.scitotenv.2020.141094

[17] Wang, J. & Kaskel, S. KOH activation of carbon-based materials for energy storage. *J. Mater. Chem.* **22**, 23710–23725 (2012).

[18] Wei, L., Sevilla, M., Fuertes, A. B., Mokaya, R. & Yushin, G. Hydrothermal carbonization of abundant renewable natural organic chemicals for high-performance supercapacitor electrodes. *Adv. Energy Mater.* **1**, 356–361 (2011).

[19] Hu, B. et al. Engineering carbon materials from the hydrothermal carbonization process of biomass. *Adv. Mater.* **22**, 813–828 (2010). 20217791 10.1002/adma.200902812

[20] Lozano-Castelló, D., Calo, J. M., Cazorla-Amorós, D. & Linares-Solano, A. Carbon activation with KOH as explored by temperature programmed techniques, and the effects of hydrogen. *Carbon* **45**, 2529–2536 (2007).

[21] Liu, X. Y. et al. Structural disorder determines capacitance in nanoporous carbons. *Science* **384**, 321–325 (2024). 38635707 10.1126/science.adn6242

[22] Forse, A. C., Griffin, J. M., Presser, V., Gogotsi, Y. & Grey, C. P. Ring current effects: factors affecting the NMR chemical shift of molecules adsorbed on porous carbons. *J. Phys. Chem. C* **118**, 7508–7514 (2014).

[23] Deschamps, M. et al. Exploring electrolyte organization in supercapacitor electrodes with solid-state NMR. *Nat. Mater.* **12**, 351–358 (2013). 23416727 10.1038/nmat3567

[24] Forse, A. C. et al. New insights into the structure of nanoporous carbons from nmr, raman, and pair distribution function analysis. *Chem. Mater.* **27**, 6848–6857 (2015).

[25] Borchardt, L., Oschatz, M., Paasch, S., Kaskel, S. & Brunner, E. Interaction of electrolyte molecules with carbon materials of well-defined porosity: characterization by solid-state NMR spectroscopy. *Phys. Chem. Chem. Phys.* **15**, 15177–15184 (2013). 23925570 10.1039/c3cp52283k

[26] Fulik, N. et al. Electrolyte mobility in supercapacitor electrodes - solid state NMR studies on hierarchical and narrow pore sized carbons. *Energy Storage Mater.* **12**, 183–190 (2018).

[27] Bragg, R. J. et al. Solvation effects on aqueous ion adsorption and electrosorption in carbon micropores. *Carbon* **229**, 119531 (2024).

[28] Harris, R. K., Thompson, T. V., Norman, P. R. & Pottage, C. Phosphorus-31 NMR studies of adsorption onto activated carbon. *Carbon* **37**, 1425–1430 (1999).

[29] Haigh, C. W. & Mallion, R. B. Ring current theories in nuclear magnetic resonance. *Prog. Nucl. Magn. Reson. Spectrosc.* **13**, 303–344 (1979).

[30] Merlet, C., Forse, A. C., Griffin, J. M., Frenkel, D. & Grey, C. P. Lattice simulation method to model diffusion and NMR spectra in porous materials. *J. Chem. Phys.* **142**, 094701 (2015). 25747093 10.1063/1.4913368

[31] Ferrari, A. C. & Robertson, J. Interpretation of Raman spectra of disordered and amorphous carbon. *Phys. Rev. B* **61**, 14095–14107 (2000).

[32] Liu, X. Y. et al. Raman spectroscopy measurements support disorder-driven capacitance in nanoporous carbons. *J. Am. Chem. Soc.* **146**, 30748–30752 (2024). 39486400 10.1021/jacs.4c10214 PMC11565708

[33] Lespade, P., Aljishi, R. & Dresselhaus, M. S. Model for Raman-scattering from incompletely graphitized carbons. *Carbon* **20**, 427–431 (1982).

[34] Ferrari, A. C. & Basko, D. M. Raman spectroscopy as a versatile tool for studying the properties of graphene. *Nat. Nanotechnol.* **8**, 235–246 (2013). 23552117 10.1038/nnano.2013.46

[35] Guo, J. et al. Topological defects: origin of nanopores and enhanced adsorption performance in nanoporous carbon. *Small* **8**, 3283–3288 (2012). 22893594 10.1002/smll.201200894

[36] Osswald, S., Chmiola, J. & Gogotsi, Y. Structural evolution of carbide-derived carbons upon vacuum annealing. *Carbon* **50**, 4880–4886 (2012).

[37] Lian, C. et al. Enhancing the capacitive performance of electric double-layer capacitors with ionic liquid mixtures. *Acs Energy Lett.* **1**, 21–26 (2016).

[38] Segalini, J., Iwama, E., Taberna, P.-L., Gogotsi, Y. & Simon, P. Steric effects in adsorption of ions from mixed electrolytes into microporous carbon. *Electrochem. Commun.* **15**, 63–65 (2012).

[39] Li, X. -r et al. Effect of the oxygen functional groups of activated carbon on its electrochemical performance for supercapacitors. *N. Carbon Mater.* **35**, 232–243 (2020).

[40] Jagiello, J. & Olivier, J. P. 2D-NLDFT adsorption models for carbon slit-shaped pores with surface energetical heterogeneity and geometrical corrugation. *Carbon* **55**, 70–80 (2013).

[41] Cançado, L. G. et al. Quantifying defects in graphene via Raman spectroscopy at different excitation energies. *Nano Lett.* **11**, 3190–3196 (2011). 21696186 10.1021/nl201432g

[42] Pameté, E. et al. The many deaths of supercapacitors: degradation, aging, and performance fading. *Adv. Energy Mater.* **13**, 2301008 (2023).

[43] Rugolo, J. & Aziz, M. J. Electricity storage for intermittent renewable sources. *Energy Environ. Sci.* **5**, 7151–7160 (2012).

[44] Jäckel, N. et al. Comparison of carbon onions and carbon blacks as conductive additives for carbon supercapacitors in organic electrolytes. *J. Power Sources* **272**, 1122–1133 (2014).

[45] Jing, L. et al. The mass-balancing between positive and negative electrodes for optimizing energy density of supercapacitors. *J. Am. Chem. Soc.* **146**, 14369–14385 (2024). 38718351 10.1021/jacs.4c00486

[46] Banerjee, S. et al. Electrochemistry at the edge of a single graphene layer in a nanopore. *Acs Nano* **7**, 834–843 (2013). 23249127 10.1021/nn305400n PMC3551991

[47] Rouquerol, F., Rouquerol, J. & Sing, K. CHAPTER 6 - Assessment of surface area. in *Adsorption by Powders and Porous Solids*, 165–189 (Academic Press, 1999).

