# Supplementary material for: Highly disordered nanoporous carbons for enhanced energy storage in supercapacitors
Source: Nat Commun. 2026 Apr 20;17:5464. doi: 10.1038/s41467-026-71520-x (PMC13284229; doi:10.1038/s41467-026-71520-x)
Supplement: Supplementary file 1 — Supplementary Information [file 41467_2026_71520_MOESM1_ESM.pdf]

Supporting Information: Highly Disordered Nanoporous Carbons for Enhanced Energy Storage in Supercapacitors

Xinyu Liu,[1+] Robert D. Hunter,[2+,] Zhen Xu,[1] El Hassane Lahrar,[3,4] Céline Merlet,[4,5] Clare P. Grey,[1*] Maria-Magdalena Titirici,[2] Alexander C. Forse[1*]

+ Authors contributed equally

Corresponding Authors

Clare P. Grey and Alexander C. Forse

*cpg27@cam.ac.uk

*acf50@cam.ac.uk

1 Yusuf Hamied Department of Chemistry, University of Cambridge, Cambridge CB2 1EW, U.K.

2 Department of Chemical Engineering, Imperial College London, London, SW7 2AZ, U.K.

3 Sorbonne Université, CNRS, Physicochimie des Électrolytes et Nanosystèmes Interfaciaux, F-75005 Paris, France.

4 Réseau sur le Stockage Électrochimique de l'Énergie (RS2E), Fédération de Recherche CNRS 3459, Amiens 80039, France

5 CIRIMAT, Université de Toulouse, Toulouse INP, CNRS, 118 Route de Narbonne, 31062 Toulouse cedex 9, France

Contents

Supplementary Figures 3

Supplementary Tables 18

Supplementary References 25

Supplementary Figures

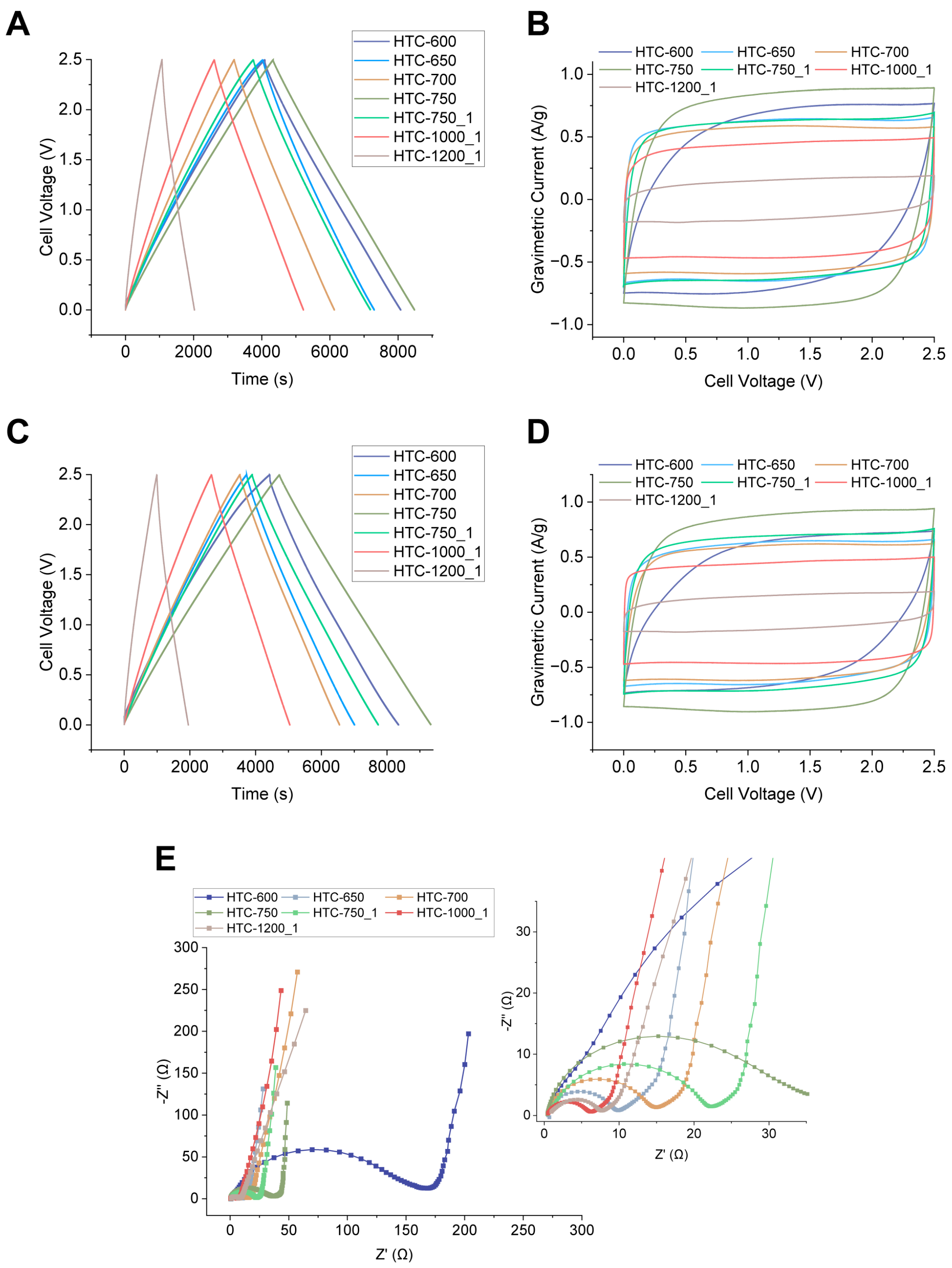

Figure S1: Galvanostatic charge–discharge plots and cyclic voltammograms of the synthesised carbons, with repeat measurements shown, and EIS plots of the synthesised carbons. (**A**) Galvanostatic charge–discharge plots of the synthesised nanoporous carbons in 1 M NEt_4BF_4 /ACN at 0.05 A g^{-1}. (**B**) Cyclic voltammograms of the synthesised nanoporous

carbons at 10 mV s^{-1}. (**C**) Galvanostatic charge−discharge plots of the repeat cells of synthesised nanoporous carbons in 1 M NEt_4BF_4 /ACN at 0.05 A g^{-1}. (**D**) Cyclic voltammograms of the repeat cells of synthesised nanoporous carbons at 10 mV s^{-1}. (**E**) EIS measurements of the cells of synthesised nanoporous carbons in 1 M NEt_4BF_4 /ACN using the frequency ranging from 0.01 to 200k Hz.

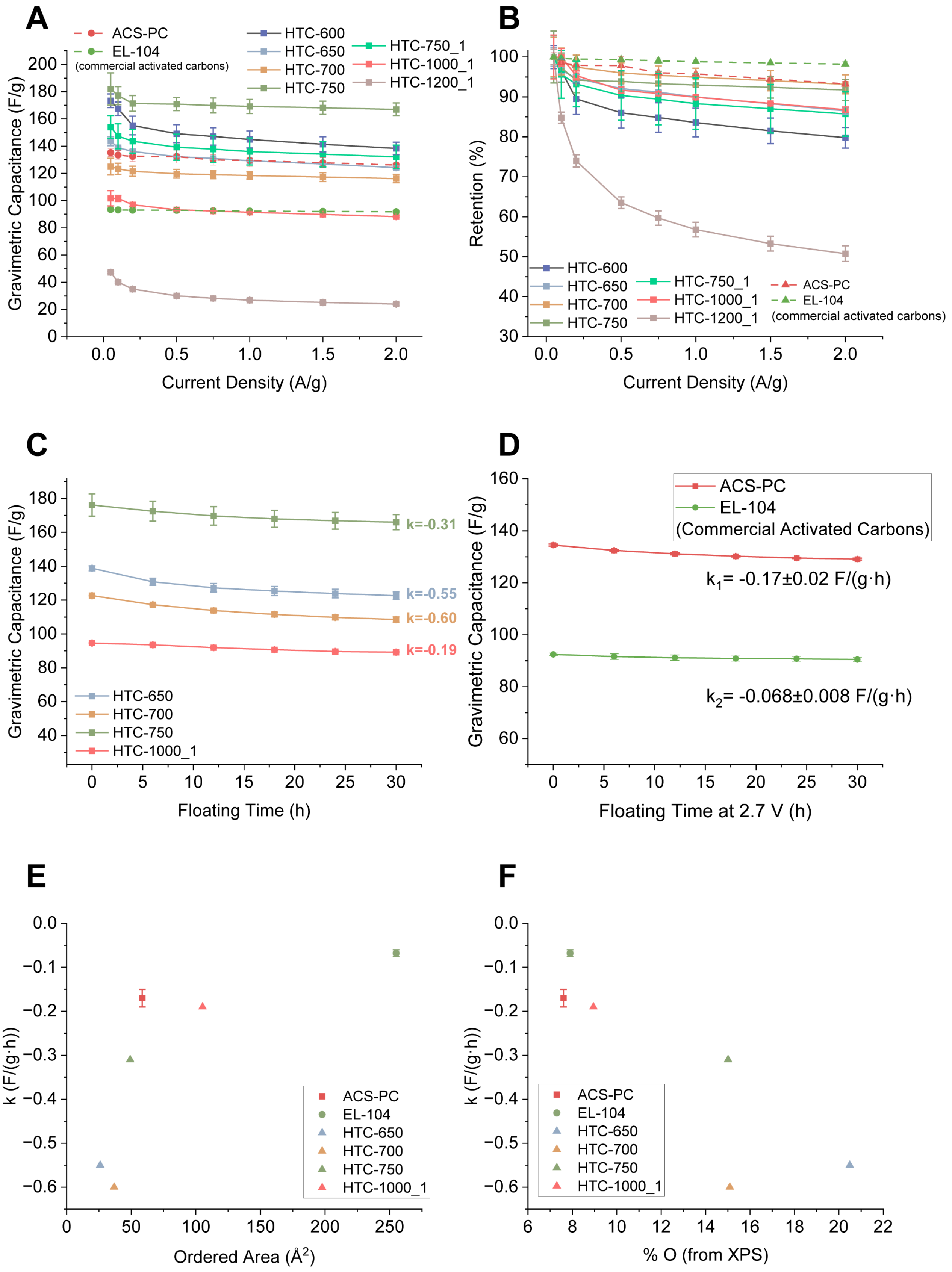
A
ACS-PC
EL-104
(commercial activated carbons)
HTC-600
HTC-650
HTC-700
HTC-750
HTC-750_1
HTC-1000_1
HTC-1200_1
Gravimetric Capacitance (F/g)
Current Density (A/g)
B
Retention (%)
Current Density (A/g)
C
k=-0.31
k=-0.55
k=-0.60
k=-0.19
Gravimetric Capacitance (F/g)
Floating Time (h)
D
(Commercial Activated Carbons)
k1= -0.17±0.02 F/(g·h)
k2= -0.068±0.008 F/(g·h)
Gravimetric Capacitance (F/g)
Floating Time at 2.7 V (h)
E
k (F/(g·h))
Ordered Area (Å2)
F
% O (from XPS)

Figure S2: Gravimetric capacitances at different current densities and cyclability of the synthesised carbons. (**A**) Gravimetric capacitances of the synthesised and two commercial nanoporous carbons in 1 M NEt_4BF_4 /ACN at different current densities. (**B**) Capacitance retention ratio of the synthesised and two commercial nanoporous carbons and in 1 M NEt_4BF_4 /ACN at different current densities, with the retention ratio calculated by gravimetric capacitance at *x* current density divided by the gravimetric capacitance at 0.05 A g^{-1}. (**C**) Gravimetric capacitances of selected synthesised carbons as a function of floating time at a cell voltage of 2.7 V. *k* is the slope of the linear curve, representing the capacitance degradation rate (units: F $g^{-1}h^{-1}$). (**D**) Gravimetric capacitances of two commercial activated carbons (ACS-PC; more disordered and EL-104; more ordered) as a function of floating time at a cell voltage of 2.7 V, reproduced from our previous work.[2]It is observed that the synthesised carbons have worse stability than commercial activated carbons, which should be optimised in future studies, *e.g.* through electrode mass balancing. (**E**) Relationship between capacitance degradation rate (units: F $g^{-1}h^{-1}$) and ordered area. (F) Relationship between capacitance degradation rate and %O from XPS measurements. In general, synthesised nanoporous carbons with higher %O content degraded faster than commercial activated carbons with lower %O content, suggesting that high oxygen content in synthesised carbons together with structural disorder might contribute to instability of the carbons.

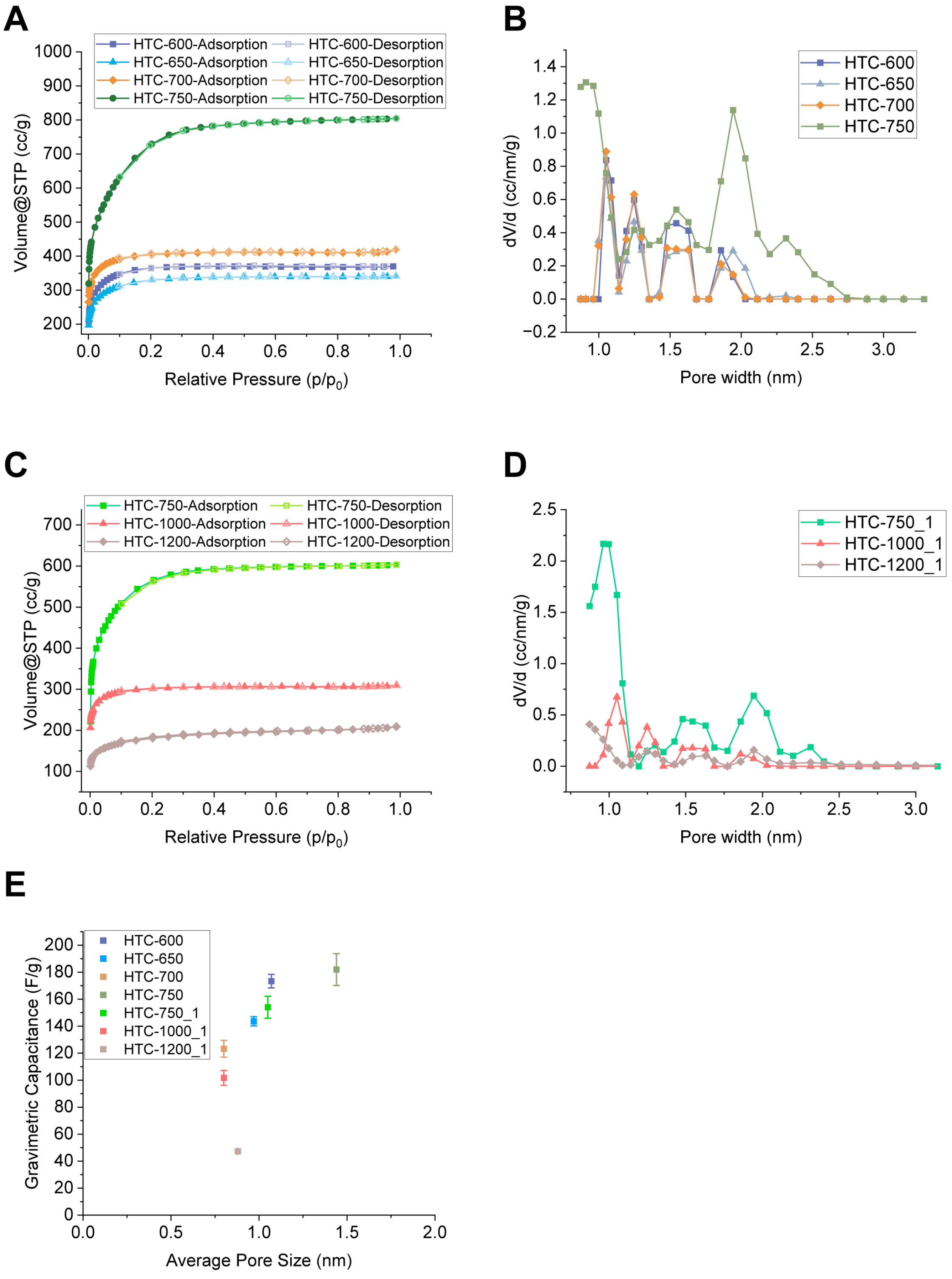

Figure S3: Gas sorption isotherms and pore size distribution plots of the synthesised carbons. (**A**) N_2 isotherms at 77 K of the four synthesised nanoporous carbons (filled dots show adsorption, hollow dots show desorption). **(B)** Pore size distributions based on 2D-NLDFT analysis (slit pore model) of N_2 isotherms at 77 K in **(A)**.[1] (**C**) N_2 isotherms at 77 K of

three synthesised nanoporous carbons (filled dots show adsorption, hollow dots show desorption). **(D)** Pore size distributions based on 2D-NLDFT analysis (slit pore model) of N_2 isotherms at 77 K in **(C)**.[1] **(E)** Relationship between gravimetric capacitances of the synthesised nanoporous carbons in 1 M NEt_4BF_4 /ACN and average pore size.

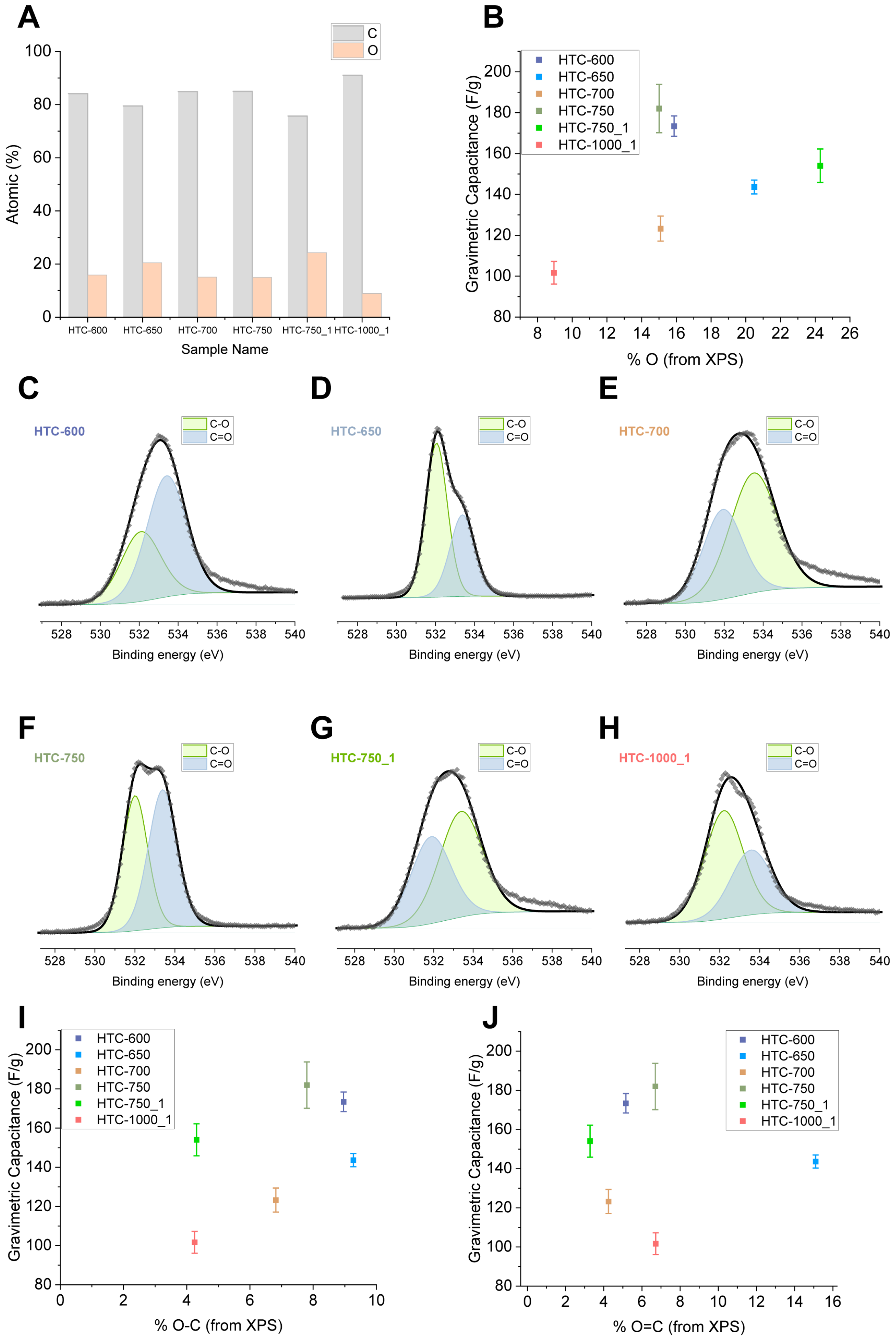

Figure S4: X-ray photoelectron spectroscopy (XPS) results of the synthesised carbons. (A) XPS survey of the synthesised nanoporous carbons. **(B)** Relationship between gravimetric

capacitances in 1 M NEt_4BF_4 /ACN and %O (atomic percent) measured from XPS for the synthesised carbons, with no clear correlation observed, similar to our previous study.[2] Fitted O1s core-level spectra of **(C)** HTC-600, **(D)** HTC-650, **(E)** HTC-700, **(F)** HTC-750, **(G)** HTC-750_1 and **(H)** HTC-1000_1. **(I)** Relationship between gravimetric capacitances in 1 M NEt_4BF_4 /ACN and %O-C (atomic percent) type functional groups measured from XPS for the synthesised carbons. **(J)** Relationship between gravimetric capacitances in 1 M NEt_4BF_4 /ACN and %O=C (atomic percent) type functional groups measured from XPS for the synthesised carbons.

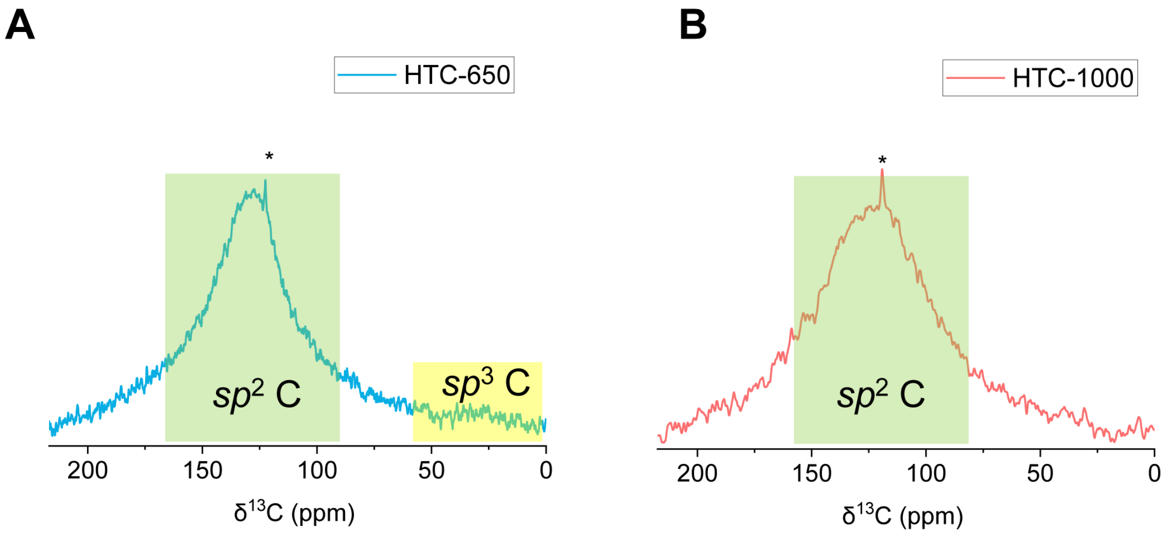

Figure S5: ^{13}C MAS NMR spectra (20 kHz, 14 T) of the synthesised carbons. (A) HTC-650. **(B)** HTC-1000 "*" represents minor amount of C=C impurities in the synthesised carbons. It is unclear whether trace amount of sp^3 hybridised carbons is present within the structure.

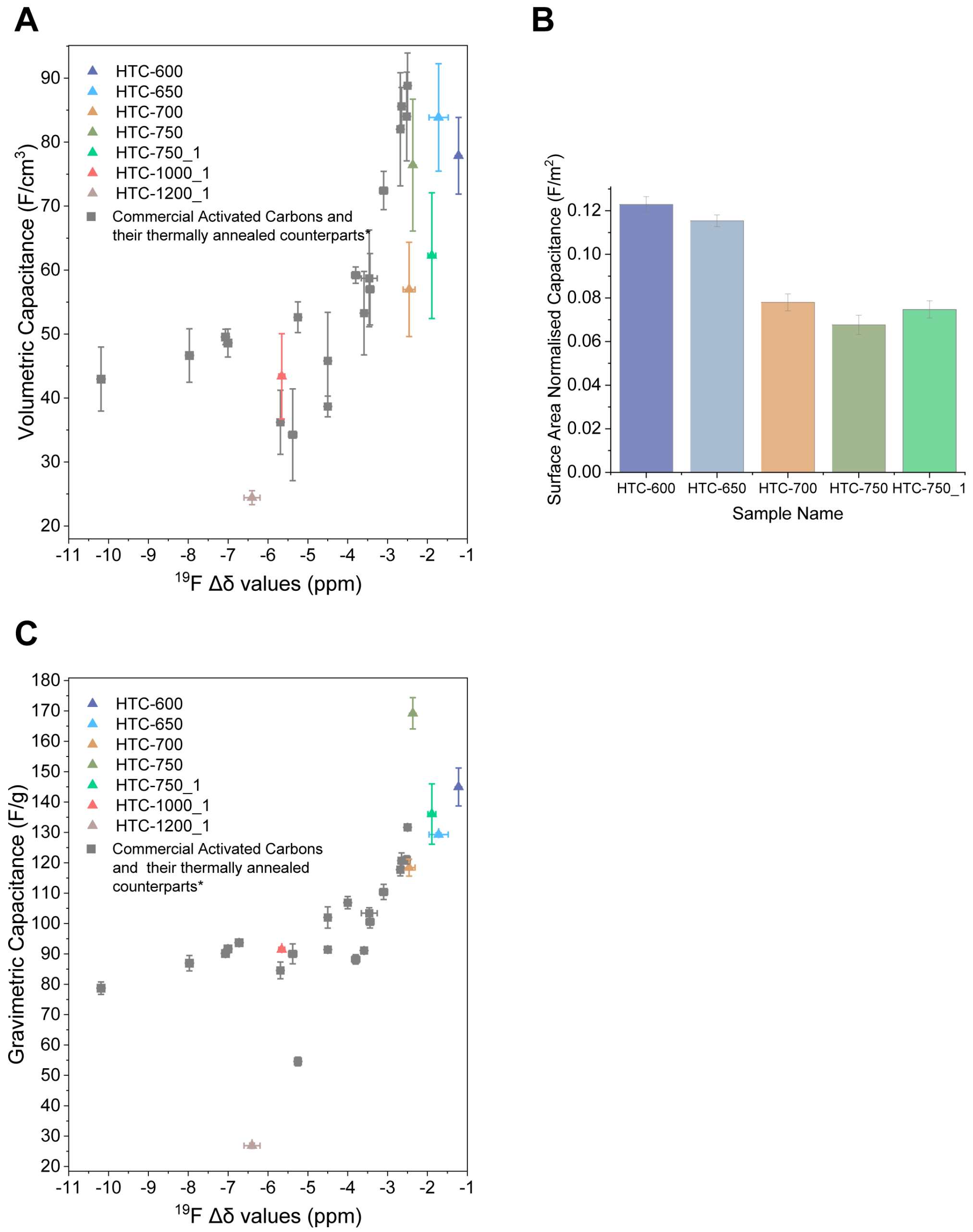

Figure S6: Correlation between Δδ values and gravimetric and volumetric capacitance at different current densities. **(A)** Correlation between volumetric capacitance and ^{19}F Δδ values of the synthesised carbons in 1 M NEt_4BF_4 /ACN at 0.05 A g^{-1}, with data on commercial activated carbons and their thermally annealed counterparts added from previous studies.[2] (**B**) BET surface area normalised capacitance of HTC-600, HTC-650, HTC-700, HTC-750 and HTC-750_1. Although HTC-750 and HTC-750_1 show high gravimetric capacitances, the volumetric capacitances and surface area normalised capacitances are lower than those of HTC-600 and HTC-650. **(C)** Correlation between gravimetric capacitance and ^{19}F Δδ values of the synthesised carbons in 1 M NEt_4BF_4 /ACN at 1 A g^{-1}, with commercial activated carbons and their thermally annealed counterparts data added from our previous study.[2]

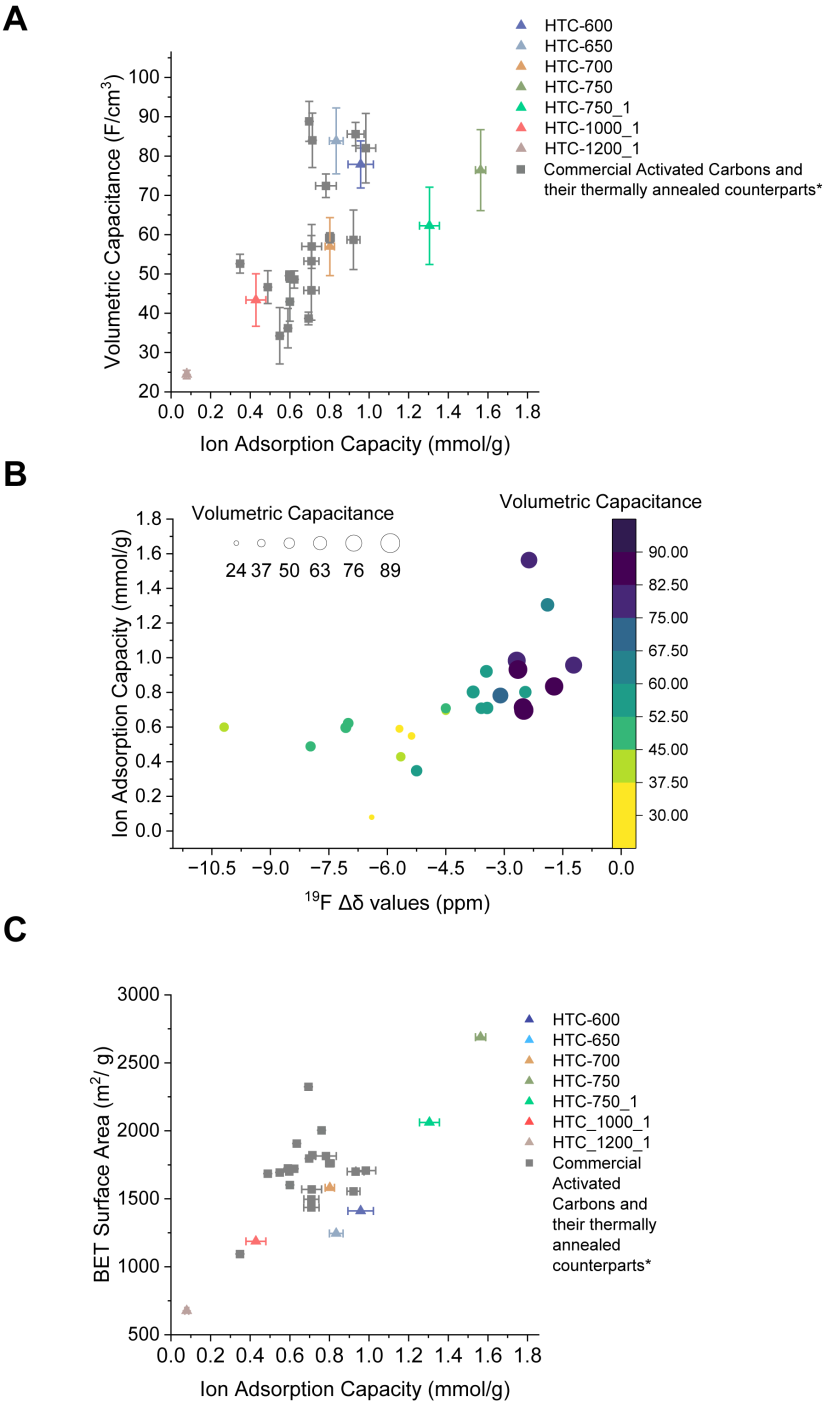

Figure S7: Relationship between volumetric capacitance and ion adsorption capacity. (A) Relationship between volumetric capacitance and ion adsorption capacity for the synthesised carbons series, with commercial activated carbons and their thermally annealed counterparts data added from previous studies.[2] **(B)** Relationship between volumetric capacitances at 0.05 A g^{-1} and ion adsorption capacity and ^{19}F Δδ values for the synthesised carbons series, with

commercial activated carbons and their thermally annealed counterparts data added from previous studies.[2] **(C)** Relationship between BET surface area and ion adsorption capacity for the synthesised carbons series, with commercial activated carbons and their thermally annealed counterparts data added from previous studies.[2] For carbons with extreme surface areas - very low (HTC-1200, ~676 m^2/g) and very high (HTC-750, ~2689 m^2/g) - BET surface area correlates well with ion adsorption capacity. However, for the majority of carbons clustering in the intermediate range (1400-2000 m^2/g), BET surface area shows no clear correlation with ion adsorption capacity, indicating that BET surface area alone is insufficient to predict electrochemically accessible porosity.

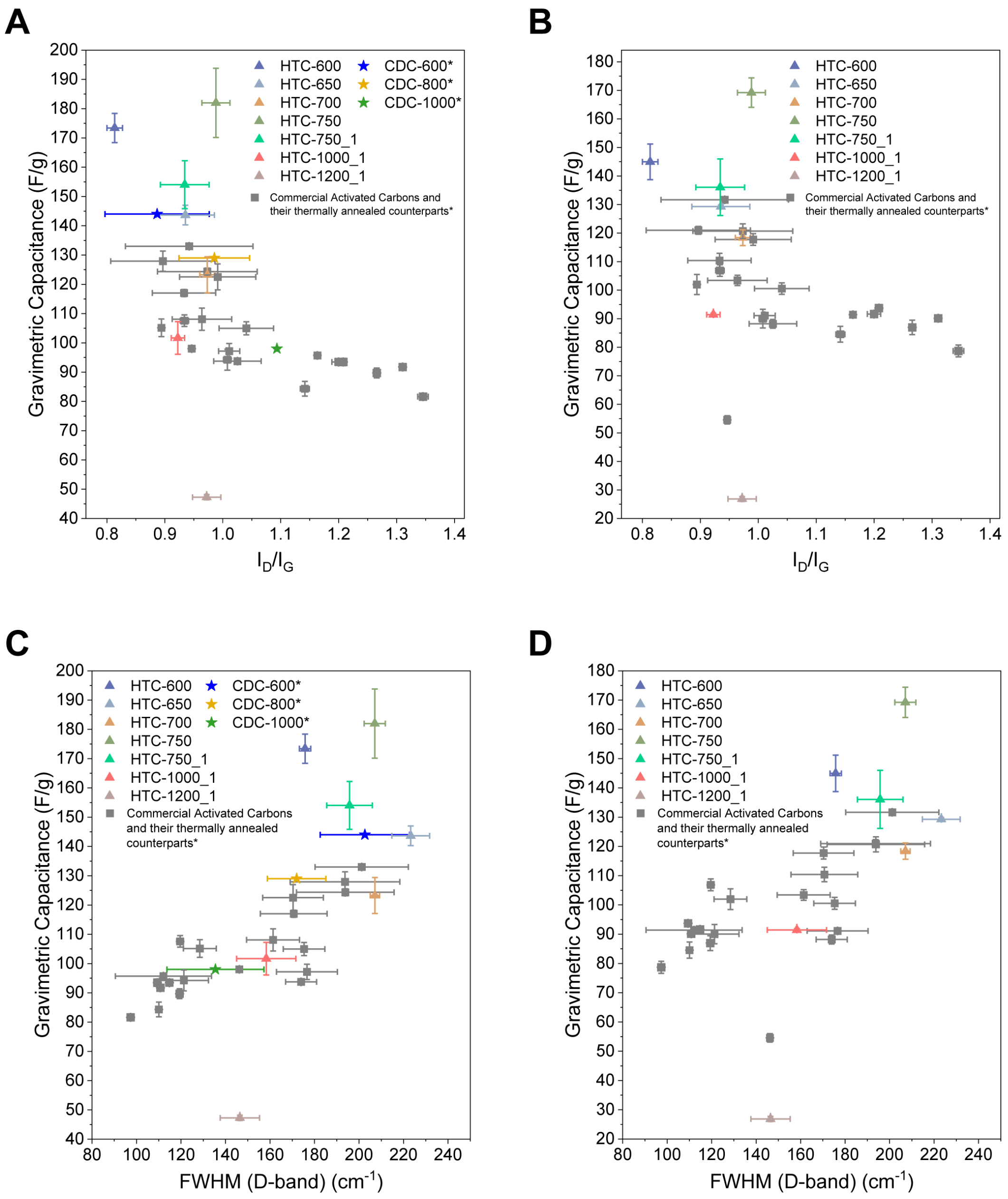

Figure S8: Relationship between gravimetric capacitance and I_D/I_G values and D-band full width half maximum (FWHM) from Raman. (A) Correlation between gravimetric capacitance at 0.05 A g^{-1} and I_D/I_G values for synthesised carbons, with commercial activated carbons and their thermally annealed counterparts data added from previous studies.[2,3] **(B)** Correlation between gravimetric capacitance at 1 A g^{-1} and I_D/I_G values for synthesised carbons, with commercial activated carbons and their thermally annealed counterparts data added from previous studies.[2,3] **(C)** Relationship between gravimetric capacitance at 0.05 A g^{-1} and D-band FWHM for the synthesised carbons, with commercial activated carbons and their thermally annealed counterparts data added from previous studies.[2,3]

(D) Relationship between gravimetric capacitance at 1 A g^{-1} and D-band FWHM for the synthesised carbons, with commercial activated carbons and their thermally annealed counterparts data added from previous studies.[2,3]

A

B

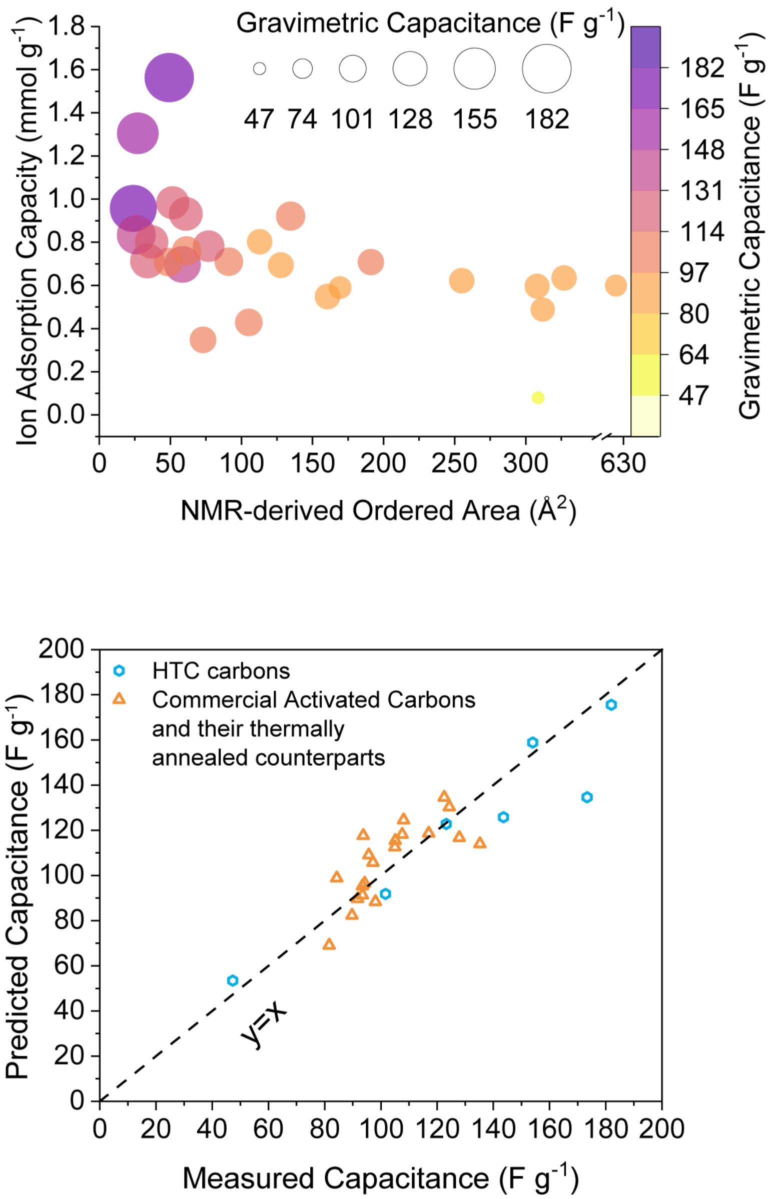

Figure S9: Relationship between ion adsorption capacity, NMR-derived ordered area and gravimetric capacitance and capacitance prediction. (A) Relationship between gravimetric capacitances at 0.05 A g^{-1} and ion adsorption capacities and NMR-derived ordered areas for the synthesised carbons series, with commercial activated carbons and their thermally annealed counterparts data added from previous studies.[2] **(B)** Relationship between predicted capacitance and measured capacitance using NMR-derived ordered area and ion adsorption capacity measured from NMR experiments.

Supplementary Tables

Table S1: BET surface area, accumulative pore volume and average pore size of all the synthesised carbons, derived from N_2 isotherms (77 K).

Carbon Name	BET Surface Area (m^2/g)	Accumulative Pore Volume (cc/g)	Average Pore Size (nm)
HTC-600	1411	0.64	1.07
HTC-650	1245	0.53	0.97
HTC-700	1581	0.65	0.80
HTC-750	2689	1.24	1.44
HTC-750_1	2062	0.93	1.05
HTC-1000_1	1187	0.48	0.80
HTC-1200_1	676	0.32	0.88

Table S2: %C and %O of part of the studied carbons, measured from X-ray photoelectron spectroscopy (XPS). The percentage represents atomic percent (at%).

Carbon	%C	%O	O-C (at%)	O=C (at%)
HTC-600	84.13	15.87	8.96	5.16
HTC-650	79.51	20.49	9.27	15.1
HTC-700	84.91	15.09	6.82	4.25
HTC-750	84.99	15.01	7.8	6.7
HTC-750_1	75.71	24.29	4.31	3.28
HTC-1000_1	91.05	8.95	4.25	6.72

Table S3: Table of elemental analysis (CHN combustion analysis) of the synthesised nanoporous carbons and two commercial activated carbons.

Carbon Name	%C (wt%)	%H (wt%)	%N (wt%)	Other elements (Possibly %O) (wt%)
HTC-600	85.97±0.11	3.36±0.07	0.28±0.01	10.40±0.04
HTC-650	85.23±0.01	2.72±0.04	0.32±0.01	11.72±0.05
HTC-700	88.64±0.01	2.22±0.01	0.46	8.68
HTC-750	91.97±0.03	1.94±0.01	0.27±0.01	5.38±0.04
HTC-750_1	88.23±0.01	2.63±0.03	0.28	8.87±0.04
HTC-1000_1	90.25±0.02	2.43±0.02	0.31±0.01	7.03±0.01
ACS-PC	93.23±0.06	0.57±0.03	0.44±0.01	5.74±0.03
EL-104	95.90±0.04	0.72±0.04	0.48±0.01	2.92±0.02

Table S4: Table of electrode mass loading in coin cells. Masses are averaged between two electrodes, with a standard deviation of ~0.1 mg for all cells.

Carbon Name	Electrode Mass Loading in cell 1 (mg/cm^2)	Electrode Mass Loading in cell 2 (mg/cm^2)
HTC-600	10.4	12.0
HTC-650	13.8	15.4
HTC-700	8.1	11.0
HTC-750	11.3	10.1
HTC-750_1	10.0	9.9
HTC-1000_1	10.6	10.1
HTC-1200_1	13.0	12.5

Table S5: Chemical shift of in-pore resonances and ^{19}F $\Delta\delta$ value for all studied carbons. The error in $\Delta\delta$ values represent the standard deviations of multiple fittings of the NMR spectra. For each fitting, different initial peak shapes with different Gaussian/Lorentzian ratios were applied, then varied freely to achieve the optimal fitting. Each spectrum was deconvoluted three times.

Carbon Name	In-pore resonance chemical shift (ppm)	^{19}F $\Delta\delta$ value (ppm)
HTC-600	-150.52±0.03	-1.22±0.03
HTC-650	-151.02±0.24	-1.72±0.24
HTC-700	-151.76±0.02	-2.46±0.02
HTC-750	-151.67±0.02	-2.37±0.02
HTC-750_1	-151.03±0.10	-1.73±0.10
HTC-1000_1	-154.95±0.07	-5.65±0.07
HTC-1200_1	-155.70±0.20	-6.40±0.20

Table S6: Cell resistance of the synthesised HTC carbons and two commercial activated carbons (ACS-PC and EL-104) in 1 M NEt_4BF_4 /ACN. The cell resistance is read from the IR drop in constant charge-discharge measurements. HTC-600 shows evidently larger resistance, suggesting the potential lower electrical conductivity compared to other synthesised carbons.

Carbon Name	Resistance of cell 1 (Ω)	Resistance of cell 2 (Ω)	Average (Ω)
HTC-600	150.5	158.20	154.35±5.44
HTC-650	18.36	23.80	21.08±3.85
HTC-700	17.48	25.60	21.54±5.74
HTC-750	61.70	67.04	64.37±3.78
HTC-750_1	36.51	22.72	29.62±9.75
HTC-1000_1	6.93	7.20	7.07±0.20
HTC-1200_1	12.90	15.20	14.05±1.63
ASC-PC	23.04	23.69	23.37±0.46
EL-104	29.50	36.00	32.75±4.60

Table S7: Conductivity from four-point probe measurements of the synthesised HTC carbon films and two commercial activated carbon films (ACS-PC and EL-104).

Carbon Name	Conductivity (S/m)
HTC-650_6	0.004±0.001
HTC_700_6	0.012±0.008
HTC_750_6	0.326±0.077
HTC_750_1	0.213±0.022
HTC_1000_1	0.832±0.155
HTC_1200_1	0.256±0.106
ACS-PC	1.22±0.10
EL-104	0.47±0.07

Supplementary References

1 Jagiello, J. & Olivier, J. P. 2D-NLDFT adsorption models for carbon slit-shaped pores with surface energetical heterogeneity and geometrical corrugation. *Carbon* **55**, 70-80 (2013). https://doi.org/10.1016/j.carbon.2012.12.011

2 Liu, X. Y. *et al.* Structural disorder determines capacitance in nanoporous carbons. *Science* **384**, 321-325 (2024). https://doi.org/10.1126/science.adn6242

3 Liu, X. Y. *et al.* Raman Spectroscopy Measurements Support Disorder-driven Capacitance in Nanoporous Carbons. *J Am Chem Soc* **146**, 30748-30752 (2024). https://doi.org/10.1021/jacs.4c10214
