# Supplementary material for: Highly disordered nanoporous carbons for enhanced energy storage in supercapacitors
Source: Nat Commun. 2026 Apr 20;17:5464. doi: 10.1038/s41467-026-71520-x (PMC13284229; doi:10.1038/s41467-026-71520-x)
Supplement: Supplementary file 2 — Transparent Peer Review file [file 41467_2026_71520_MOESM2_ESM.pdf]

nature portfolio

Peer Review File

Highly Disordered Nanoporous Carbons for Enhanced Energy Storage in Supercapacitors

Corresponding Author: Professor Alexander Forse

Parts of this Peer Review File have been redacted as indicated to maintain the confidentiality of unpublished data.

This file contains all reviewer reports in order by version, followed by all author rebuttals in order by version.

Version 0:

Reviewer comments:

Reviewer #1

(Remarks to the Author)
This manuscript presents a significant investigation into low-temperature synthesis route for the production of highly disordered nanoporous carbons, which are intended for supercapacitor applications. The authors effectively demonstrate that this approach can generate carbons with increased capacitance. This accomplishment contributes substantially to the ongoing endeavors of developing energy - efficient and scalable synthesis methods for supercapacitor electrodes. The integration of structural and electrochemical analyses offers valuable insights. However, to further augment the impact of the study, I propose the following considerations for the authors. With these revisions, the manuscript holds the potential to make a substantial contribution to the relevant literature. Therefore, I recommend a major revision prior to considering the work for publication.

(1) The authors previously published an impactful paper (Science, DOI: 10.1126/science.adn6242), which convincingly demonstrated the pivotal role of structural disorder in enhancing the capacitance of nanoporous carbons. Given that the current study shares conceptual resemblances, especially in emphasizing the significance of highly disordered carbon structures with small graphene - like domains, it is advisable for the authors to clearly demarcate the novel aspects of this work. Elucidating how the current findings expand upon or diverge from their earlier work would effectively highlight the scientific contribution of this study.

(2) The manuscript indicates that achieving highly disordered carbon structures through low - temperature synthesis represents a central innovation. If this is truly the key advancement, the authors might contemplate strengthening the introduction by providing more background information on why this synthetic challenge is of great importance, what specific limitations are inherent in conventional high - temperature approaches, and how their method surmounts these limitations. A more explicit elaboration of the technical difficulties and the innovative aspects of their method would enhance the perceived novelty and relevance of the work.

(3) The discussion on the ion-accessible surface area is engaging; however, its importance could be more firmly substantiated by quantitative data. Incorporating numerical comparisons that demonstrate how the pore structure affects ion adsorption under identical electrochemical conditions, for example, a consistent electrolyte composition and potential window, would fortify the conclusions drawn in the study. Moreover, visual aids like pore - size distributions presented in relation to ion transport metrics or surface accessibility ratios would offer readers a more intuitive understanding of the structure - performance relationship. This would not only enhance the clarity of the manuscript but also deepen the reader's comprehension of the underlying scientific concepts.

Reviewer #2

(Remarks to the Author)
This manuscript presents an insightful study showcasing the utility of NMR as a powerful tool to elucidate the relationship between capacitance and structural features in microporous carbons. The authors provide high-quality data and clear results that contribute meaningfully to the understanding of electrochemical performance in disordered carbon materials.
However, there are a few critical points that merit further discussion and clarification:

1. Trade-Off Between Capacitance and Stability: The authors advocate for low-temperature, low-domain-size carbons as a route to enhanced capacitance due to their higher structural disorder. While this is supported by the data, it is important to address the associated trade-offs. Increased disorder inherently raises the edge to in-plane atom ratio, potentially introducing more electrochemically active sites. This, in turn, could compromise electrolyte stability and narrow the electrochemical stability window (ESW). From an energy storage perspective, it is essential to assess whether the observed capacitance gains are sufficient to outweigh the losses in stability. A more detailed discussion of this trade-off would strengthen the manuscript.
2. Cyclability and Long-Term Performance: One of the defining advantages of supercapacitors is their exceptional cycle life, a benchmark that high-rate lithium-ion batteries still struggle to meet. If the structural disorder in low-temperature carbons promotes parasitic side reactions due to a higher density of reactive sites, this could potentially degrade cyclability. The manuscript would benefit from a more critical assessment of whether the short-term gains in capacitance are sustainable in the long term, especially under real-world cycling conditions.
3. Novelty and Justification of the Study: A broader concern relates to the novelty of the findings. The authors have previously demonstrated the impact of disorder on capacitance using commercial carbons. While synthesizing their own carbons adds an element of control and reproducibility, the central conclusion appears to reiterate their earlier claims. The manuscript would be considerably strengthened by a clearer articulation of what new insights this work provides beyond previous studies, and whether the incremental advancement justifies publication at the level of a high-impact journal such as Nature.

Reviewer #3

(Remarks to the Author)
This paper reports the synthesis of a highly disordered nanoporous carbon material prepared via low-temperature carbonization and activation. The material exhibits small graphene-like domain sizes and high capacitance. The authors claim to establish a correlation between the graphene-like domains and capacitance, which is consistent with findings reported in their previous work. However, the structural and electrochemical characterizations presented in the manuscript are insufficient. Therefore, I do not recommend acceptance of this paper.
1. More comprehensive characterization of the carbon structure is necessary to provide a full understanding. This should include quantification of domain size (both lateral and vertical thickness), interlayer spacing, and detailed pore structure analysis distinguishing between open and closed pores.
2. The manuscript lacks detailed electrochemical characterization. Cyclic voltammetry (CV) at varying scan rates, electrochemical impedance spectroscopy (EIS), and analysis of ion diffusion kinetics should be included to evaluate performance comprehensively.
3. While the materials show good rate performance, it is unclear how low-temperature synthesis achieve this. Conductivity measurements should be provided.
4. The low-temperature synthesis and high density of edge sites may introduce side reactions. These potential reactions and their impacts on electrochemical stability should be investigated and discussed.
5. In Figure S1, charge–discharge and CV tests were conducted over different voltage ranges. The rationale for using different potential windows should be clearly explained.
6. The manuscript should include a more in-depth discussion of the formation mechanism responsible for generating the small graphene-like domains under low-temperature conditions.
7. The SSA increases sharply from HTC-700 to HTC-750. This sudden change requires further discussion and a clearer explanation.
8. Although the overall oxygen content appears similar among the samples, the distribution and types of oxygen-containing functional groups likely differ. These differences should be analyzed and discussed in relation to performance. In addition, the XPS results in Table S2 show that HTC-650 has a significantly higher oxygen content than the other samples. This anomaly should be verified, and the variations in oxygen species across all samples should be interpreted and explained.

Reviewer #4

(Remarks to the Author)
This work investigated the electrical double-layer capacitance (EDLC) of nanoporous carbon materials, specifically, using NMR, from which, the authors tried to extend conclusions, including: 1) low-temperature synthesis is a promising pathway to produce nanoporous carbons with enhanced capacitance in EDLC; 2) Nanoporous carbons produced at low temperature have a smaller graphene-like domain; 3) Carbon with smaller graphene-like domains and higher ion adsorption capacity show enhanced EDLC; and 4) $\Delta\delta$ and the ion adsorption capacity both measured by NMR can be used as descriptors for capacitance. Among the above four points of conclusion, only the fourth one can mark this work a valuable investigation, while the other three are either worthless or unacceptable.
Conclusion item 1: Carbon materials produced at “low temperature” do not always render enhanced EDLC. Figures 1, 3, and 3 showed that HTC-750 outperformed HTC-600 in gravimetric capacitance. If the authors lower the heat treatment temperature down to 300 oC, and name the sample HTC-300. One can expect an even lower gravimetric capacity for the HTC-300 than for the HTC-600, which is likely due to the HTC-300's lower carbonization level. Therefore, conclusion item 1 is misleading to readers, particularly those unfamiliar with this topic.

Conclusion item 2: Carbon materials, including nanoporous carbon, produced at a lower temperature always have a smaller graphene-like domain if the other synthesis conditions are kept identical. A larger graphene-like domain indicates a higher level of graphitization; graphitization is an endothermic reaction process, which requires a higher temperature or extended

heat treatment time. Low-temperature heating treatment yields a lower graphitization level of pyrolytic carbon produced from the same precursor. This is widely accepted. Conclusion item 2 contributes nothing new to the knowledge.

Conclusion item 3: It is questionable that carbon with smaller graphene-like domains shows enhanced EDLC. The pore size relevant to the ion size is a critical factor that dominates the EDLC. Figure 1b shows that HTC-750 is rich in subnanometer pores compared to HTC-700, -650, and -600. Figure 1e, 1f, 2a, and 2b show that HTC-750 has the highest gravimetric capacity. Considering that the size of TEA ion is approximately 0.45 nm, the subnanopore in HTC-750 should be a key factor that yields the best gravimetric capacity. The critical role of pore size on the EDLC was first reported by Prof. Simmon and Gogotsi (Science 2006), and this phenomenon has been reported in the following research by different research groups. Unfortunately, one can barely find the discussion on the effect of pore size in the manuscript, even the HTC-750 showed clearly a different pore size distribution (Fig 1b).

Conclusion item 4: This is the most valuable conclusion. Insightful and practically beneficial. However, this conclusion still requires a more comprehensive study. For example, applying carbon materials from a larger variety of sources to present the viability of this method, and illustrate the physical picture at the microscale to allow readers who are not experts in NMR to understand its fundamentals easily.

Version 1:

Reviewer comments:

Reviewer #1

(Remarks to the Author)
Authors have well solved the comments

Reviewer #2

(Remarks to the Author)
The authors have addressed all my comments and concerns satisfactorily, and I recommend publication of the article.

Reviewer #5

(Remarks to the Author)
The article “Highly disordered nanoporous carbons for enhanced energy storage in supercapacitors” reports a study on KOH-activated carbons used as electrodes and tested with an organic electrolyte for supercapacitor performance. Authors correlate the electrochemical test parameters with NMR, Raman spectroscopy and nitrogen gas sorption data. While the findings are interesting and bring important factor (temperature) for carbon preparation again to discussion, this work considerably lacks novelty. Authors use a relatively low temperature treatment of organic precursors with KOH to produce activated carbons with disorder. Authors state that they do not find a correlation between pore size and capacitance values of the carbon materials, contrary to previously well-established concept. As a matter of fact, activated carbons, both powder and cloth are very sensitive material due to the edge sites and dangling bonds rendering them vulnerable to fast reaction with oxygen and moisture from the atmosphere. This causes the generation of oxygenated functional groups on the surface and of course block the pores entrances. The degree of functionalization depends on how long the carbon sample was kept in open or closed environment. It is therefore, necessary to perform heat treatment ~700°C before using any carbon material in a supercapacitor device (especially with non-aqueous electrolyte). If ignored, the pore blockage of non-treated carbon could lead to significant capacitance discrepancies in the obtained electrochemical performance.

Some of the works on surface functional groups are listed below.

https://pubs.acs.org/doi/10.1021/ie061071v Characterization of Active Sites on Carbon Catalysts | Industrial & Engineering Chemistry Research
https://www.sciencedirect.com/science/article/abs/pii/S0008622398003339 Modification of the surface chemistry of activated carbons - ScienceDirect
https://www.sciencedirect.com/science/article/abs/pii/S0008622308006696 Effect of electrochemical treatments on the surface chemistry of activated carbon - ScienceDirect
https://www.sciencedirect.com/science/article/pii/S0957582021000707 Feasibility of electrochemical regeneration of activated carbon used in drinking water treatment plant. Reactor configuration design at a pilot scale - ScienceDirect
https://link.springer.com/article/10.1007/s10800-013-0644-0 Factors contributing to ageing of high voltage carbon/carbon supercapacitors in salt aqueous electrolyte | Journal of Applied Electrochemistry
https://www.sciencedirect.com/science/article/abs/pii/S0013468620308082 Fitting the porous texture of carbon electrodes to a binary ionic liquid electrolyte for the realization of low temperature EDLCs - ScienceDirect

Another issue is the degree of disorder in carbon sample activated at 750°C compared to other samples. The significant difference is of course due to the higher temperature which is more favourable for introducing long-range graphene layers. Further high temperature such as 1000°C or 1200°C would lead to graphitization of material which is generally used for producing hard carbons and is already a known fact. However, in the context of this manuscript, this abrupt change of structure for the sample produced at 750°C has not been explained sufficiently and scientifically.

Some suggestions on role of pore size distribution are listed below.
• From the pore size distribution data, authors could calculate the fraction of pore volume for micro-, ultra-micro-, and mesopores that would reveal whether improved capacitance arises primarily from structural disorder or from differences in accessible pore volume.
• To what extent might pore size distribution influence the NMR-derived descriptors (Δδ and ion adsorption capacity)? Smaller pores may enhance adsorption capacity but limit ion mobility; conversely, larger pores may facilitate faster transport but reduce volumetric performance. These effects could influence the interpretation of the NMR data.
• Are the observed performance differences attributable solely to graphene-like domain size, or could pore size and connectivity be co-contributing factors? Without PSD data, it is difficult to disentangle the relative contributions of domain disorder versus pore structure.
• How does the pore size distribution of these materials relate to tortuosity metrics discussed in their earlier Nature Materials study? For example, carbon with high microporosity but lower mesopore volume might inherently exhibit higher tortuosity, which could influence rate capability even if intrinsic capacitance is high.
• Correlating the authors finding with pore size distribution of carbons preferably would allow readers to better align the present results with the established understanding of pore-network effects from the author’s earlier work. This would also strengthen the mechanistic interpretation of the structure–property relationships and provide a more complete picture of how disorder, pore accessibility, and pore geometry collectively determine capacitive performance.

Authors should show the morphology of these carbons such as with TEM data to clarify how the graphene domains are oriented in each carbon sample. Such data also helps to calculate adjacent graphene layer distance and their orientation. In some of the works given below, it is clearly shown how the graphene layers are arranged leading to the pore development.

https://www.sciencedirect.com/science/article/abs/pii/S0008622320307399 Melting point depression of ionic liquids by their confinement in carbons of controlled mesoporosity - ScienceDirect
https://link.springer.com/article/10.1007/s11581-021-04354-w Revisiting the carbon mesopore contribution towards improved performance of ionic liquid–based EDLCs at sub-zero temperatures | Ionics

A similar data would definitely help in understanding the organization of graphene domains and pore development by the short-range and long-range graphene layers which is important for finding a correlation with how the charges are stored and how capacitance increases.

It would be unfair to rule out the role of pore size in the electrochemical performance of supercapacitors without presenting three-electrode cell data. The electrochemical performance lacks information on individual carbon electrodes in the given organic electrolyte where one could see the charge/discharge behaviour of carbon electrodes on positive and negative sides of the supercapacitor cell to clarify how the size of pores affects the capacitances on each polarity.

Based on the above-stated reasons and lack of novelty, I do not recommend this article for publishing in Nature Communications.

Version 2:

Reviewer comments:

Reviewer #6

(Remarks to the Author)
Reviewer comments:

In this article the authors generate design descriptors and performance prediction tools for the application of nanoporous carbons in organic supercapacitors. The authors synthesize nanocarbons with varying features by tuning the synthesis conditions, most notably the temperature used during the KOH activation step. This route enabled the preparation of porous carbons with varying graphene-like domain sizes, a parameter that was demonstrated alongside the ion adsorption capacity to have a significant impact on the resulting gravimetric capacitance. The authors utilized a NMR route to quantitatively characterize both the graphene-like domain sizes and the ion adsorption capacity of the carbon materials when soaked in electrolyte, enabling them to predict the resulting performance of the materials using these two quantitative parameters as inputs for their prediction tool. This work is well-done and the manuscript is written in a way that is clear, concise and accessible to both experts and non-experts alike. As I was asked to focus on the response to comments from Reviewer #5, I will focus my review on that.

The majority of the comments from Reviewer #5 were objective and scientific/technical in nature. It is my opinion that the authors have provided responses to these comments that are rigorous, clear and scientifically compelling. The authors addressed many of the comments either by adding new data, or by drawing attention to the previously included data and analyses that addressed the Reviewer’s points but were perhaps overlooked in the initial review. For other comments, the authors reasonably rebutted making significant changes to the manuscript, including explanations and citations to the literature to justify as appropriate. The authors also made several revisions to the writing to improve clarity and provide more detail.

The first comment from Reviewer #5 was more subjective in nature and related to the novelty of the work. I completely agree

that the synthesis strategies developed in this work and that changing the temperature and synthetic conditions used to prepare carbon impacts the degree of disorder and chemical features has been widely reported before. However, I do agree with the authors point that a key aspect of novelty of this work is the demonstration of the "dual-factor performance model", in that both ion adsorption capacity and graphene-like domain sizes dictate the capacitance of the materials. Perhaps even more importantly, the authors demonstrate a route to quantitatively simultaneously evaluate both of these properties using NMR, which can then be used to predictively determine the capacitance. These are most certainly novel demonstrations.

To this end, I recommend acceptance of this work in Nature Communications as it is a very well done and well written study, and the authors have provided suitable responses to Reviewer #5. If I may, I have two suggested comments for the authors to consider addressing when making the final revisions to this work, as I think it will help clarify some of the key concepts explored in this paper.

1. The authors show the independent impacts of ion adsorption capacity and the size of graphene-like domains on the resulting capacitance of the materials. However, these two parameters are closely coupled, which even the authors acknowledge in Line 295 when they state: "We hypothesise that edge sites accessible to electrolyte ions are where the charges are stored most efficiently". In other words, carbons with smaller sizes of graphene-like domains will have more edge sites and by extension more ion adsorption capacity. However, the subsequent discussion and majority of the paper discuss these two parameters in a way that they are considered independent from each other, for example line 297: ""Summarising, the ion adsorption capacity plays an important role together with the sizes of the graphene-like domains in determining the capacitance." To their credit, the authors did a good job demonstrating the synthesis of carbon particles where they could independently vary one of these parameters without significantly changing the other. Thus, there is some sort of "loose" interdependency between these two parameters (graphene-like domain size and ion exchange capacity), where in some cases they are dependent on each other but in other cases they are not. It would likely benefit the readers if the authors could include a few sentences that succinctly explain and clarify the relationship between these two parameters, as I think that will really help readers interpret the results and further appreciate the achievements of this work. Particularly, providing a brief discussion of how ion adsorption capacity and graphene-like domain sizes are related to each other in some instances, but then can deviate from each other in other instances, would be helpful.

2. I would suggest providing a bit more detail and clarification regarding the mechanism for porosity formation within the carbon particles during synthesis. In particular, the authors state: "During the KOH activation process, KOH decomposes into K2CO3 around 400°C, which further breaks down into K2O and CO2 at temperatures above 700°C, leading to activated carbons with high BET surface areas and development of pores around 2 nm diameter." In the way this is currently written, it may be unclear to readers how exactly the formation of K2O, CO2, and K2CO3 results in porosity formation within the carbon particles, as those seem like they could be two independent processes. I suggest providing a sentence to explain how the formation of these species is mechanistically linked with the formation of porosity in the carbon particles so that readers do not need to connect the dots themselves.

Open Access This Peer Review File is licensed under a Creative Commons Attribution 4.0 International License, which permits use, sharing, adaptation, distribution and reproduction in any medium or format, as long as you give appropriate credit to the original author(s) and the source, provide a link to the Creative Commons license, and indicate if changes were made.

In cases where reviewers are anonymous, credit should be given to 'Anonymous Referee' and the source.
The images or other third party material in this Peer Review File are included in the article's Creative Commons license, unless indicated otherwise in a credit line to the material. If material is not included in the article's Creative Commons license and your intended use is not permitted by statutory regulation or exceeds the permitted use, you will need to obtain permission directly from the copyright holder.
To view a copy of this license, visit https://creativecommons.org/licenses/by/4.0/

Response to Reviewer 1

This manuscript presents a significant investigation into low-temperature synthesis route for the production of highly disordered nanoporous carbons, which are intended for supercapacitor applications. The authors effectively demonstrate that this approach can generate carbons with increased capacitance. This accomplishment contributes substantially to the ongoing endeavors of developing energy - efficient and scalable synthesis methods for supercapacitor electrodes. The integration of structural and electrochemical analyses offers valuable insights. However, to further augment the impact of the study, I propose the following considerations for the authors. With these revisions, the manuscript holds the potential to make a substantial contribution to the relevant literature. Therefore, I recommend a major revision prior to considering the work for publication.

We thank the reviewer for their comments and suggestions. All revisions made in the manuscript are highlighted in yellow for clarity. Our detailed responses to each comment are provided below.

(1) The authors previously published an impactful paper (Science, DOI: 10.1126/science.adn6242), which convincingly demonstrated the pivotal role of structural disorder in enhancing the capacitance of nanoporous carbons. Given that the current study shares conceptual resemblances, especially in emphasizing the significance of highly disordered carbon structures with small graphene - like domains, it is advisable for the authors to clearly demarcate the novel aspects of this work. Elucidating how the current findings expand upon or diverge from their earlier work would effectively highlight the scientific contribution of this study.

While our previous Science paper (*Science* **2024**, https://www.science.org/doi/10.1126/science.adn6242) established the fundamental principle that structural disorder drives capacitance using commercial carbons, the current work represents a major advancement in three critical areas:

1. **Rational Synthesis Strategy:** We now provide a systematic synthetic route to produce highly disordered carbons, moving from discovery to practical implementation. This bridges the gap between fundamental understanding and materials engineering.

2. **Dual-Factor Performance Model:** Our previous work discovered that domain sizes dominate capacitance in nanoporous carbons. With a wider range of carbons, here we reveal that both graphene-like domain sizes and ion adsorption capacities determine capacitance, providing a more complete mechanistic understanding, which has not been reported in previous literature and extends the conclusion in our previous study.

3. **Data-Driven Prediction:** We establish the first quantitative method to predict capacitance directly from NMR spectra, transforming NMR from a characterisation tool into a materials screening platform. This enables rapid identification of high-performance materials without electrochemical testing.

As described in our original manuscript:

In abstract: *"NMR spectroscopy measurements provided unique insights by simultaneously probing local structural order and ion adsorption capacities, revealing that carbons with smaller graphene-like domain sizes and higher ion adsorption capacities generally have better capacitive performance."*

In the main text: *"Importantly, in addition to providing a measurement of the sizes of the graphene-like domains, our NMR experiments measure another key property of the nanoporous carbons; the electrolyte ion adsorption capacity. Indeed, the integrals of the in-pore resonances relate to the number of ions which can access the carbon nanopores in the absence of applied potential. Deconvolution of the NMR spectra of the electrolyte-saturated carbon samples shows that the gravimetric capacitance showed some correlation with the ion adsorption capacity (Figure 1f), with HTC-750 having the largest ion adsorption capacity in the series (1.6 mmol g^{-1})."*

In conclusion: *"NMR spectroscopy, in particular, allowed us to simultaneously evaluate both the sizes of the graphene-like domains and the electrolyte ion adsorption capacities, with electrolyte ions as the probe. Our findings indicate that carbons with smaller graphene-like domains and higher ion adsorption capacity generally show enhanced capacitive performance. The highest gravimetric capacitances were achieved for either highly disordered carbons or carbons with more order but more accessible pores (higher electrolyte ion adsorption capacities), the latter carbons, however, generally being associated with lower volumetric capacitances. We show that the combination of $\Delta\delta$ and the ion adsorption capacity both measured by NMR spectroscopy in a quick and simple NMR experiment (~5 mins) are excellent descriptors for capacitance, enabling rapid identification of high-performance materials without electrochemical testing"*

At the reviewer's suggestion, we have addressed this in the main text and conclusion:

"With an expanded series of nanoporous carbons including those from our previous studies, it is observed that carbons with higher gravimetric capacitances generally have (i) larger ion adsorption capacities (Figure 3a) and (ii) $\Delta\delta$ values of smaller magnitude (Figure 3b), which identifies ion adsorption capacity as another key descriptor for capacitance and extends our previous findings.[21]"

"We show that the combination of $\Delta\delta$ and the ion adsorption capacity both measured by NMR spectroscopy in a quick and simple NMR experiment (~5 mins) are excellent descriptors for capacitance, enabling rapid identification of high-performance materials without electrochemical testing."

(2) The manuscript indicates that achieving highly disordered carbon structures through low - temperature synthesis represents a central innovation. If this is truly the key advancement, the authors might contemplate strengthening the introduction by providing more background information on why this synthetic challenge is of great importance, what specific limitations are inherent in conventional high - temperature approaches, and how their method surmounts these limitations. A more explicit elaboration of the technical difficulties and the innovative aspects of their method would enhance the perceived novelty and relevance of the work.

We appreciate this suggestion and will expand the introduction to better state the significance of our approach. As described above, the key innovation is not the development of new synthesis methods, but rather the strategic application of established low-temperature activation guided by our fundamental understanding of disorder effects.

Carbon synthesis for supercapacitors has extensively focused on optimising the pore structure (*Energy Fuels* **2023**, *37*, (5), 3523–3554), while overlooking structural disorder - a factor our previous work demonstrated to be the primary determinant of capacitance performance. While it has been studied that low-temperature treatment leads to carbons with smaller graphene-like domains (*Carbon* **2012**, *50*, (13), 4880-4886), here we systematically exploit established low-temperature KOH activation (600-750 °C) to engineer maximum structural disorder while maintaining adequate porosity, presenting a fundamental shift from porosity-centred to disorder-centred design. Extended activation times (6 hours) overcome the traditional yield limitations of lower-temperature processes while preserving the small graphene-like

domains essential for enhanced performance. Our work demonstrates how fundamental mechanistic insights can transform materials synthesis strategy, even using well-established chemical processes. The innovation lies in recognising disorder as the critical design parameter and strategically applying synthesis conditions to optimise this previously neglected factor.

At the reviewer's comment, we have addressed this in the main text:

"While low-temperature-treated carbons exhibit smaller graphene-like domains,[35] previous studies of supercapacitor electrodes have focused on optimising the pore structure and have largely overlooked domain size[18] -a factor shown to be a primary determinant of capacitance performance. We here synthesise a range of highly disordered activated carbons and find that their high capacitances correlate with their small graphene-like domain sizes, while the electrolyte accessibility to the carbon pores also plays an important role, presenting a fundamental shift from porosity-centred to disorder-centred design.[36,37] Our work shows that low-temperature synthesis is a promising method for producing nanoporous activated carbons with a more disordered local structure and enhanced energy storage in EDLCs and demonstrates how fundamental mechanistic insights can transform materials synthesis strategy, even using well-established chemical processes, offering a clear principle for designing nanoporous carbons with better performance."

(3) The discussion on the ion-accessible surface area is engaging; however, its importance could be more firmly substantiated by quantitative data. Incorporating numerical comparisons that demonstrate how the pore structure affects ion adsorption under identical electrochemical conditions, for example, a consistent electrolyte composition and potential window, would fortify the conclusions drawn in the study. Moreover, visual aids like pore - size distributions presented in relation to ion transport metrics or surface accessibility ratios would offer readers a more intuitive understanding of the structure - performance relationship. This would not only enhance the clarity of the manuscript but also deepen the reader's comprehension of the underlying scientific concepts.

At the reviewer's suggestion, we have included the relationship between BET surface area from nitrogen gas sorption measurements and ion adsorption capacity measured by NMR in the Supporting Information Figure S7 (C):

C

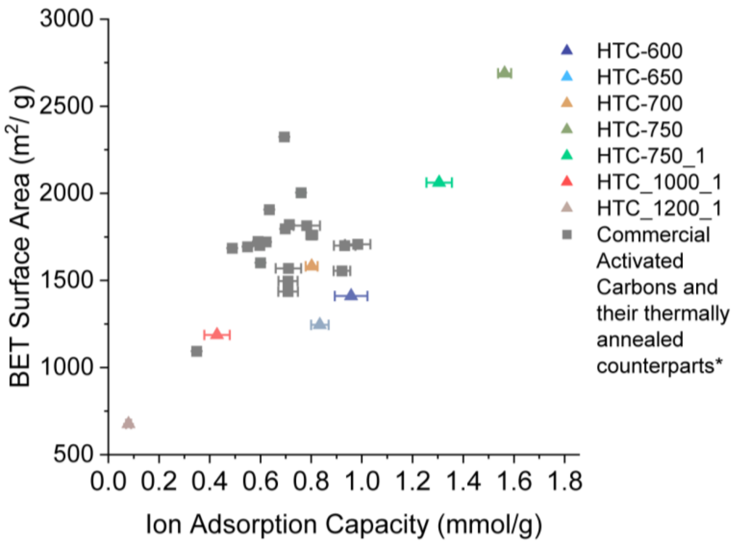

***(C)** Relationship between BET surface area and ion adsorption capacity for the synthesised carbons series, with commercial activated carbons and their thermally annealed counterparts data added from previous studies.[2] For carbons with extreme surface areas - very low (HTC-1200, ~676 m^2/g) and very high (HTC-750, ~2689 m^2/g) - BET surface area correlates well with ion adsorption capacity. However, for the majority of carbons clustering in the intermediate range (1400-2000 m^2/g), BET surface area shows no clear correlation with ion adsorption capacity, indicating that BET surface area alone is insufficient to predict electrochemically accessible porosity.*

We have addressed this in the main text:

"While there is some correlation between BET surface area and NMR-measured ion adsorption capacity for the HTC samples (Figure S7C), these quantities do not correlate well for commercial activated carbons. This suggests that BET surface area alone is insufficient to predict electrochemically accessible porosity, underscoring the value of the NMR approach over gas sorption-based methods."

Response to Reviewer 2

This manuscript presents an insightful study showcasing the utility of NMR as a powerful tool to elucidate the relationship between capacitance and structural features in microporous carbons. The authors provide high-quality data and clear results that contribute meaningfully to the understanding of electrochemical performance in disordered carbon materials.

We thank the reviewer for their positive comments. All revisions made in the manuscript are highlighted in yellow for clarity. Our detailed responses to each comment are provided below.

However, there are a few critical points that merit further discussion and clarification: 1. Trade-Off Between Capacitance and Stability: The authors advocate for low-temperature, low-domain-size carbons as a route to enhanced capacitance due to their higher structural disorder. While this is supported by the data, it is important to address the associated trade-offs. Increased disorder inherently raises the edge to in-plane atom ratio, potentially introducing more electrochemically active sites. This, in turn, could compromise electrolyte stability and narrow the electrochemical stability window (ESW). From an energy storage perspective, it is essential to assess whether the observed capacitance gains are sufficient to outweigh the losses in stability. A more detailed discussion of this trade-off would strengthen the manuscript.

We acknowledge that our low-temperature carbons show a measurable capacitance-stability trade-off, but the fundamental principle and engineering potential justify this proof-of-concept study.

Comparing our highest-performing synthesised carbon (HTC-750) with the best commercial activated carbon (ACS-PC), we observe a 36% capacitance increase (182 vs. 133 F/g) while HTC-750 degrades approximately 1.8 times faster. All other synthesised carbons show smaller capacitance gains with proportionally higher stability losses, as shown in Figure S2 in our original supporting information

However, we propose that our work serves as a fundamental proof-of-concept demonstrating that disorder engineering can achieve substantial capacitance gains. The observed stability trade-offs do not invalidate the approach but rather highlight engineering challenges that are addressable through established strategies, which lie beyond the scope of the current study. Future work should include: (1) detailed degradation mechanism analysis, (2) electrode optimisation through mass balancing and composite approaches, and (3) electrolyte engineering to minimise parasitic reactions at disordered sites. This work establishes the fundamental viability of disorder-driven design. The stability challenges we identify provide clear targets for future engineering efforts.

As described in the original manuscript:

"Secondly, the low-temperature synthesised carbons were found to have reduced cycling stability compared to commercial activated carbons (Figure S2). Floating tests were conducted to study the cycle lives (See Methods) of the low-temperature synthesised carbons and showed more rapid degradation compared to commercial activated carbons (Figure S2)."

"The conductivity and degradation issues can potentially be addressed in the future by incorporating conductive additives, e.g. carbon black,[41] and by electrode mass balancing,[42] respectively, to improve the overall performance of the devices. We propose that the concept of low-temperature activated carbon synthesis can be extended beyond the specific methods used in this study e.g. by using a range of biomass precursors and activation methods."

At the reviewer's suggestion, we have included additional discussions in the main text:

"*While our low-temperature synthesised carbons show reduced cycling stability compared to commercial materials (e.g., HTC-750 degrades about 1.8 times faster than ACS-PC despite achieving 36% higher capacitance), this work serves as a fundamental proof-of-concept demonstrating that disorder engineering can achieve substantial performance gains. This work establishes disorder engineering as a viable design strategy, with the identified stability challenges providing clear targets for future electrode optimisation and electrolyte engineering efforts*."

Additionally, the importance of long-term cycling stability depends significantly on the intended application. For certain supercapacitor applications such as regenerative braking systems in electric vehicles, power smoothing in renewable energy systems, backup power for memory devices, or pulse power applications (*Energy Environ. Sci.*, **2012**, (*5*), 7151-7160), the devices experience relatively infrequent cycling or operate primarily in standby mode, making the enhanced energy density more important than cycle life. In contrast, applications requiring continuous high-frequency cycling, such as grid-scale frequency regulation, would benefit more from the stability optimisation strategies we have identified for future development.

At the reviewer's suggestion, we have included additional discussion in the main text:

"The significance of cycling stability is application-dependent, with supercapacitor applications such as regenerative braking in electric vehicles, backup power systems, and pulse power delivery experiencing relatively infrequent cycling[42] where enhanced energy density may outweigh stability concerns. Applications that require continuous cycling would benefit from the stability enhancement strategies identified for future development."

2. Cyclability and Long-Term Performance: One of the defining advantages of supercapacitors is their exceptional cycle life, a benchmark that high-rate lithium-ion batteries still struggle to meet. If the structural disorder in low-temperature carbons promotes parasitic side reactions due to a higher density of reactive sites, this could potentially degrade cyclability. The manuscript would benefit from a more critical assessment of whether the short-term gains in capacitance are sustainable in the long term, especially under real-world cycling conditions.

Please see our response to the comment above.

3. Novelty and Justification of the Study: A broader concern relates to the novelty of the findings. The authors have previously demonstrated the impact of disorder on capacitance using commercial carbons. While synthesizing their own carbons adds an element of control and reproducibility, the central conclusion appears to reiterate their earlier claims. The manuscript would be considerably strengthened by a clearer articulation of what new insights this work provides beyond previous studies, and whether the incremental advancement justifies publication at the level of a high-impact journal such as Nature.

While our previous Science paper (*Science* **2024,** https://www.science.org/doi/10.1126/science.adn6242) established the fundamental principle that structural disorder drives capacitance using commercial carbons, the current work represents a major advancement in three critical areas:

1. **Rational Synthesis Strategy:** We now provide a systematic synthetic route to produce highly disordered carbons, moving from discovery to practical implementation. This bridges the gap between fundamental understanding and materials engineering.

2. **Dual-Factor Performance Model:** Our previous work discovered that domain sizes dominate capacitance in nanoporous carbons. With a wider range of carbons, here we reveal that both graphene-like domain sizes and ion adsorption capacities determine capacitance, providing a more complete mechanistic understanding, which has not been reported in previous literature and extends the conclusion in our previous study.

3. **Data-Driven Prediction:** We establish the first quantitative method to predict capacitance directly from NMR spectra, transforming NMR from a characterisation tool into a materials screening platform. This enables rapid identification of high-performance materials without electrochemical testing.

As described in our original manuscript:

In abstract: *"NMR spectroscopy measurements provided unique insights by simultaneously probing local structural order and ion adsorption capacities, revealing that carbons with smaller graphene-like domain sizes and higher ion adsorption capacities generally have better capacitive performance."*

In the main text: *"Importantly, in addition to providing a measurement of the sizes of the graphene-like domains, our NMR experiments measure another key property of the nanoporous carbons; the electrolyte ion adsorption capacity. Indeed, the integrals of the in-pore resonances relate to the number of ions which can access the carbon nanopores in the absence of applied potential. Deconvolution of the NMR spectra of the electrolyte-saturated carbon samples shows that the gravimetric capacitance showed some correlation with the ion adsorption capacity (Figure 1f), with HTC-750 having the largest ion adsorption capacity in the series (1.6 mmol g^{-1})."*

In conclusion: *"NMR spectroscopy, in particular, allowed us to simultaneously evaluate both the sizes of the graphene-like domains and the electrolyte ion adsorption capacities, with electrolyte ions as the probe. Our findings indicate that carbons with smaller graphene-like domains and higher ion adsorption capacity generally show enhanced capacitive performance. The highest gravimetric capacitances were achieved for either highly disordered carbons or carbons with more order but more accessible pores (higher electrolyte ion adsorption capacities), the latter carbons, however, generally being associated with lower volumetric capacitances. We show that the combination of $\Delta\delta$ and the ion adsorption capacity both measured by NMR spectroscopy in a quick and simple NMR experiment (~5 mins) are excellent descriptors for capacitance, enabling rapid identification of high-performance materials without electrochemical testing"*

At the reviewer's suggestion, we have addressed this in the main text and conclusion:

"With an expanded series of nanoporous carbons including those from our previous studies, it is observed that carbons with higher gravimetric capacitances generally have (i) larger ion adsorption capacities (Figure 3a) and (ii) $\Delta\delta$ values of smaller magnitude (Figure 3b), which identifies ion adsorption capacity as another key descriptor for capacitance and extends our previous findings.[21]"

"We show that the combination of $\Delta\delta$ and the ion adsorption capacity both measured by NMR spectroscopy in a quick and simple NMR experiment (~5 mins) are excellent descriptors for capacitance, enabling rapid identification of high-performance materials without electrochemical testing."

Response to Reviewer 3

This paper reports the synthesis of a highly disordered nanoporous carbon material prepared via low-temperature carbonization and activation. The material exhibits small graphene-like domain sizes and high capacitance. The authors claim to establish a correlation between the graphene-like domains and capacitance, which is consistent with findings reported in their previous work. However, the structural and electrochemical characterizations presented in the manuscript are insufficient. Therefore, I do not recommend acceptance of this paper.

We thank the reviewer for their comments. All revisions made in the manuscript are highlighted in yellow for clarity. Our detailed responses to each comment are provided below.

1. More comprehensive characterization of the carbon structure is necessary to provide a full understanding. This should include quantification of domain size (both lateral and vertical thickness), interlayer spacing, and detailed pore structure analysis distinguishing between open and closed pores.

The characterisation we have in the original draft is comprehensive to support the current conclusion without redundancy. We have characterised the domain sizes (in-plane correlation length) based on our NMR measurements combined with simulations. As described in the main text of our original manuscript:

"To extract the sizes of the graphene-like domains in the synthesised nanoporous carbons, we conducted NMR simulations based on our previously established method,[24,30] and as applied in previous studies[21] (Figure 3c). In general, the synthesised carbons follow the trend observed in our previous study that carbons with smaller calculated ordered domain sizes generally have higher capacitance.[21] Here our new low-temperature synthesised carbons have both smaller graphene-like domains and larger capacitances compared to our previous commercial activated carbon series, extending the limits of our master plots (Figure 3c). Among the carbons carbonised and activated for six hours, the ordered domain sizes increased as the synthesis temperature increased, e.g. from 23.9 Å^2 to 49.2 Å^2 for HTC-600 and HTC-750, respectively. HTC-1000_1 exhibits similar behaviour to most commercial carbons, while HTC-1200_1 with an intermediate simulated ordered area of around 308 Å^2 shows the lowest capacitance among all studied carbons, due to limited porosity and ion adsorption capacity (Figure S3, Table S1)."

For very disordered nanoporous carbons with in-plane correlation length less than 2 nm in the study, it is challenging to quantify the in-plane correlation length or the interlayer spacing between the disordered graphitic layers with other characterisation techniques due to the lack of crystallinity (*Carbon* **2006**, *44* (15), 3239-3246).

We have provided the pore structure analysis in Figure S3 in our original Supporting Information. While we acknowledge that closed pores are important in hard carbons for Na-ion batteries, for EDLCs that store charge based on the electrosorption of ions within the pores, ions do not access closed pores and therefore these pores cannot participate in charge storage. This is observed in the limited capacitance for conventional carbon black (*Carbon*, **2018**,133, 1-5).

The NMR-derived ion adsorption capacity directly measures electrolyte ion accessibility to carbon pores in the absence of applied potential, providing a more powerful predictor of electrochemical performance than BET surface area or geometric pore size analysis.

At the reviewer's suggestion, we have included the relationship between BET surface area from nitrogen gas sorption measurements and ion adsorption capacity measured by NMR in the Supporting Information Figure S7 **(C)**:

C

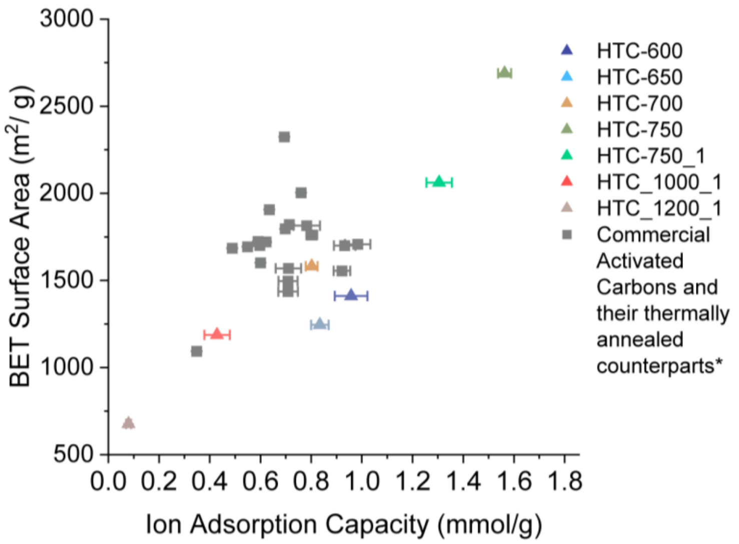

***(C)** Relationship between BET surface area and ion adsorption capacity for the synthesised carbons series, with commercial activated carbons and their thermally annealed counterparts data added from previous studies.[2] For carbons with extreme surface areas - very low (HTC-1200, ~676 m²/g) and very high (HTC-750, ~2689 m²/g) - BET surface area correlates well with ion adsorption capacity. However, for the majority of carbons clustering in the intermediate range (1400-2000 m²/g), BET surface area shows no clear correlation with ion adsorption capacity, indicating that surface area alone is insufficient to predict electrochemically accessible porosity.*

We have addressed this in the main text:

"While there is some correlation between BET surface area and NMR-measured ion adsorption capacity for the HTC samples (Figure S7C), these quantities do not correlate well for commercial activated carbons. This suggests that BET surface area alone is insufficient to predict electrochemically accessible porosity, underscoring the value of the NMR approach over gas sorption-based methods."

2. The manuscript lacks detailed electrochemical characterization. Cyclic voltammetry (CV) at varying scan rates, electrochemical impedance spectroscopy (EIS), and analysis of ion diffusion kinetics should be included to evaluate performance comprehensively.

We emphasise that our electrochemical characterisation approach is comprehensive for the research objectives of this study. The multi-rate galvanostatic testing across current densities from 0.05 to 2 A/g (Figure S2) provides equivalent kinetic information to variable scan rate cyclic voltammetry, capturing the relevant performance characteristics across nearly two orders of magnitude in current density.

As described in our original Supporting Information Figure S2 (**A**) and (**B**)

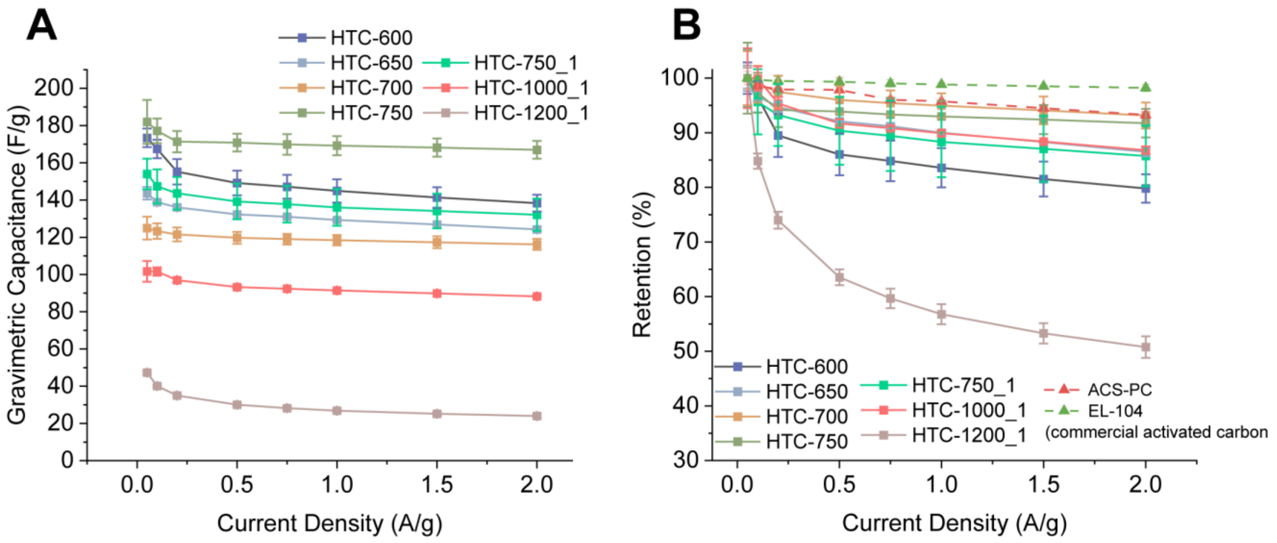

Figure S2: Gravimetric capacitances at different current densities and cyclability of the synthesised carbons. (**A**) Gravimetric capacitances of the synthesised nanoporous carbons in 1 M NEt_4BF_4 /ACN at different current densities. (**B**) Capacitance retention ratio of the synthesised nanoporous carbons in 1 M NEt_4BF_4 /ACN at different current densities, with the retention ratio calculated by gravimetric capacitance at *x* current density divided by the gravimetric capacitance at 0.05 A g^{-1}.

Figure S2 demonstrates that the low-temperature synthesised nanoporous carbons maintain high capacitance performance at higher current densities up to 2 A/g.

At the reviewer's suggestion, we have conducted additional EIS experiments on the synthesised nanoporous carbons:

From revised Supporting Information Figure S1 (**E**),

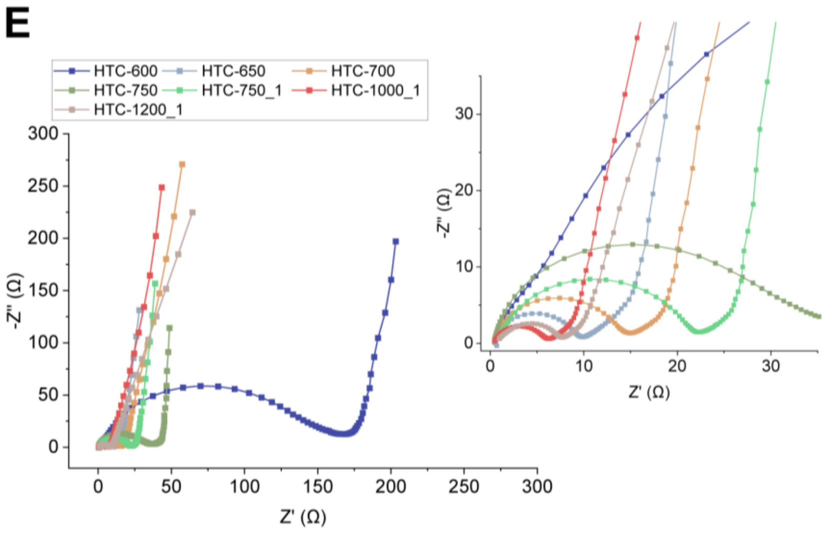

Figure S1: Galvanostatic charge−discharge plots and cyclic voltammograms of the synthesised carbons, with repeat measurements shown and EIS plots of the synthesised carbons. (**A**) Galvanostatic charge−discharge plots of the synthesised nanoporous carbons in 1 M NEt_4BF_4 /ACN

at 0.05 A g^{-1}. (**B**) Cyclic voltammograms of the synthesised nanoporous carbons at 10 mV s^{-1}. (**C**) Galvanostatic charge−discharge plots of the repeat cells of synthesised nanoporous carbons in 1 M NEt_4BF_4 /ACN at 0.05 A g^{-1}. (**D**) Cyclic voltammograms of the repeat cells of synthesised nanoporous carbons at 10 mV s^{-1}. (**E**) EIS measurements of the cells of synthesised nanoporous carbons in 1 M NEt_4BF_4 /ACN using the frequency ranging from 0.01 to 200k Hz. HTC-600 shows a large initial semicircle in the Nyquist plot, indicative of its low electrical conductivity.

We have addressed this in the main text:

"Firstly, carbons synthesised at lower temperatures exhibit slightly poorer conductivity (Tables S6 and S7), as indicated by the more resistive voltammogram for HTC-600, which deviated more evidently from the ideal rectangular shape (Figure S1), and by the large initial semicircle in its EIS Nyquist plot (Figure S1), both reflecting its low electrical conductivity."

This work focuses on establishing the fundamental thermodynamic relationship between structural parameters (*i.e.* graphene-like domain sizes and ion adsorption capacities) and capacitance and proving the concept that low-temperature synthesis is a promising path to engineer carbons with enhanced capacitance. Detailed kinetic analysis including electrolyte ion diffusion studies would be an interesting avenue for future work, although is ultimately beyond the scope of our current study.

For a detailed study on how the carbon structure influences the kinetic performance in supercapacitors, please see our recent work on tortuosity (https://chemrxiv.org/engage/chemrxiv/article-details/683f1bd91a8f9bdab5736850).

3. While the materials show good rate performance, it is unclear how low-temperature synthesis achieve this. Conductivity measurements should be provided.

Thank you for this suggestion. We note that direct conductivity measurements on nanoporous carbon powders are technically challenging. We have noted that low-temperature synthesised carbons exhibit lower conductivity, as evidenced by more resistive CV shapes for materials like HTC-600 (Figure S1). Despite this conductivity limitation, these materials maintain good rate performance, demonstrating that the capacitance gains overcome the resistive losses - a finding that validates the fundamental viability of our approach. For practical electrode applications, conductivity limitations can be addressed through the incorporation of conductive additives such as carbon black. Electrode composition optimisation represents important future work that extends beyond the scope of this fundamental proof-of-concept study.

At the reviewer's suggestion, we have included the cell resistance from constant charge-discharge measurements in Supporting Information Table S6:

Table S6: *Cell resistance of the synthesised HTC carbons and two commercial activated carbons (ACS-PC and EL-104) in 1 M NEt_4BF_4 /ACN. The cell resistance is read from the IR drop in constant charge-discharge measurements. HTC-600 shows evidently larger resistance, suggesting the potential lower electrical conductivity compared to other synthesised carbons.*

Carbon Name	Resistance of cell 1 (Ω)	Resistance of cell 2 (Ω)	Average (Ω)
HTC-600	150.5	158.20	154.35±5.44
HTC-650	18.36	23.80	21.08±3.85
HTC-700	17.48	25.60	21.54±5.74
HTC-750	61.70	67.04	64.37±3.78
HTC-750_1	36.51	22.72	29.62±9.75
HTC-1000_1	6.93	7.20	7.07±0.20
HTC-1200_1	12.90	15.20	14.05±1.63
ASC-PC	23.04	23.69	23.37±0.46
EL-104	29.50	36.00	32.75±4.60

Additionally, we have conducted conductivity measurements on the synthesised carbon films. From revised Supporting Information Table S7:

Table S7: *Conductivity from four-point probe measurements of the synthesised HTC carbon films and two commercial activated carbon films (ACS-PC and EL-104).*

Carbon Name	Conductivity (S/m)
HTC-650_6	0.004±0.001
HTC_700_6	0.012±0.008
HTC_750_6	0.326±0.077
HTC_750_1	0.213±0.022
HTC_1000_1	0.832±0.155
HTC_1200_1	0.256±0.106
ACS-PC	1.22±0.10
EL-104	0.47±0.07

In the main text:

"Firstly, carbons synthesised at lower temperatures exhibit slightly poorer conductivity (Tables S6 and S7), as indicated by the more resistive voltammogram for HTC-600, which deviated more evidently from the ideal rectangular shape (Figure S1), and by the large initial semicircle in its EIS Nyquist plot (Figure S1), both reflecting its low electrical conductivity."

While potential systematic errors in absolute conductivity values may arise from sample preparation and instrumentation, it is generally observed that the conductivity increases with the synthesis temperature, as expected.

4. The low-temperature synthesis and high density of edge sites may introduce side reactions. These potential reactions and their impacts on electrochemical stability should be investigated and discussed.

Thank you for this comment, **please also see our response to Reviewer 2, point 1**. We agree with the reviewer that low-temperature synthesised carbons have worse stability than commercial activated carbons. As we have discussed in the main text of the original manuscript:

"Secondly, the low-temperature synthesised carbons were found to have reduced cycling stability compared to commercial activated carbons (Figure S2). Floating tests were conducted to study the cycle lives (See Methods) of the low-temperature synthesised carbons and showed more rapid degradation compared to commercial activated carbons (Figure S2)."

At the reviewer's suggestion, we have addressed this in the main text:

"We hypothesise that the observed stability trade-offs may arise from two potential mechanisms[41]: (i) carbon edge sites acting as active catalytic sites that accelerate electrolyte decomposition reactions, or (ii) the disordered carbon structures themselves being inherently more reactive compared to more ordered carbons."

At the reviewer's suggestion, we have conducted preliminary XPS analysis on the degraded electrodes for some selected synthesised carbons:

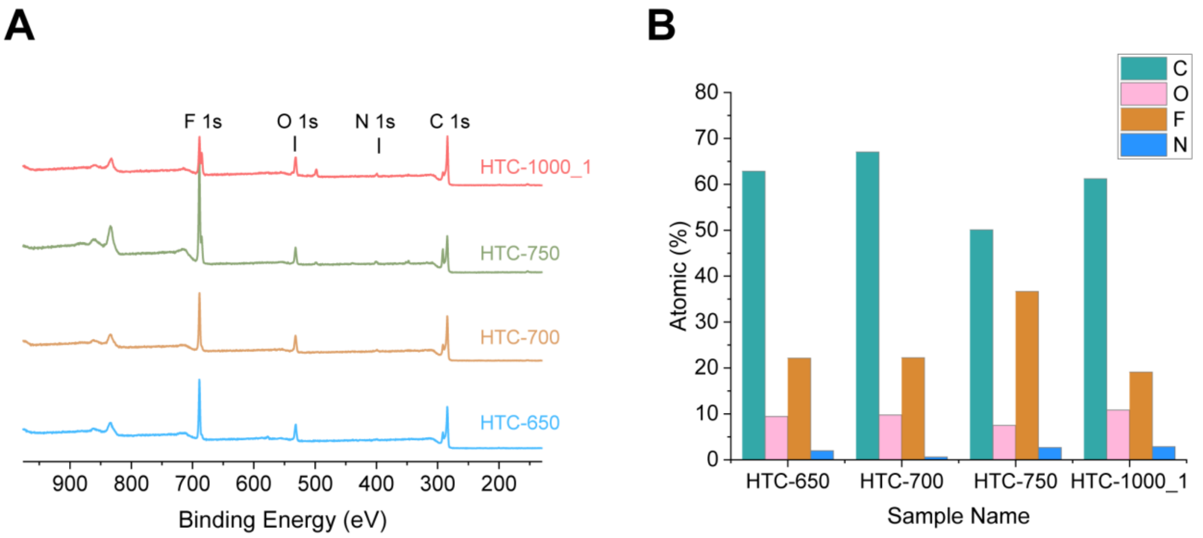

Figure R1. (**A**) XPS survey spectra and (**B**) elemental composition of the degraded synthesised nanoporous carbon films.

We acknowledge the importance of understanding degradation mechanisms in disordered carbons and the effects of carbon structure on degradation processes. Based on previous literature, carbon supercapacitor degradation mechanisms could involve electrolyte decomposition at carbon surface defects, pore blocking

by decomposition products, carbon surface oxidation, and electrode/electrolyte interphase degradation (*Adv. Energy Mater*. **2023**, 13, 2301008). While the carbon local structural order degree would play an important role in the degradation, there are multiple factors that could affect this process including the surface properties of the carbon electrodes, the electrode polarisation, trace amounts of water in the system, *etc* (*Energy Storage Mater*. **2015**, 209, 90-98). Here, the lack of clear correlation between our XPS results (**Figure R1**) and degradation rates indicates that surface chemical composition alone cannot predict the carbons' stability, highlighting the complexity of degradation mechanisms. For a more definitive understanding of the degradation mechanisms, comprehensive studies on the degradation processes including post-cycling electrode analysis and electrolyte analysis combined with multiple characterisation techniques are needed. While we acknowledge that the low-temperature synthesised carbons show worse stability as a potential limitation, this does not compromise the fundamental scientific contributions of the current study. The observed stability challenges represent important engineering targets for future optimisation.

5. In Figure S1, charge–discharge and CV tests were conducted over different voltage ranges. The rationale for using different potential windows should be clearly explained.

We used the same voltage ranges for constant charge-discharge and CV measurements. We have revised Figure S1 for a clearer demonstration.

From the revised Supporting Information:

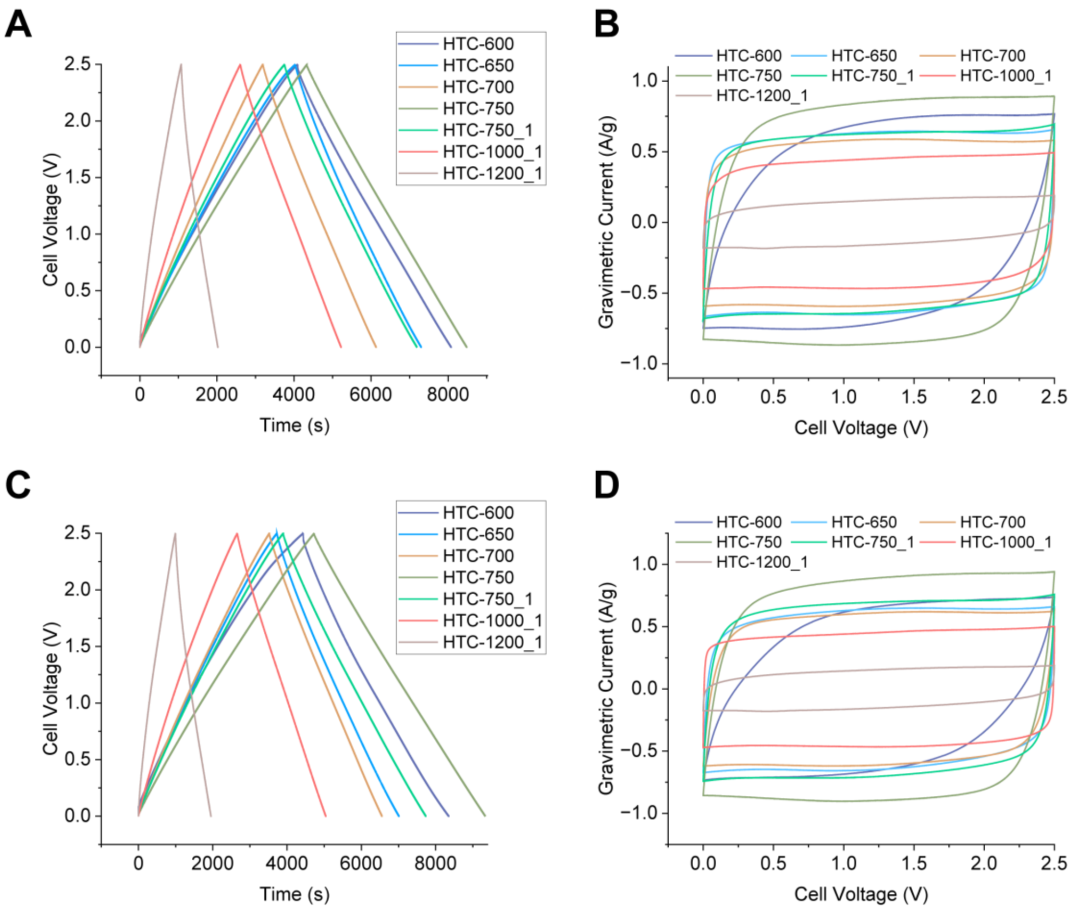

Figure S1: Galvanostatic charge−discharge plots and cyclic voltammograms of the synthesised carbons, with repeat measurements shown. (**A**) Galvanostatic charge−discharge plots of the synthesised nanoporous carbons in 1 M NEt_4BF_4 /ACN at 0.05 A g^{-1}. (**B**) Cyclic voltammograms of the synthesised nanoporous carbons at 10 mV s^{-1}. (**C**) Galvanostatic charge−discharge plots of the repeat cells of synthesised nanoporous carbons in 1 M NEt_4BF_4 /ACN at 0.05 A g^{-1}. (**D**) Cyclic voltammograms of the repeat cells of synthesised nanoporous carbons at 10 mV s^{-1}.

6. The manuscript should include a more in-depth discussion of the formation mechanism responsible for generating the small graphene-like domains under low-temperature conditions.

The formation of small graphene-like domains under low-temperature conditions is fundamentally governed by kinetic limitations that prevent extensive graphitisation processes. At lower activation temperatures (600-750°C), the thermal energy is insufficient to drive the coalescence and ordering of carbon atoms into large, well-ordered graphitic structures that occur during high-temperature graphitisation (>800°C). Instead, the carbon framework undergoes limited structural reorganisation, preserving the disordered nature of the hydrothermal carbon precursor while developing porosity through KOH activation. This contrasts with conventional high-temperature activation. The temperature-dependent evolution of carbon domain sizes has been extensively studied in previous literatures (*Adv. Mater.*,**2006**, (*18*), 2073-2094; *Carbon*, **2016**, (*109*), 681-693), as also noted by Reviewer 4.

We have addressed this in the main text:

"The observed temperature-dependent evolution of domain sizes aligns with previous findings,[4,32,35] where carbons synthesised at lower temperatures have smaller graphene-like domains due to kinetic limitations that hinder extensive graphitisation."

7. The SSA increases sharply from HTC-700 to HTC-750. This sudden change requires further discussion and a clearer explanation.

During KOH activation, KOH initially reacts with carbon to form K_2CO_3, which subsequently reacts with additional carbon and decomposes to produce K_2O, CO, and CO_2. Therefore, the pores are formed based on three potential mechanisms: i) etching of the carbon framework by redox reactions, ii) physical activation by gaseous products, *e.g.* CO and CO_2 and iii) the intercalation of metallic potassium, which further expands the carbon lattices. At temperatures higher than 700°C, K_2CO_3 begins to decompose into K_2O and CO_2, resulting in dramatic pore opening and substantially increased BET surface areas.

As we have discussed in the main text of the original manuscript,

"During the KOH activation process, KOH decomposes into K_2CO_3 around 400°C, which further breaks down into K_2O and CO_2 at temperatures above 700°C, leading to activated carbons with high BET surface areas and development of pores around 2 nm diameter.[17,18,20]"

"We attribute this to the significant pore opening at 750 °C which gives rise to the sample with the highest BET surface area (2689 m^2 g^{-1}) in the series and a significant number of pores below 1 nm in diameter and around 2 nm in diameter (Figure 1b, Table S1). This is likely due to the more dramatic etching of the carbon framework at 750 °C by KOH and the formation of potassium-containing compounds at this temperature (i.e. K_2CO_3 and K_2O).[17,18]"

This phenomenon is also extensively reported in previous literature (*Journal of Materials Chemistry*, **2012**, (*22*), 23710-23725; *Advanced Energy Materials*, **2011**, (*1*), 356-361)

8. Although the overall oxygen content appears similar among the samples, the distribution and types of oxygen-containing functional groups likely differ. These differences should be analyzed and discussed in relation to performance. In addition, the XPS results in Table S2 show that HTC-650 has a significantly higher oxygen content than the other samples. This anomaly should be verified, and the variations in oxygen species across all samples should be interpreted and explained.

We have deconvoluted the types of oxygen-containing functional groups in XPS measurements. As shown in our original Supporting Information Figure S4:

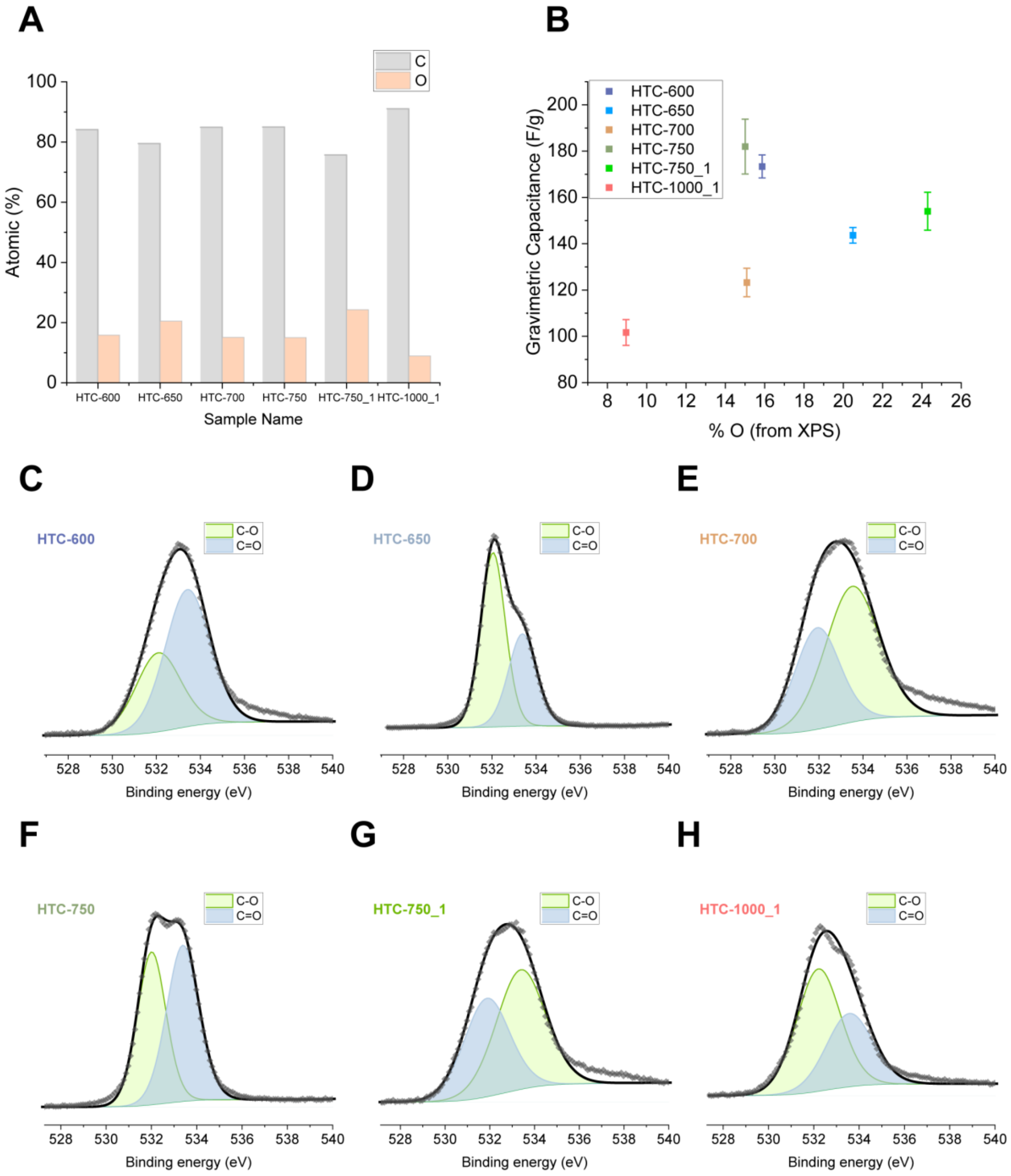

Figure S4: X-ray photoelectron spectroscopy (XPS) results of the synthesised carbons. (A) XPS survey of the synthesised nanoporous carbons. **(B)** Relationship between gravimetric capacitances in 1 M NEt_4BF_4 /ACN and %O (atomic percent) measured from XPS for the synthesised carbons, with no clear correlation observed, similar to our previous study.[2] Fitted O1s core-level spectra of **(C)** HTC-600, **(D)** HTC-650, **(E)** HTC-700, **(F)** HTC-750, **(G)** HTC-750_1 and **(H)** HTC-1000_1.

At the reviewer's suggestion, we have also now included the results in the Supporting Information Table S2.

Table S2: %C and %O of part of the studied carbons, measured from X-ray photoelectron spectroscopy (XPS). The percentage represents atomic percent (at%).

Carbon	%C	%O	O-C (at%)	O=C (at%)
HTC-600	84.13	15.87	8.96	5.16
HTC-650	79.51	20.49	9.27	15.1
HTC-700	84.91	15.09	6.82	4.25
HTC-750	84.99	15.01	7.8	6.7
HTC-750_1	75.71	24.29	4.31	3.28
HTC-1000_1	91.05	8.95	4.25	6.72

At the reviewer's suggestion, we have additionally included the relationship between capacitance and different types of oxygen-containing functional groups in Supporting Information Figure S4.

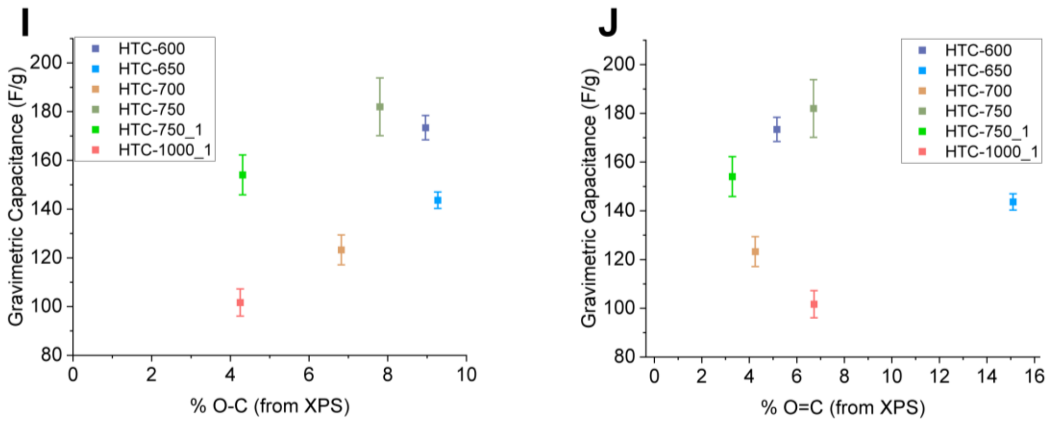

Figure S4: X-ray photoelectron spectroscopy (XPS) results of the synthesised carbons. (A) XPS survey of the synthesised nanoporous carbons. **(B)** Relationship between gravimetric capacitances in 1 M NEt_4BF_4 /ACN and %O (atomic percent) measured from XPS for the synthesised carbons, with no clear correlation observed, similar to our previous study.[2] Fitted O1s core-level spectra of **(C)** HTC-600, **(D)** HTC-650, **(E)** HTC-700, **(F)** HTC-750, **(G)** HTC-750_1 and **(H)** HTC-1000_1. **(I)** Relationship between gravimetric capacitances in 1 M NEt_4BF_4 /ACN and %O-C (atomic percent) type functional groups measured from XPS for the synthesised carbons. **(J)** Relationship between gravimetric capacitances in 1 M NEt_4BF_4 /ACN and %O=C (atomic percent) type functional groups measured from XPS for the synthesised carbons.

It is worth noting that there is no obvious correlation between gravimetric capacitance in 1 M NEt_4BF_4 /ACN and different types of oxygen-containing functional groups. As described in the main text of the original manuscript:

"Additional characterisation using X-ray photoelectron spectroscopy (XPS) measurements revealed high surface oxygen content (~15-20 at. %) for all synthesised carbons (Figure S4 and Table S2), though the oxygen content did not show any apparent correlation with gravimetric capacitance (Figure S4), similar to our previous findings.[21] We also note that the oxygen content in bulk is much lower (less than 10 wt%) (Table S3). While oxygen-containing functional groups can affect the point of zero charge (PZC) of the nanoporous carbons and thus influence their performance in aqueous electrolytes,[10] these effects may be less significant in the organic electrolyte (1 M NEt_4BF_4 /ACN) used in the current study.[38]"

HTC-750_1 exhibits the highest surface oxygen content among all carbons from XPS measurements, which can be attributed to the most dramatic activation at this temperature, as previously discussed. HTC-650 shows approximately 5 at% higher oxygen content than HTC-750 and other carbons synthesised with six-hour activation times. This anomaly can potentially be explained by the competing effects of activation intensity and duration: while 750 °C leads to the most dramatic activation, the six-hour treatment at this temperature leads to burn-off of oxygen-containing functional groups, whereas the lower temperature (650°C) preserves these groups during the extended activation period.

Response to Reviewer 4

This work investigated the electrical double-layer capacitance (EDLC) of nanoporous carbon materials, specifically, using NMR, from which, the authors tried to extend conclusions, including: 1) low-temperature synthesis is a promising pathway to produce nanoporous carbons with enhanced capacitance in EDLC; 2) Nanoporous carbons produced at low temperature have a smaller graphene-like domain; 3) Carbon with smaller graphene-like domains and higher ion adsorption capacity show enhanced EDLC; and 4) Δδ and the ion adsorption capacity both measured by NMR can be used as descriptors for capacitance. Among the above four points of conclusion, only the fourth one can mark this work a valuable investigation, while the other three are either worthless or unacceptable.

We thank the reviewer for their comments. All revisions made in the manuscript are highlighted in yellow for clarity. Our detailed responses to each comment are provided below.

Conclusion item 1: Carbon materials produced at "low temperature" do not always render enhanced EDLC. Figures 1, 3, and 3 showed that HTC-750 outperformed HTC-600 in gravimetric capacitance. If the authors lower the heat treatment temperature down to 300 oC, and name the sample HTC-300. One can expect an even lower gravimetric capacity for the HTC-300 than for the HTC-600, which is likely due to the HTC-300's lower carbonization level. Therefore, conclusion item 1 is misleading to readers, particularly those unfamiliar with this topic.

At the reviewer's suggestion, we have specified the temperature range in the main text:

"We emphasise that "low-temperature synthesis" refers specifically to the effective KOH activation temperature range of 600-750°C, below which insufficient carbonisation may lead to decreased material performance."

Conclusion item 2: Carbon materials, including nanoporous carbon, produced at a lower temperature always have a smaller graphene-like domain if the other synthesis conditions are kept identical. A larger graphene-like domain indicates a higher level of graphitization; graphitization is an endothermic reaction process, which requires a higher temperature or extended heat treatment time. Low-temperature heating treatment yields a lower graphitization level of pyrolytic carbon produced from the same precursor. This is widely accepted. Conclusion item 2 contributes nothing new to the knowledge.

We appreciate that it is widely accepted as we expected. Our study does not emphasise that low-temperature synthesis leading to smaller graphene-like domains is a novel finding.

While our previous Science paper (*Science* **2024**) established the fundamental principle that structural disorder drives capacitance using commercial carbons, the current work represents a major advancement in three critical areas:

1. **Rational Synthesis Strategy:** We now provide a systematic synthetic route to produce highly disordered carbons, moving from discovery to practical implementation. This bridges the gap between fundamental understanding and materials engineering.

2. **Dual-Factor Performance Model:** Our previous work discovered that domain sizes dominate capacitance in nanoporous carbons. With a wider range of carbons, here we reveal that both graphene-like domain sizes and ion adsorption capacities determine capacitance, providing a more

complete mechanistic understanding, which has not been reported in previous literature and extends the conclusion in our previous study.

3. **Data-Driven Prediction:** We establish the first quantitative method to predict capacitance directly from NMR spectra, transforming NMR from a characterisation tool into a materials screening platform. This enables rapid identification of high-performance materials without electrochemical testing.

As described in our original manuscript:

In abstract: *"NMR spectroscopy measurements provided unique insights by simultaneously probing local structural order and ion adsorption capacities, revealing that carbons with smaller graphene-like domain sizes and higher ion adsorption capacities generally have better capacitive performance."*

In the main text: *"Importantly, in addition to providing a measurement of the sizes of the graphene-like domains, our NMR experiments measure another key property of the nanoporous carbons; the electrolyte ion adsorption capacity. Indeed, the integrals of the in-pore resonances relate to the number of ions which can access the carbon nanopores in the absence of applied potential. Deconvolution of the NMR spectra of the electrolyte-saturated carbon samples shows that the gravimetric capacitance showed some correlation with the ion adsorption capacity (Figure 1f), with HTC-750 having the largest ion adsorption capacity in the series (1.6 mmol g^{-1})."*

In conclusion: *"NMR spectroscopy, in particular, allowed us to simultaneously evaluate both the sizes of the graphene-like domains and the electrolyte ion adsorption capacities, with electrolyte ions as the probe. Our findings indicate that carbons with smaller graphene-like domains and higher ion adsorption capacity generally show enhanced capacitive performance. The highest gravimetric capacitances were achieved for either highly disordered carbons or carbons with more order but more accessible pores (higher electrolyte ion adsorption capacities), the latter carbons, however, generally being associated with lower volumetric capacitances. We show that the combination of $\Delta\delta$ and the ion adsorption capacity both measured by NMR spectroscopy in a quick and simple NMR experiment (~5 mins) are excellent descriptors for capacitance, enabling rapid identification of high-performance materials without electrochemical testing"*

At the reviewer's suggestion, we have addressed this in the main text and conclusion:

"With an expanded series of nanoporous carbons including those from our previous studies, it is observed that carbons with higher gravimetric capacitances generally have (i) larger ion adsorption capacities (Figure 3a) and (ii) $\Delta\delta$ values of smaller magnitude (Figure 3b), which identifies ion adsorption capacity as another key descriptor for capacitance and extends our previous findings.[21]"

"We show that the combination of $\Delta\delta$ and the ion adsorption capacity both measured by NMR spectroscopy in a quick and simple NMR experiment (~5 mins) are excellent descriptors for capacitance, enabling rapid identification of high-performance materials without electrochemical testing."

Conclusion item 3: It is questionable that carbon with smaller graphene-like domains shows enhanced EDLC. The pore size relevant to the ion size is a critical factor that dominates the EDLC. Figure 1b shows that HTC-750 is rich in subnanometer pores compared to HTC-700, -650, and -600. Figure 1e, 1f, 2a, and 2b show that HTC-750 has the highest gravimetric capacity. Considering that the size of TEA ion is approximately 0.45 nm, the subnanopore in HTC-750 should be a key factor that yields the best gravimetric capacity. The critical role of pore size on the EDLC was first reported by Prof. Simmon and Gogotsi (Science 2006), and this phenomenon has been reported in the following research by

different research groups. Unfortunately, one can barely find the discussion on the effect of pore size in the manuscript, even the HTC-750 showed clearly a different pore size distribution (Fig 1b).

HTC-750 exhibits more subnanopores and also more wider pores around and above 2 nm, as shown in the original Supporting Information Figure S3 **(B)**

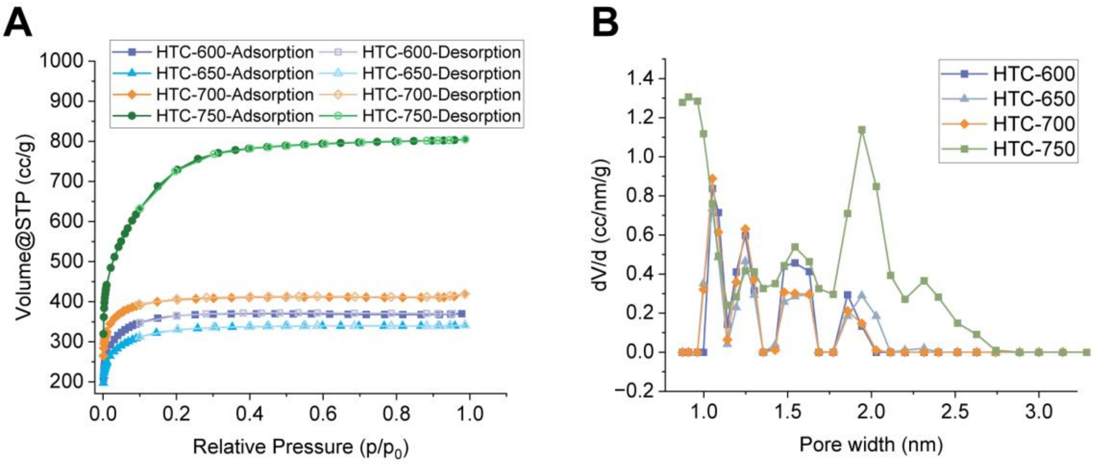

Figure S3: Gas sorption isotherms and pore size distribution plots of the synthesised carbons. (**A**) N_2 isotherms at 77 K of the four synthesised nanoporous carbons (filled dots show adsorption, hollow dots show desorption). **(B)** Pore size distributions based on 2D-NLDFT analysis (slit pore model) of N_2 isotherms at 77 K in **(A)**.[1]

While we acknowledge that pore-size theory is accepted by many researchers in the supercapacitor field, our recent study of 20 nanoporous carbons showed no obvious correlation between capacitance and average pore size (*Science*, **2024**, (*384*), 321-325). The key study mentioned by the reviewer (Science 2006) studied carbons where *both* pore size and structural disorder varied *i.e. that study could not isolate the impact of pore size alone on capacitance*, and our recent study suggests that disorder effects, rather than pore size, caused the reported variations in capacitance on the 2006 paper (*Science*, **2024**, (*384*), 321-325). We also note that the lack of correlation between carbon pore size and capacitance has also been reported by other researchers (*Phys. Chem. Chem. Phys.* **2011** (*13*) 12403-12406).

At the reviewer's suggestion, and for completeness, we have included the relationship between gravimetric capacitance and average pore size in the Supporting Information Figure S3. This data again shows results contrary to the results of the 2006 Science paper.

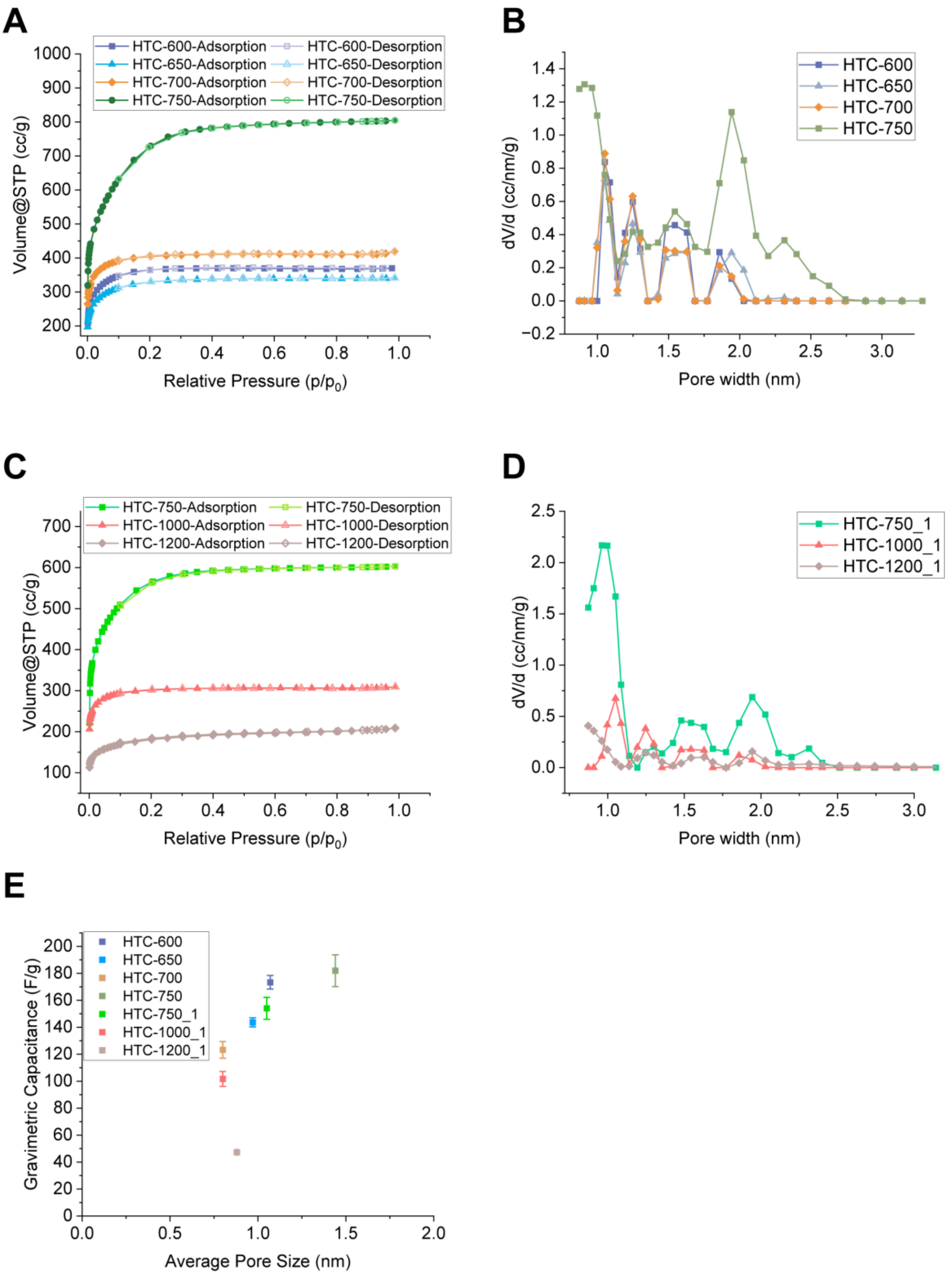

Figure S3: Gas sorption isotherms and pore size distribution plots of the synthesised carbons. (**A**) N_2 isotherms at 77 K of the four synthesised nanoporous carbons (filled dots show adsorption, hollow dots show desorption). **(B)** Pore size distributions based on 2D-NLDFT analysis (slit pore model) of N_2 isotherms at 77 K in **(A)**.[1] (**C**) N_2 isotherms at 77 K of three synthesised nanoporous carbons (filled dots show adsorption, hollow dots show desorption). **(D)** Pore size distributions based on 2D-NLDFT analysis

(slit pore model) of N_2 isotherms at 77 K in **(C)**.[1] **(E)** Relationship between gravimetric capacitances of the synthesised nanoporous carbons in 1 M NEt_4BF_4 /ACN and average pore size.

Additionally, the activated carbons studied here exhibit broad pore size distributions (Figure S3) where average pore size is not a representative parameter, unlike carbide-derived carbons (CDCs) which typically have narrower distributions but were not the focus of this study.

Conclusion item 4: This is the most valuable conclusion. Insightful and practically beneficial. However, this conclusion still requires a more comprehensive study. For example, applying carbon materials from a larger variety of sources to present the viability of this method, and illustrate the physical picture at the microscale to allow readers who are not experts in NMR to understand its fundamentals easily.

We thank the reviewer for their positive comments. We feel that the study is comprehensive in demonstrating the use of Δδ and the ion adsorption capacity (both measured by NMR) as descriptors for capacitance. We emphasise that our NMR-based capacitance prediction successfully encompasses commercial carbons from different precursors and synthesis routes, including activated carbon cloths (ACC series), coconut shell-derived carbons (YP series), glucose-derived carbons (HTC series) and other carbons with fundamentally different 3D structures, demonstration the universality of our prediction method for predominantly microporous carbons. In future studies we will be expanding this approach to carbons prepared via a wider range of synthesis routes.

Response to Reviewer 5

The article "Highly disordered nanoporous carbons for enhanced energy storage in supercapacitors" reports a study on KOH-activated carbons used as electrodes and tested with an organic electrolyte for supercapacitor performance. Authors correlate the electrochemical test parameters with NMR, Raman spectroscopy and nitrogen gas sorption data. While the findings are interesting and bring important factor (temperature) for carbon preparation again to discussion, this work considerably lacks novelty.

We thank the reviewer for their comments. Following our previous Science paper (*Science* **2024**, https://www.science.org/doi/10.1126/science.adn6242), which established the fundamental principle that structural disorder drives capacitance using commercial carbons, this work represents a major advancement in three critical areas:

1. **Rational Synthesis Strategy:** We now provide a systematic synthetic route by varying the activation temperature to produce highly disordered carbons, moving from discovery to practical implementation. This bridges the gap between fundamental understanding and materials engineering.

2. **Dual-Factor Performance Model:** Our previous work discovered that domain sizes dominate capacitance in nanoporous carbons. With a wider range of carbons, here we reveal that both graphene-like domain sizes and ion adsorption capacities determine capacitance, providing a more complete mechanistic understanding, which has not been reported in previous literature and extends the conclusion in our previous study.

3. **Data-Driven Prediction:** We establish the first quantitative method to predict capacitance directly from NMR spectra, transforming NMR from a characterisation tool into a materials screening platform. This enables rapid identification of high-performance materials without electrochemical testing.

Authors use a relatively low temperature treatment of organic precursors with KOH to produce activated carbons with disorder. Authors state that they do not find a correlation between pore size and capacitance values of the carbon materials, contrary to previously well-established concept. As a matter of fact, activated carbons, both powder and cloth are very sensitive material due to the edge sites and dangling bonds rendering them vulnerable to fast reaction with oxygen and moisture from the atmosphere. This causes the generation of oxygenated functional groups on the surface and of course block the pores entrances. The degree of functionalization depends on how long the carbon sample was kept in open or closed environment. It is therefore, necessary to perform heat treatment ~700 ° C before using any carbon material in a supercapacitor device (especially with non-aqueous electrolyte). If ignored, the pore blockage of non-treated carbon could lead to significant capacitance discrepancies in the obtained electrochemical performance.

Some of the works on surface functional groups are listed below.

https://pubs.acs.org/doi/10.1021/ie061071v Characterization of Active Sites on Carbon Catalysts |

Industrial & Engineering Chemistry Research

https://www.sciencedirect.com/science/article/abs/pii/S0008622398003339 Modification of the surface chemistry of activated carbons - ScienceDirect

https://www.sciencedirect.com/science/article/abs/pii/S0008622308006696 Effect of electrochemical treatments on the surface chemistry of activated carbon - ScienceDirect

https://www.sciencedirect.com/science/article/pii/S0957582021000707 Feasibility of electrochemical regeneration of activated carbon used in drinking water treatment plant. Reactor configuration design at a pilot scale - ScienceDirect

https://link.springer.com/article/10.1007/s10800-013-0644-0 Factors contributing to ageing of high voltage carbon/carbon supercapacitors in salt aqueous electrolyte | Journal of Applied Electrochemistry

https://www.sciencedirect.com/science/article/abs/pii/S0013468620308082 Fitting the porous texture of carbon electrodes to a binary ionic liquid electrolyte for the realization of low temperature EDLCs – ScienceDirect

Specifically, our NMR approach directly measures the ion adsorption capacity within the nanopores, which is a more direct measurement of the ion accessible porosity that is directly responsible for the capacity than the gas physisorption technique. As we have now addressed in the revised manuscript:

In Introduction: "*Additionally, the integral of the in-pore resonance quantifies the ion adsorption capacity within nanopores in the absence of applied potential. This approach provides a more direct measure of ion accessible porosity than gas physisorption analysis, as the latter relies on the assumption that pores accessible to small gas molecules at measurement temperatures (e.g., 77 K for N_2) are equally accessible to the larger electrolyte ions at room temperature.*"

Importantly, we have shown that the ion adsorption capacity (as measured by NMR), together with the graphene-like domain sizes, determines the capacitance in nanoporous carbons.

As described in our original manuscript:

In abstract: *"NMR spectroscopy measurements provided unique insights by simultaneously probing local structural order and ion adsorption capacities, revealing that carbons with smaller graphene-like domain sizes and higher ion adsorption capacities generally have better capacitive performance."*

In the main text: *"Importantly, in addition to providing a measurement of the sizes of the graphene-like domains, our NMR experiments measure another key property of the nanoporous carbons; the electrolyte ion adsorption capacity. Indeed, the integrals of the in-pore resonances relate to the number of ions that can access the carbon nanopores in the absence of applied potential. Deconvolution of the NMR spectra of the electrolyte-saturated carbon samples shows that the gravimetric capacitance showed some correlation with the ion adsorption capacity (Figure 1f), with HTC-750 having the largest ion adsorption capacity in the series (1.6 mmol g^{-1})."*

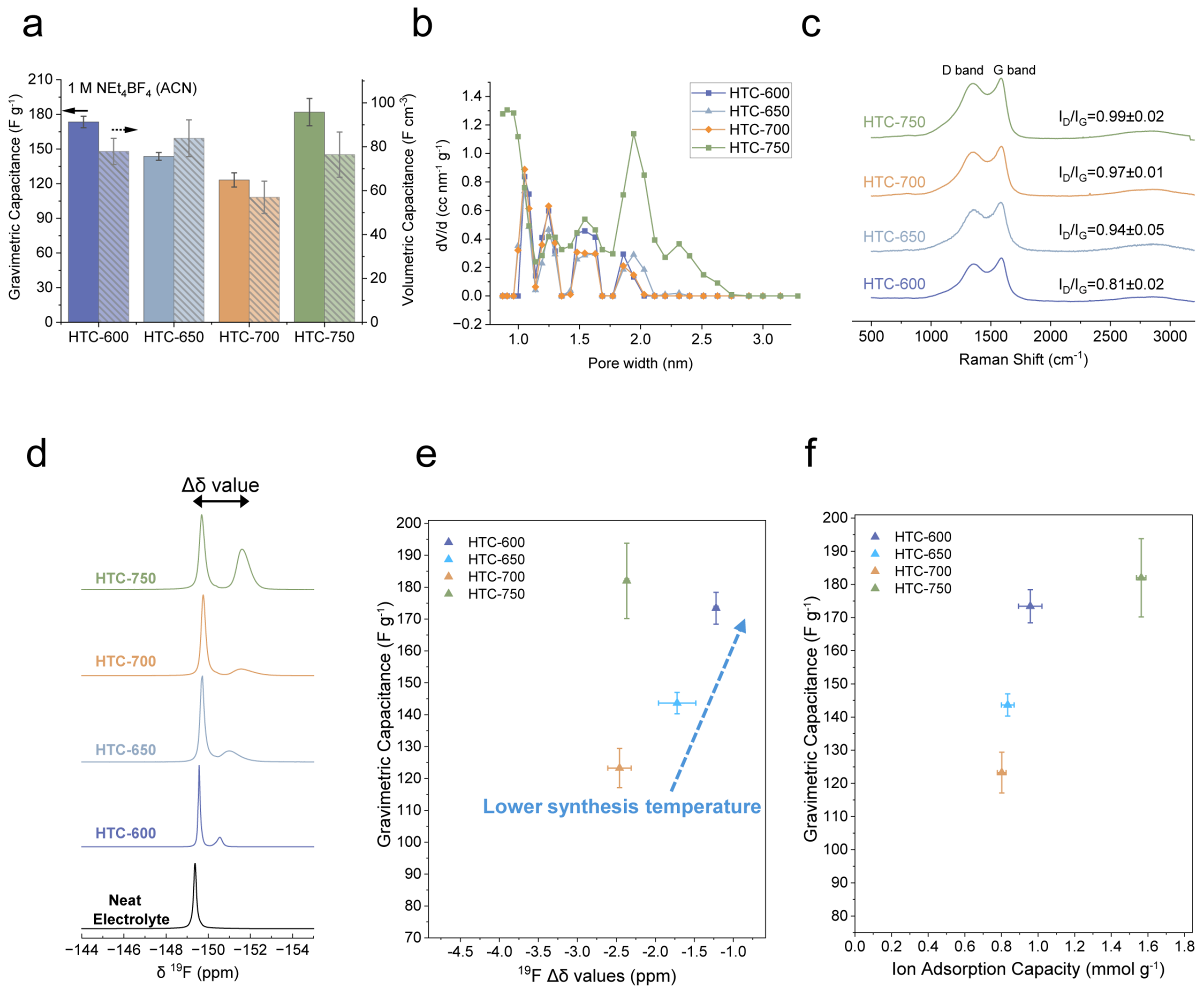

Figure 1. (a) Gravimetric and volumetric capacitances of low-temperature synthesised carbons measured at 0.05 A g^{-1} in 1 M NEt_4BF_4 /ACN, with the masses of the electrodes shown in Table S4. (b) Pore size distributions of low-temperature synthesised carbons calculated based on 2D non-local density functional theory (2D-NLDFT)[39] analysis (slit pore model) of N_2 isotherms at 77 K, with BET surface area, pore volume and average pore size presented in Table S1. (c) Raman spectra of low-temperature synthesised carbons (532 nm). See Supporting Information and our previous work[32] for details on deconvolution of Raman spectra. (d) ^{19}F MAS NMR spectra (14.1 T, 5 kHz MAS) of the synthesised carbons soaked with 1 M NEt_4BF_4 /ACN electrolyte. (e) Plot of gravimetric capacitance and ^{19}F $\Delta\delta$ values derived from d), with the in-pore chemical shifts taken as the weighted average for carbons showing multiple in-pore environments. (f) Relationship between gravimetric capacitance at 0.05 A g^{-1} in 1 M NEt_4BF_4 /ACN and ion adsorption capacity.

"With an expanded series of nanoporous carbons including those from our previous studies, it is observed that carbons with higher gravimetric capacitances generally have (i) larger ion adsorption capacities (Figure 3a) and (ii) $\Delta\delta$ values of smaller magnitude (Figure 3b), which identifies ion adsorption capacity as another key descriptor for capacitance and extends our previous findings.[21]"

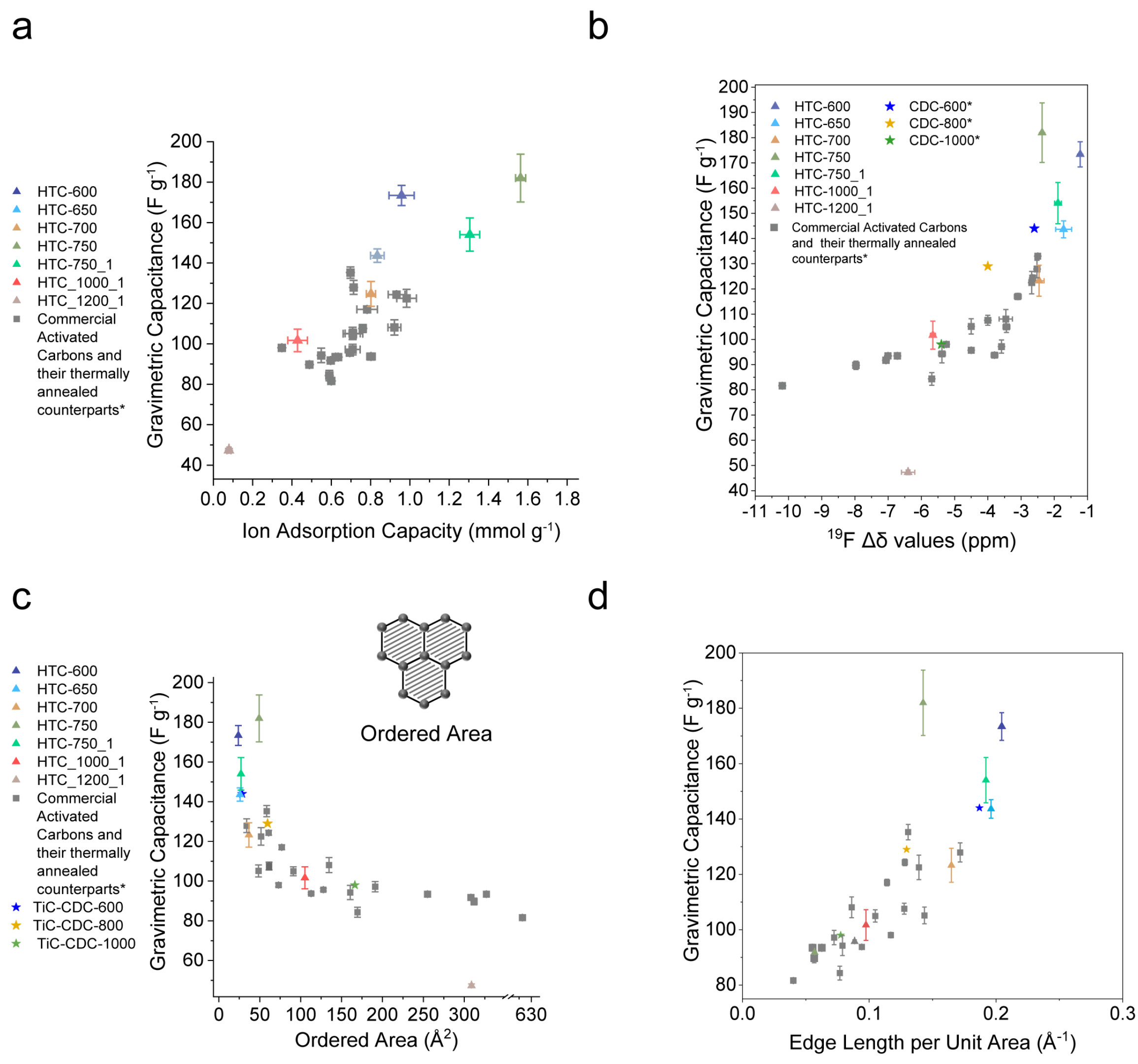

Figure 3. (a) Correlation between gravimetric capacitance and ion adsorption capacities, with commercial activated carbons, their thermally annealed counterparts data added from previous studies.[3,21] *See Figure S7 for the relationship between ion adsorption capacities and BET surface areas. (b) Correlation between gravimetric capacitance and* ^{19}F *Δδ values, with commercial activated carbons, their thermally annealed counterparts and CDC data added from previous studies.*[3,21] *(c) Relationship between gravimetric capacitance and calculated ordered domain size of the synthesised nanoporous carbons, with commercial activated carbons, their thermally annealed counterparts and CDC data added from previous studies.*[21] *(d) Correlation between gravimetric capacitance and edge length per unit area for synthesised carbons, with commercial activated carbons and their thermally annealed counterparts data added from previous studies.*[21] *See Methods for detailed definitions of the edge length per unit area.*

In conclusion: *"NMR spectroscopy, in particular, allowed us to simultaneously evaluate both the sizes of the graphene-like domains and the electrolyte ion adsorption capacities, with electrolyte ions as the probe. Our findings indicate that carbons with smaller graphene-like domains and higher ion adsorption capacity generally show enhanced capacitive performance. The highest gravimetric capacitances were achieved for either highly disordered carbons or carbons with more*

order but more accessible pores (higher electrolyte ion adsorption capacities), the latter carbons, however, generally being associated with lower volumetric capacitances. We show that the combination of Δδ and the ion adsorption capacity both measured by NMR spectroscopy in a quick and simple NMR experiment (~5 mins) are excellent descriptors for capacitance, enabling rapid identification of high-performance materials without electrochemical testing"

We acknowledge that functional groups potentially influence the capacitance of nanoporous carbons. We have extensively studied the oxygen-containing functional groups using XPS measurements. As shown in our original Supporting Information Figure S4:

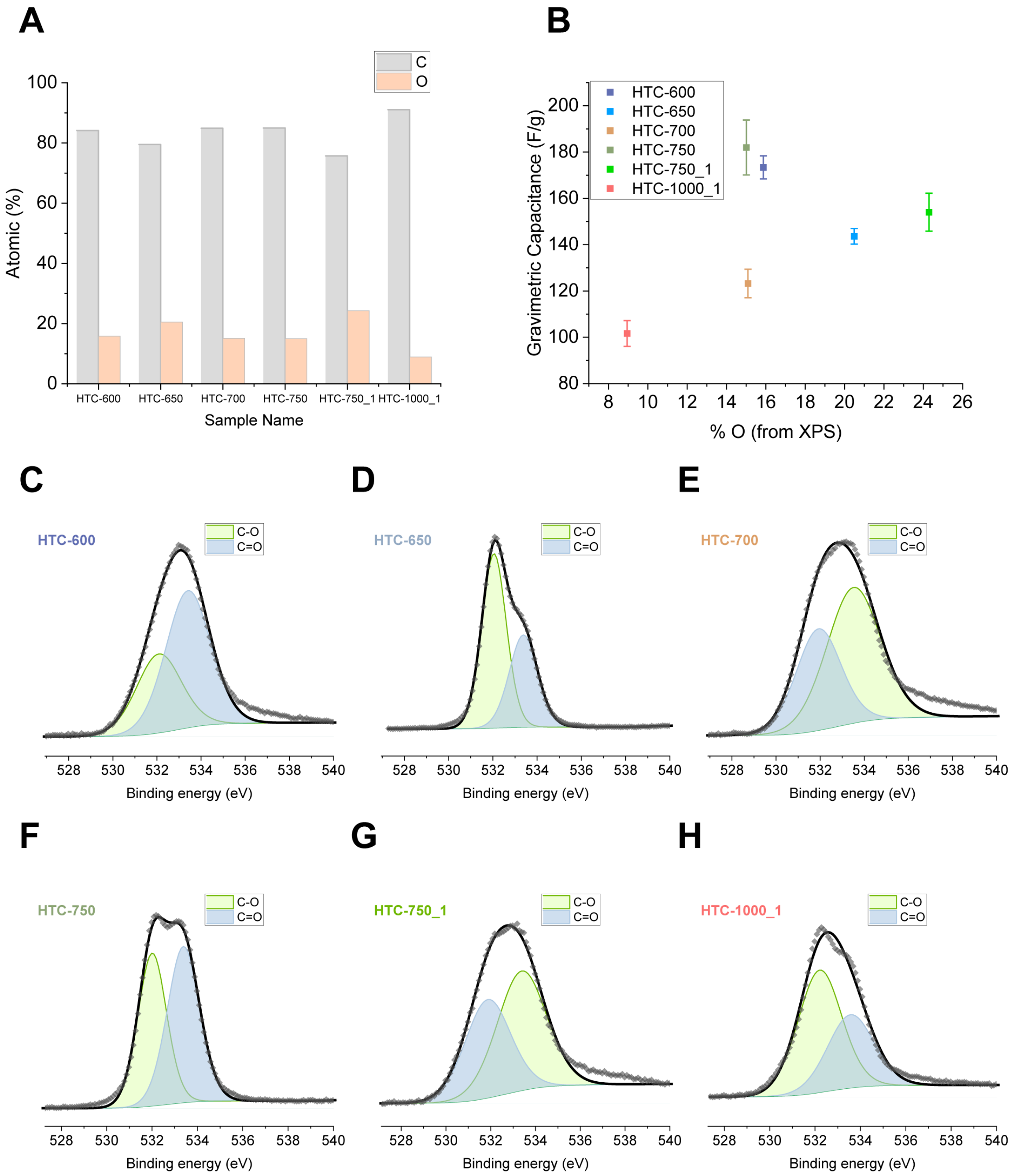

Figure S4: X-ray photoelectron spectroscopy (XPS) results of the synthesised carbons. (A) *XPS survey of the synthesised nanoporous carbons.* ***(B)*** *Relationship between gravimetric capacitances in 1 M NEt_4BF_4 /ACN and %O (atomic percent) measured from XPS for the synthesised carbons, with no clear correlation observed, similar to our previous study.[2] Fitted O1s core-level spectra of* ***(C)*** *HTC-600,* ***(D)*** *HTC-650,* ***(E)*** *HTC-700,* ***(F)*** *HTC-750,* ***(G)*** *HTC-750_1 and* ***(H)*** *HTC-1000_1.*

We have also included the results in the Supporting Information **Table S2**.

Table S2: *%C and %O of part of the studied carbons, measured from X-ray photoelectron spectroscopy (XPS). The percentage represents atomic percent (at%).*

Carbon	%C	%O	O-C (at%)	O=C (at%)
HTC-600	84.13	15.87	8.96	5.16
HTC-650	79.51	20.49	9.27	15.1
HTC-700	84.91	15.09	6.82	4.25
HTC-750	84.99	15.01	7.8	6.7
HTC-750_1	75.71	24.29	4.31	3.28
HTC-1000_1	91.05	8.95	4.25	6.72

Additionally, we have included the relationship between capacitance and different types of oxygen-containing functional groups in Supporting Information **Figure S4**.

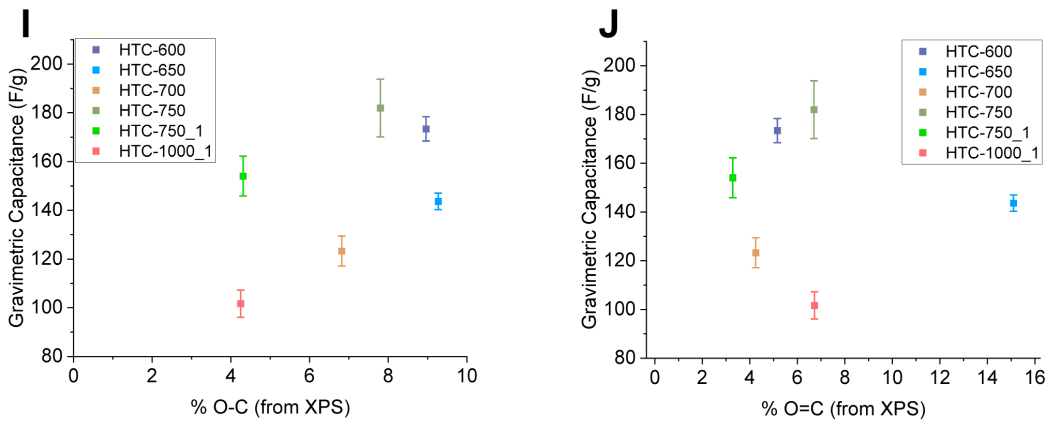

Figure S4: X-ray photoelectron spectroscopy (XPS) results of the synthesised carbons. (I) *Relationship between gravimetric capacitances in 1 M NEt_4BF_4 /ACN and %O-C (atomic percent) type functional groups measured from XPS for the synthesised carbons.* ***(J)*** *Relationship between gravimetric capacitances in 1 M NEt_4BF_4 /ACN and %O=C (atomic percent) type functional groups measured from XPS for the synthesised carbons.*

It is worth noting that there is no obvious correlation between gravimetric capacitance in 1 M NEt_4BF_4 /ACN and different types of oxygen-containing functional groups. As described in the main text of the original manuscript:

"Additional characterisation using X-ray photoelectron spectroscopy (XPS) measurements revealed high surface oxygen content (~15-20 at. %) for all synthesised carbons (Figure S4 and Table S2), though the oxygen content did not show any apparent correlation with gravimetric capacitance (Figure S4), similar to our previous findings.[21] We also note that the oxygen content in the bulk is much lower (less than 10 wt%) (Table S3). While oxygen-containing functional groups can affect the point of zero charge (PZC) of the nanoporous carbons and thus influence their performance in aqueous electrolytes,[10] these effects may be less significant in the organic electrolyte (1 M NEt_4BF_4 /ACN) used in the current study.[38]"

The lack of correlation between oxygen-containing functional groups and capacitance in organic electrolytes with aprotic solvents has been consistently observed from our previous studies with over 20 nanoporous carbons (*Science*, **2024**, *384*(6693),321-325), our recent preprint paper: https://chemrxiv.org/engage/chemrxiv/article-details/6911e072a10c9f5ca1910bb1 and studies from other groups (*J. Energy Chem.,* **2020** *51*, 396-404; *New Carbon Mater.,* **2020** *35*, 232–243). These findings suggest that oxygen-containing functional groups do not block the pores.

Additionally, there is no direct evidence of functional groups blocking the pores, as demonstrated by the lack of correlation between ion adsorption capacity measured by NMR and oxygen-containing functional groups from XPS measurements **(Figure R1).**

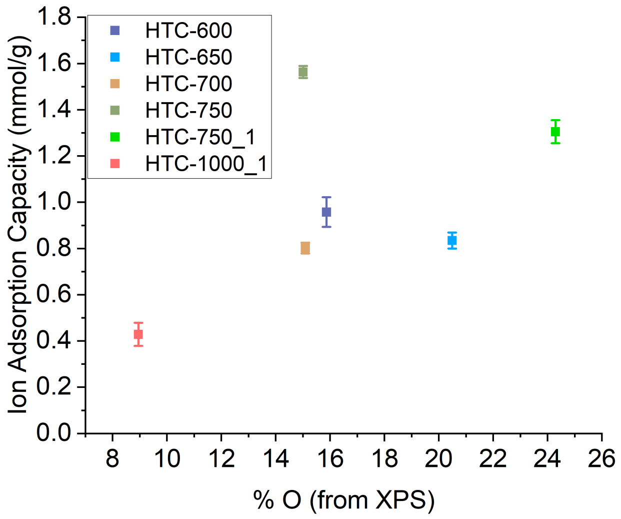

Figure R1: Relationship between oxygen-containing functional groups using XPS measurements and ion adsorption capacity from NMR spectra.

Another issue is the degree of disorder in carbon sample activated at 750°C compared to other samples. The significant difference is of course due to the higher temperature which is more favourable for introducing long-range graphene layers. Further high temperature such as 1000°C

or 1200°C would lead to graphitization of material which is generally used for producing hard carbons and is already a known fact. However, in the context of this manuscript, this abrupt change of structure for the sample produced at 750°C has not been explained sufficiently and scientifically.

The structure of all studied carbons has been characterised with NMR, Raman and gas physisorption techniques. Our NMR and Raman measurements characterise the structural order degree of the 750 °C sample. As shown in our original main text:

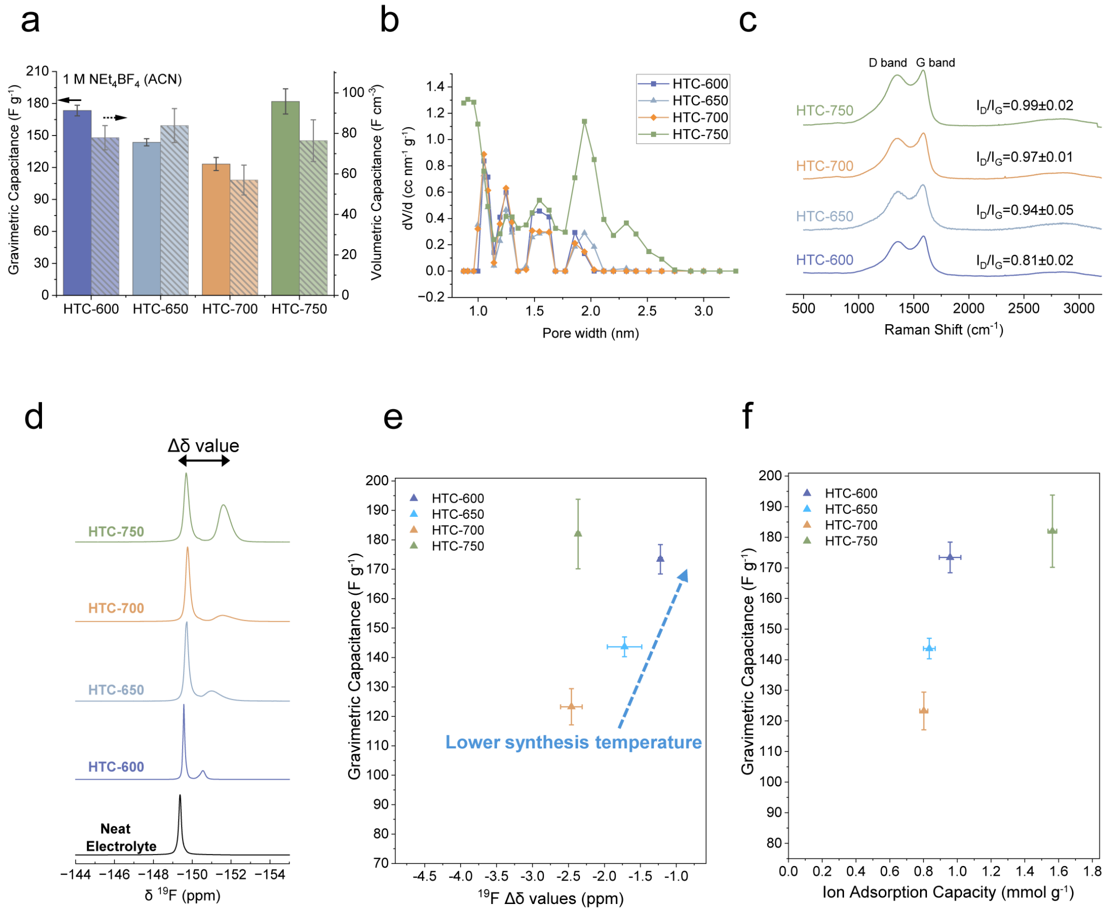

Figure 1. (a) Gravimetric and volumetric capacitances of low-temperature synthesised carbons measured at 0.05 A g^{-1} in 1 M NEt_4BF_4 /ACN, with the masses of the electrodes shown in Table S4. (b) Pore size distributions of low-temperature synthesised carbons calculated based on 2D non-local density functional theory (2D-NLDFT)[39] analysis (slit pore model) of N_2 isotherms at 77 K, with BET surface area, pore volume and average pore size presented in Table S1. (c) Raman spectra of low-temperature synthesised carbons (532 nm). See Supporting Information and our previous work[32] for details on deconvolution of Raman spectra. (d) ^{19}F MAS NMR spectra (14.1 T, 5 kHz MAS) of the synthesised carbons soaked with 1 M NEt_4BF_4 /ACN electrolyte. (e) Plot of gravimetric capacitance and ^{19}F Δδ values derived from d), with the in-pore chemical shifts taken as the weighted average for carbons showing multiple in-pore environments. (f) Relationship between gravimetric capacitance at 0.05 A g^{-1} in 1 M NEt_4BF_4 /ACN and ion adsorption capacity.

Our carbons are not "graphitised" as they are highly disordered activated carbons with small

graphene-like domains, which is one of the structural features that enhances the capacitance. HTC-750 shows a Δδ value of -2.37 ppm and an ordered domain area of around 49.2 Å^2 from our simulations with the discussed assumptions.

Regarding the "abrupt" change in porosity, during KOH activation, KOH initially reacts with carbon to form K_2CO_3, which subsequently reacts with additional carbon and decomposes to produce K_2O, CO, and CO_2. Therefore, the pores are formed based on three potential mechanisms: i) etching of the carbon framework by redox reactions, ii) physical activation by gaseous products, *e.g.* CO and CO_2 and iii) the intercalation of metallic potassium, which further expands the carbon lattices. At temperatures higher than 700°C, K_2CO_3 begins to decompose into K_2O and CO_2, resulting in dramatic pore opening and substantially increased BET surface areas.

As we have discussed extensively in the main text of the original manuscript,

"During the KOH activation process, KOH decomposes into K_2CO_3 around 400°C, which further breaks down into K_2O and CO_2 at temperatures above 700°C, leading to activated carbons with high BET surface areas and development of pores around 2 nm diameter.[17,18,20]"

"We attribute this to the significant pore opening at 750 ℃ which gives rise to the sample with the highest BET surface area (2689 m^2 g^{-1}) in the series and a significant number of pores below 1 nm in diameter and around 2 nm in diameter (Figure 1b, Table S1). This is likely due to the more dramatic etching of the carbon framework at 750 ℃ by KOH and the formation of potassium-containing compounds at this temperature (i.e. K_2CO_3 and K_2O).[17,18]"

This phenomenon is also extensively reported in previous literature (*Journal of Materials Chemistry*, **2012**, (*22*), 23710-23725; *Advanced Energy Materials*, **2011**, (1), 356-361)

Some suggestions on role of pore size distribution are listed below.

• From the pore size distribution data, authors could calculate the fraction of pore volume for micro-, ultra-micro-, and mesopores that would reveal whether improved capacitance arises primarily from structural disorder or from differences in accessible pore volume.

We have included the pore size distribution plots in the original manuscript. As shown in our original main text and Supporting Information:

From the main text **Figure 1**:

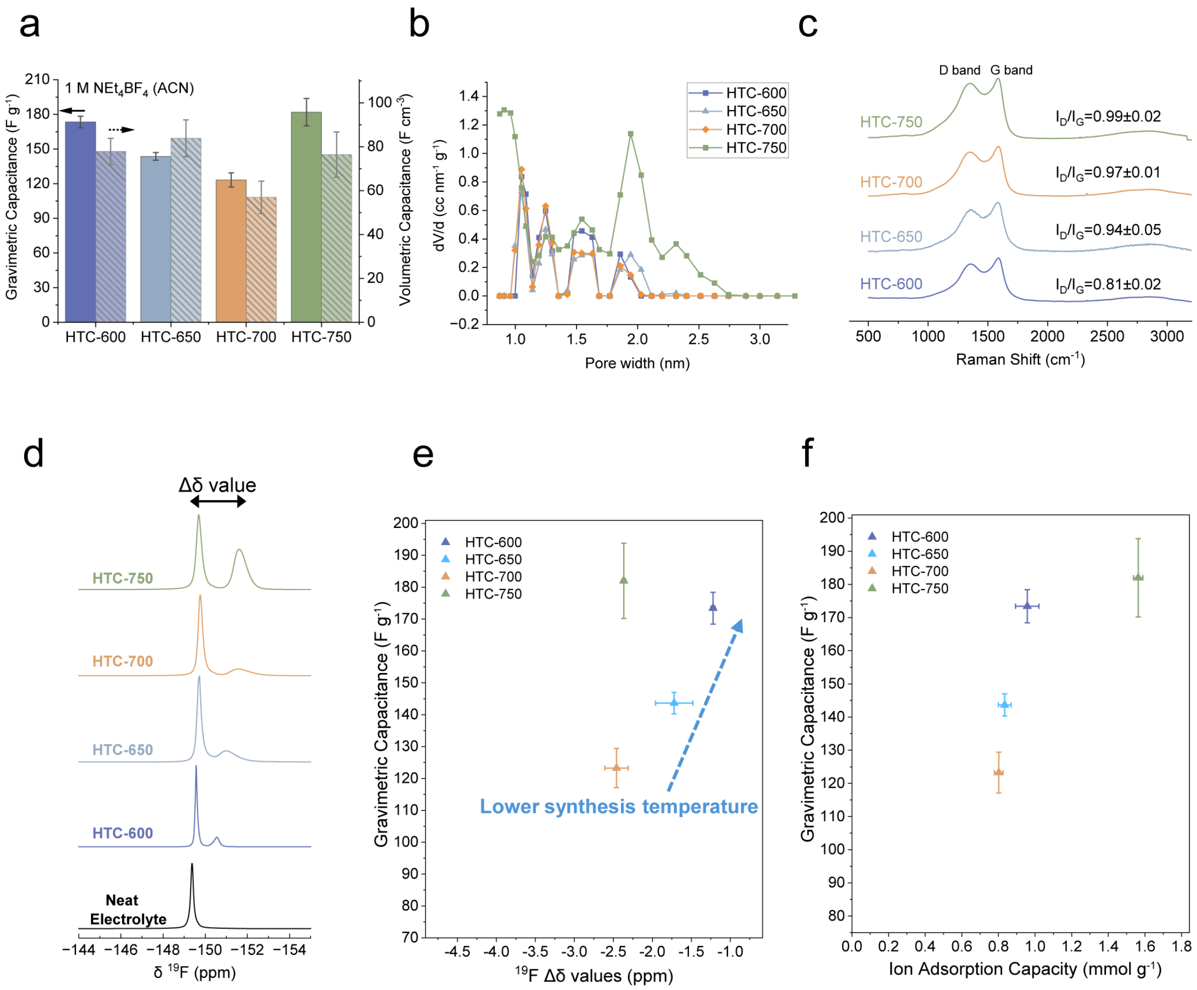

Figure 1. (a) Gravimetric and volumetric capacitances of low-temperature synthesised carbons measured at 0.05 A g^{-1} in 1 M NEt_4BF_4 /ACN, with the masses of the electrodes shown in Table S4. (b) Pore size distributions of low-temperature synthesised carbons calculated based on 2D non-local density functional theory (2D-NLDFT)[39] analysis (slit pore model) of N_2 isotherms at 77 K, with BET surface area, pore volume and average pore size presented in Table S1. (c) Raman spectra of low-temperature synthesised carbons (532 nm). See Supporting Information and our previous work[32] for details on deconvolution of Raman spectra. (d) ^{19}F MAS NMR spectra (14.1 T, 5 kHz MAS) of the synthesised carbons soaked with 1 M NEt_4BF_4 /ACN electrolyte. (e) Plot of gravimetric capacitance and ^{19}F $\Delta\delta$ values derived from d), with the in-pore chemical shifts taken as the weighted average for carbons showing multiple in-pore environments. (f) Relationship between gravimetric capacitance at 0.05 A g^{-1} in 1 M NEt_4BF_4 /ACN and ion adsorption capacity.

From the Supporting Information **Figure S3**:

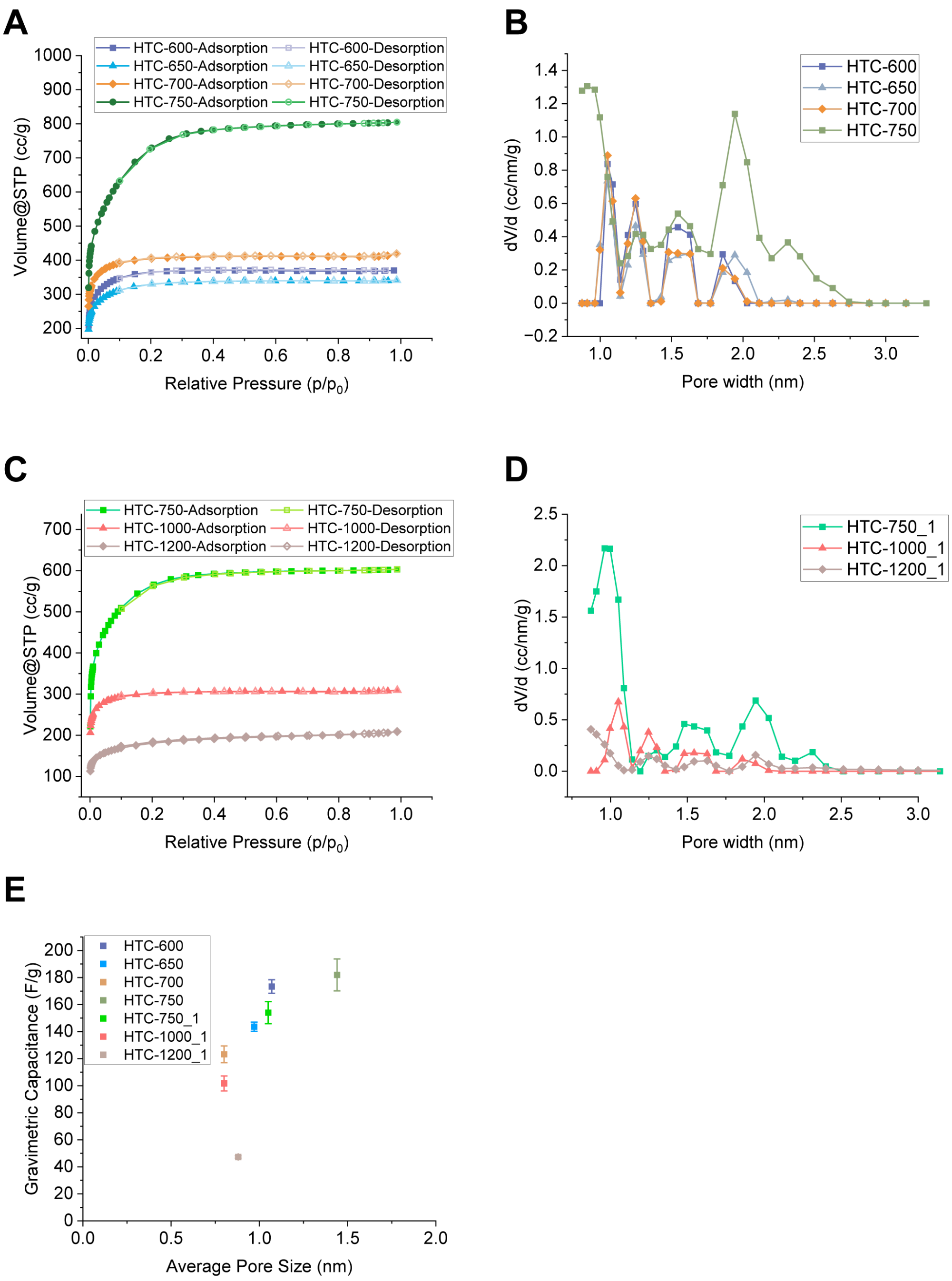

Figure S3: Gas sorption isotherms and pore size distribution plots of the synthesised carbons. *(**A**) N_2 isotherms at 77 K of the four synthesised nanoporous carbons (filled dots show adsorption, hollow dots show desorption). **(B)** Pore size distributions based on 2D-NLDFT analysis (slit pore model) of N_2 isotherms at 77 K in **(A)**.[1] (**C**) N_2 isotherms at 77 K of three synthesised nanoporous carbons (filled dots show adsorption, hollow dots show desorption). **(D)** Pore size distributions based on 2D-NLDFT analysis (slit pore model) of N_2 isotherms at 77 K in **(C)**.[1] **(E)** Relationship between gravimetric capacitances of the synthesised nanoporous carbons in 1 M NEt_4BF_4 /ACN and average pore sizes.*

In **Figure 1b** and **Figure S3B**, HTC-600, HTC-650, and HTC-700 clearly have similar PSD patterns, demonstrating a similar fraction of pore volume for micro-, ultra-micro-, and mesopores. However, these 3 carbons show significantly different gravimetric capacitance ranging from 173 to 124 F/g, directly contradicting any correlation between pore volume fractions and capacitance. The lack of correlation between capacitance and average pore size (**Figure S3E**) has been consistently observed across our previous study of over 20 nanoporous carbons (*Science*, **2024**, *384*(6693),321-325) and independently confirmed by other research groups (*Phys. Chem. Chem. Phys*., **2011** 13, 12403–12406).

More importantly, estimating ion accessible pores from gas physisorption measurements assumes that pores accessible to small gas molecules at measurement temperature (e.g., 77 K for N_2) are equally accessible to electrolyte ions during electrochemical processes at room temperature. Our NMR-derived ion adsorption capacity directly measures electrolyte ion accessibility to carbon pores in the absence of applied potential, providing a more powerful predictor of electrochemical performance than BET surface area or pore size analysis.

We have addressed this point in the revised manuscript:

From the revised manuscript:

"*Additionally, the integral of the in-pore resonance quantifies the ion adsorption capacity within nanopores in the absence of applied potential. This approach provides a more direct measure of ion accessible porosity than gas physisorption analysis, as the latter relies on the assumption that pores accessible to small gas molecules at measurement temperatures (e.g., 77 K for N_2) are equally accessible to the larger electrolyte ions at room temperature.*"

• ***To what extent might pore size distribution influence the NMR-derived descriptors (Δδ and ion adsorption capacity)? Smaller pores may enhance adsorption capacity but limit ion mobility; conversely, larger pores may facilitate faster transport but reduce volumetric performance. These effects could influence the interpretation of the NMR data.***

The Δδ values are affected by the graphene-like domain sizes and to a lesser extent the pore size for predominantly microporous carbons. Our simulation decouples the pore size domain size effects from pore size effects. The interpretation of the graphene-like domain sizes is independent of the pore sizes. As described in our original main text:

"Our previously reported NMR simulation approach[21,22,30] provides a simple method to model these effects, accounting for both the pore sizes and ion-carbon interactions, therefore allowing a representative graphene-like domain size to be extracted from the NMR spectrum."

As described in our original main text:

"Importantly, in addition to providing a measurement of the sizes of the graphene-like domains, our NMR experiments measure another key property of the nanoporous carbons; the electrolyte ion adsorption capacity. Indeed, the integrals of the in-pore resonances relate to the number of ions that can access the carbon nanopores in the absence of applied potential."

The ion adsorption capacity is a direct probe of ion accessible porosity with the electrolyte ions

being the probing molecules and is not necessarily correlated with the porosity measured from gas physisorption techniques. From our original Supporting Information **Figure S7C**:

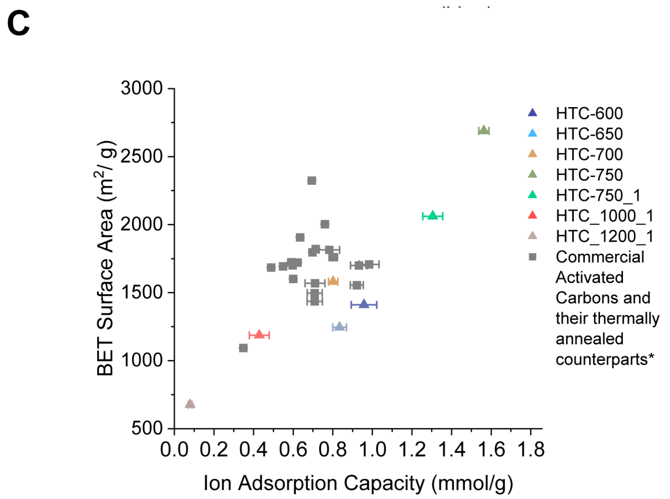

Figure S7: Relationship between volumetric capacitance and ion adsorption capacity. (C) *Relationship between BET surface area and ion adsorption capacity for the synthesised carbons series, with commercial activated carbons and their thermally annealed counterparts data added from previous studies.[2] For carbons with extreme surface areas - very low (HTC-1200, ~676 m^2/g) and very high (HTC-750, ~2689 m^2/g) - BET surface area correlates well with ion adsorption capacity. However, for the majority of carbons clustering in the intermediate range (1400-2000 m^2/g), BET surface area shows no clear correlation with ion adsorption capacity, indicating that BET surface area alone is insufficient to predict electrochemically accessible porosity.*

• Are the observed performance differences attributable solely to graphene-like domain size, or could pore size and connectivity be co-contributing factors? Without PSD data, it is difficult to disentangle the relative contributions of domain disorder versus pore structure.

First, we have provided the PSD results for all the carbons studied in our original main text **Figure 1b** and Supporting Information **Figure S3B**. Please see our response to the previous comments.

Our previous work discovered that graphene-like domain sizes dominate capacitance in nanoporous carbons. With a wider range of carbons, here we reveal that both graphene-like domain sizes and ion adsorption capacities (as measured by NMR) determine capacitance, providing a more complete mechanistic understanding, which has not been reported in previous literature and extends the conclusion in our previous study.

As described in our original manuscript:

In abstract: *"NMR spectroscopy measurements provided unique insights by simultaneously probing local structural order and ion adsorption capacities, revealing that carbons with smaller graphene-like domain sizes and higher ion adsorption capacities generally have better capacitive performance."*

In the main text: *"Importantly, in addition to providing a measurement of the sizes of the graphene-like domains, our NMR experiments measure another key property of the nanoporous carbons; the electrolyte ion adsorption capacity. Indeed, the integrals of the in-pore resonances relate to the number of ions that can access the carbon nanopores in the absence of applied potential. Deconvolution of the NMR spectra of the electrolyte-saturated carbon samples shows that the gravimetric capacitance showed some correlation with the ion adsorption capacity (Figure 1f), with HTC-750 having the largest ion adsorption capacity in the series (1.6 mmol g^{-1})."*

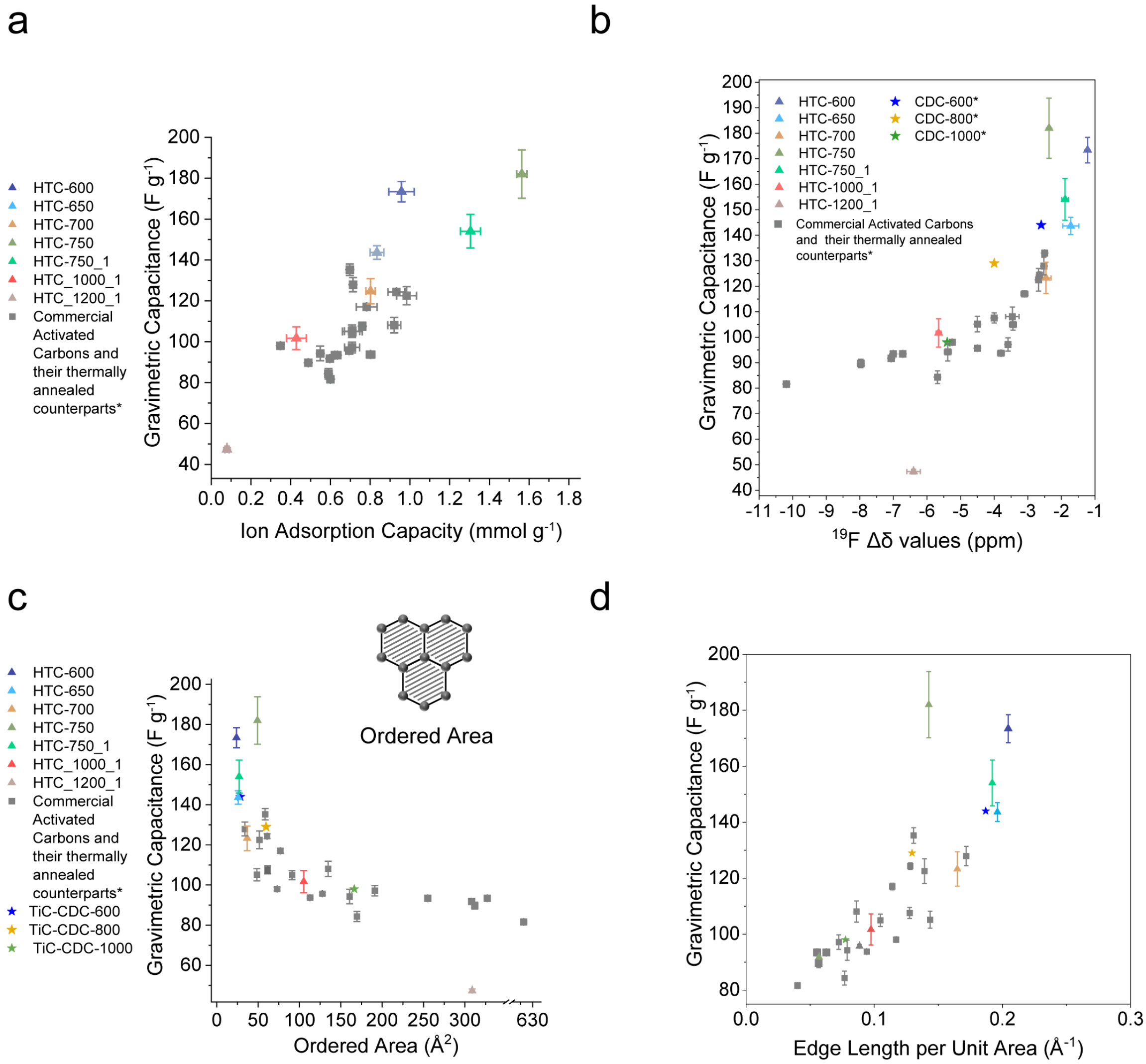

Figure 3. (a) Correlation between gravimetric capacitance and ion adsorption capacities, with commercial activated carbons, their thermally annealed counterparts data added from previous studies.[3,21] See Figure S7 for the relationship between ion adsorption capacities and BET surface areas. (b) Correlation between gravimetric capacitance and ^{19}F Δδ values, with commercial activated carbons, their thermally annealed counterparts and CDC data added from previous studies.[3,21] (c) Relationship between gravimetric capacitance and calculated ordered domain size of the synthesised nanoporous carbons, with commercial activated carbons, their thermally annealed counterparts and CDC data added from previous studies.[21] (d) Correlation between gravimetric capacitance and edge length per unit area for synthesised carbons, with commercial

activated carbons and their thermally annealed counterparts data added from previous studies.[21] *See Methods for detailed definitions of the edge length per unit area.*

"With an expanded series of nanoporous carbons including those from our previous studies, it is observed that carbons with higher gravimetric capacitances generally have (i) larger ion adsorption capacities (Figure 3a) and (ii) Δδ values of smaller magnitude (Figure 3b), which identifies ion adsorption capacity as another key descriptor for capacitance and extends our previous findings.[21]*"*

In conclusion: *"NMR spectroscopy, in particular, allowed us to simultaneously evaluate both the sizes of the graphene-like domains and the electrolyte ion adsorption capacities, with electrolyte ions as the probe. Our findings indicate that carbons with smaller graphene-like domains and higher ion adsorption capacity generally show enhanced capacitive performance. The highest gravimetric capacitances were achieved for either highly disordered carbons or carbons with more order but more accessible pores (higher electrolyte ion adsorption capacities), the latter carbons, however, generally being associated with lower volumetric capacitances. We show that the combination of Δδ and the ion adsorption capacity both measured by NMR spectroscopy in a quick and simple NMR experiment (~5 mins) are excellent descriptors for capacitance, enabling rapid identification of high-performance materials without electrochemical testing"*

• How does the pore size distribution of these materials relate to tortuosity metrics discussed in their earlier Nature Materials study? For example, carbon with high microporosity but lower mesopore volume might inherently exhibit higher tortuosity, which could influence rate capability even if intrinsic capacitance is high.

Tortuosity is an independent descriptor of kinetic performance and is not necessarily correlated with pore size but is instead correlated with connectivity of the pores, as discussed in the *Nature Materials* study. We have included the rate performance of the studied carbons in the original **Supporting Information**:

From the original Supporting Information **Figure S2A** and **B**:

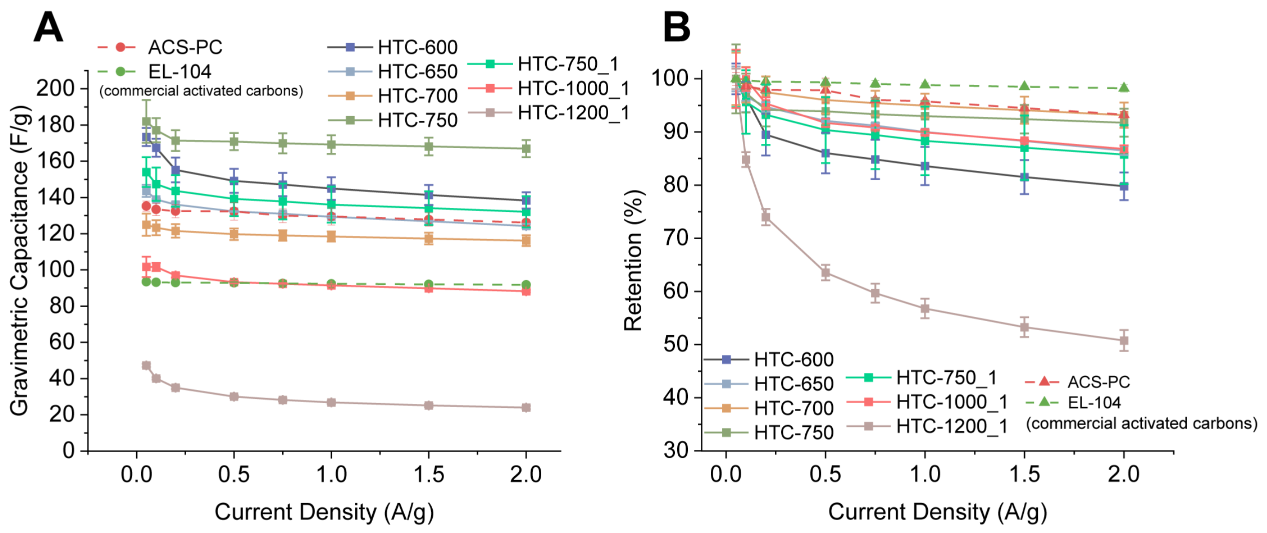

Figure S2: Gravimetric capacitances at different current densities and cyclability of the synthesised carbons. *(A) Gravimetric capacitances of the synthesised and two commercial nanoporous carbons in 1 M NEt_4BF_4 /ACN at different current densities.* ***(B)*** *Capacitance retention ratio of the synthesised and two commercial nanoporous carbons and in 1 M NEt_4BF_4 /ACN at different current densities, with the retention ratio calculated by gravimetric capacitance at x current density divided by the gravimetric capacitance at 0.05 A g^{-1}.*

HTC-1200 shows the worst retention among all synthesised nanoporous carbons, which potentially has a more tortuous structure. However, this current work focuses on establishing the fundamental thermodynamic relationship between structural parameters (*i.e.* graphene-like domain sizes and ion adsorption capacities) and capacitance and proving the concept that low-temperature synthesis is a promising path to engineer carbons with enhanced capacitance. Detailed kinetic analysis including tortuosity and electrolyte ion diffusion studies would be an interesting avenue for future work, although it is ultimately beyond the scope of our current study.

• ***Correlating the authors finding with pore size distribution of carbons preferably would allow readers to better align the present results with the established understanding of pore-network effects from the author's earlier work. This would also strengthen the mechanistic interpretation of the structure–property relationships and provide a more complete picture of how disorder, pore accessibility, and pore geometry collectively determine capacitive performance.***

Our comprehensive analysis of over 20 nanoporous carbons showed no correlation between average pore size and capacitance. Instead, we demonstrated a strong correlation between capacitance, graphene-like domain sizes and ion adsorption capacities.

From the original main text:

"With an expanded series of nanoporous carbons including those from our previous studies, it is observed that carbons with higher gravimetric capacitances generally have (i) larger ion adsorption capacities (Figure 3a) and (ii) $\Delta\delta$ values of smaller magnitude (Figure 3b), which extends our previous findings.[21]"

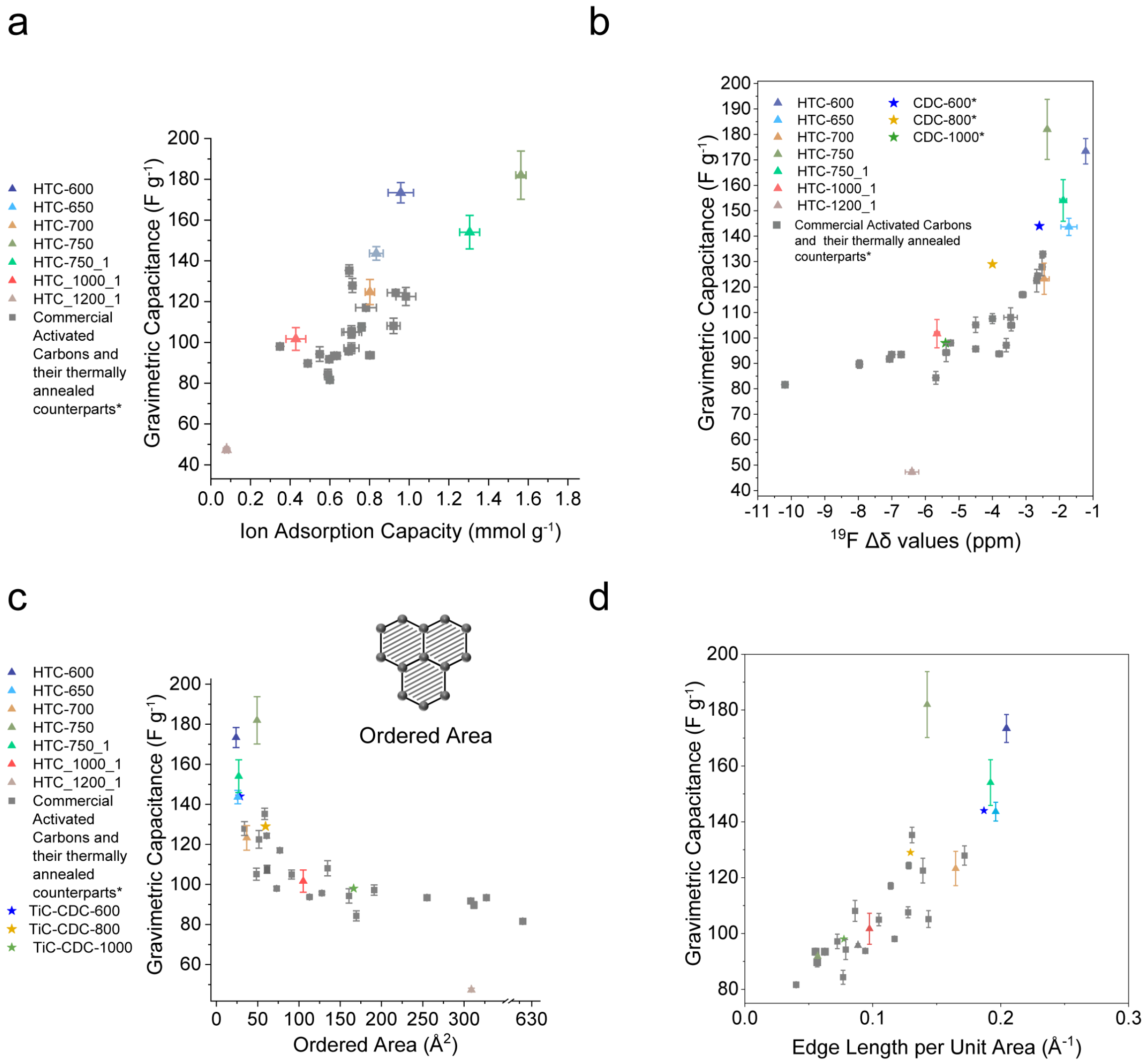

Figure 3. (a) Correlation between gravimetric capacitance and ion adsorption capacities, with commercial activated carbons, their thermally annealed counterparts data added from previous studies.[3,21] *See Figure S7 for the relationship between ion adsorption capacities and BET surface areas. (b) Correlation between gravimetric capacitance and* ^{19}F *Δδ values, with commercial activated carbons, their thermally annealed counterparts and CDC data added from previous studies.*[3,21] *(c) Relationship between gravimetric capacitance and calculated ordered domain size of the synthesised nanoporous carbons, with commercial activated carbons, their thermally annealed counterparts and CDC data added from previous studies.*[21] *(d) Correlation between gravimetric capacitance and edge length per unit area for synthesised carbons, with commercial activated carbons and their thermally annealed counterparts data added from previous studies.*[21] *See Methods for detailed definitions of the edge length per unit area.*

From our original conclusion:

"*Our findings indicate that carbons with smaller graphene-like domains and higher ion adsorption capacity generally show enhanced capacitive performance. The highest gravimetric capacitances were achieved for either highly disordered carbons or carbons with more order but more accessible*

pores (higher electrolyte ion adsorption capacities), the latter carbons, however, generally being associated with lower volumetric capacitances."

Authors should show the morphology of these carbons such as with TEM data to clarify how the graphene domains are oriented in each carbon sample. Such data also helps to calculate adjacent graphene layer distance and their orientation. In some of the works given below, it is clearly shown how the graphene layers are arranged leading to the pore development.

https://www.sciencedirect.com/science/article/abs/pii/S0008622320307399 Melting point depression of ionic liquids by their confinement in carbons of controlled mesoporosity - ScienceDirect https://link.springer.com/article/10.1007/s11581-021-04354-w Revisiting the carbon mesopore contribution towards improved performance of ionic liquid–based EDLCs at sub-zero temperatures | Ionics

A similar data would definitely help in understanding the organization of graphene domains and pore development by the short-range and long-range graphene layers which is important for finding a correlation with how the charges are stored and how capacitance increases.

The morphology and porosity of these materials have been extensively studied in previous studies (*Carbon*, **2013**, *62*, 346-355). TEM studies have revealed that nanoporous carbons comprise three-dimensional networks of wrinkled graphene sheets with non-hexagonal defects (*Small*, **2012,** *8,* 3283-3288), but direct imaging provides only localised evidence for topological defects without quantitative information. Our NMR measurements, combined with simulations and Raman spectroscopy, quantitatively analyse the ion adsorption capacity and degree of structural disorder, using disorder metrics including the Δδ values, ordered areas, and I_D/I_G values. These metrics consistently correlate with capacitance across nanoporous carbons from diverse sources, indicating that both ion adsorption capacity and graphene-like domain size are key descriptors of capacitance. TEM analysis would not enhance our conclusions.

At the reviewer's comments, we have included previous TEM studies in the revised manuscript:

From the revised manuscript: "*While TEM has been used to visualise topological defects in disordered carbons,[35] such imaging provides only localised views without the quantitative disorder metrics essential for establishing structure-performance correlations.*"

It would be unfair to rule out the role of pore size in the electrochemical performance of supercapacitors without presenting three-electrode cell data. The electrochemical performance lacks information on individual carbon electrodes in the given organic electrolyte where one could see the charge/discharge behaviour of carbon electrodes on positive and negative sides of the supercapacitor cell to clarify how the size of pores affects the capacitances on each polarity.

First, we have not completed rule out the role of pore size in the electrochemical performance of supercapacitors. Our predictive model shows that the Δδ value and ion adsorption capacity from the NMR spectrum can predict the capacitance. As shown in our original manuscript, and from the

original main text:

"Mapping ion adsorption capacity, Δδ values and gravimetric capacitance for a wide range of nanoporous carbons, we propose that the capacitance can be directly predicted from the ion adsorption capacity and the Δδ value, both measurable in a single NMR experiment. (Figure 4b)."

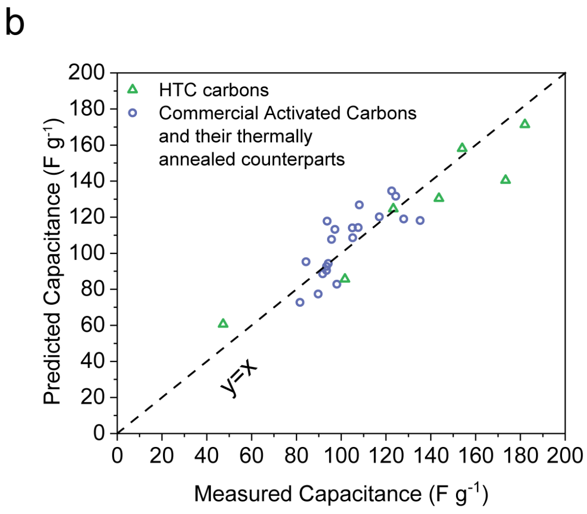

Figure 4. (b) Relationship between predicted capacitance and measured capacitance using Δδ values and ion adsorption capacity measured from NMR experiments.

The porosity influences the ion uptake capacity, which is best measured directly via NMR spectroscopy on electrolyte-soaked samples, rather than through gas physisorption analysis. As we have addressed in the previous comments.

Second, our results clearly demonstrate no correlation between pore size and capacitance across a comprehensive series of synthesised and commercial nanoporous carbons (*Science*, **2024**, *384*(6693),321-325) in two-electrode symmetric cells. We have conducted three-electrode cell measurements on two commercial nanoporous carbons with different graphene-like domain sizes, which confirmed that capacitance is similar for both positive and negative electrodes for a given carbon **(Figure R2)**. These three-electrode results validate that our disorder-capacitance relationships hold regardless of electrode polarity, and that a symmetric two-electrode configuration provides a reasonable assessment of structure-performance relationships.

Figure Redacted

Figure R2: Gravimetric capacitance of ACS-PC and EL-104 calculated from constant charge-discharge measurements at 0.05 A/g in a three-electrode cell in 1 M $TEABF_4$ (ACN). Data from https://chemrxiv.org/engage/chemrxiv/article-details/6911e072a10c9f5ca1910bb1
